# Preparation and Surface Properties Study of Novel Fluorine-Containing Methacrylate Polymers for Coating

**DOI:** 10.3390/ma11112258

**Published:** 2018-11-13

**Authors:** Ding Zhang, Ping Xing, Renming Pan, Xiangyang Lin, Min Sha, Biao Jiang

**Affiliations:** 1 School of Chemical Engineering, Nanjing University of Science and Technology, Nanjing 210094, China; zhangdingkathy@163.com (D.Z.); panrenming@163.com (R.P.); linxiangyang@njust.edu.cn (X.L.); 2 CAS Key Laboratory of Synthetic Chemistry of Natural Substances, Shanghai Institute of Organic Chemistry, Chinese Academy of Sciences, Shanghai 200032, China; xingping@mail.sioc.ac.cn; 3 School of Management Science & Engineering, Nanjing University of Finance & Economics, Nanjing 210046, China

**Keywords:** fluorocarbon coating, fluorine-containing methacrylate, free radical copolymerization, repellency, thermal property

## Abstract

A new structural fluorine-containing methacrylate monomer CH_2_=C(CH_3_)COOC–(CF_3_)_2_CF_2_CF_2_CF_3_ (**5**) was synthesized derived from perfluoro-2-methyl-2-pentene (D_2_). A homopolymer of **5** and copolymers of **5** and methacrylate with different alkyl chain length (chain length *n* = 1, 2, 4, 6, 8, 12, 18) were obtained. These new fluorinated acrylate polymers showed excellent water and oil repellency. The contact angle of the films of the homopolymer and part of the copolymers were similar with the corresponding polymers prepared from CH_2_=CHC(O)OCH(C_3_F_7_)(CF(CF_3_)_2_), but greater than that of the C_6_F_13_(CF_3_)CHOC(O)CH=CH_2_ homopolymer. The structure-property relationship research indicated that the copolymers’ hydrophobicity decreased first and then increased with the increase of alkyl chain length. *T*_d_ of all the polymers were greater than 220 °C and *T*_g_ fluctuated within the range of −51~103.8 °C. Contact angle and *T*_g_ could be adjusted by controlling the feed ratio of monomer to meet the requirements of technical indicators in the practical applications. The outstanding liquid repellency and thermal stability make monomer **5** a promising alternative to perfluorinated long-chain fluorosurfactants.

## 1. Introduction

The fluorine-containing acrylate polymer is an excellent coating film material whose side chains are usually composed of perfluorinated chains. In practical applications, the fluoroalkyl chain will migrate to the surface of the film, which endows it with excellent abrasion resistance, corrosion resistance, weather resistance, water and oil repellency [1,2,3]. It is regarded as the mainstream of today’s high-grade coating materials, which has been widely used in textiles, coatings, leather, paper and other fields [4].

Generally, the long perfluoroalkyl chain (C6–C12) exhibits excellent performance of coating, and the most widely used coating is C8 perfluoroalkyl chain consider of cost and performance [5]. However, upon the strengthening of environmental protection awareness, these compounds have been listed as one of the most difficult organic pollutants to degrade [6,7]. Because when organisms ingest perfluorooctanoic acid (PFOA, C_7_F_15_COOH) or perfluorooctane sulphonate (PFOS, C_8_F_17_SO_3_X, with X = K, Na, H), they are difficult to decompose through metabolism due to their inherent stability [8,9]. Many countries have gradually enacted agreements to ban the use of fluorosurfactants with a long fluorocarbon chain length (≥C8) [10]. Therefore, it is increasingly urgent to develop novel environmentally friendly alternatives to perfluorinated long-chain fluorosurfactants (PFOS/PFOA) and evaluate their biodegradability and environmental impact at the same time.

Recently, the short-term fluorocarbon chain (≤C6) has been developed to achieve a certain degree of water and oil repellency [11,12]. Although its water-repellent property satisfies the requirements, its oil repellency is worse than that of the long fluorocarbon chain. In view of the deficiencies in the current development, production and application of fluorocarbon polymers [13,14,15,16], our group successfully developed a series of fluorinated acrylate polymers based on reactive sites of carbon-oxygen double bond of perfluoro(2-methyl-3-pentanone) [17]. Their performances were greater than that of C_6_F_13_(CF_3_)CHOC(O)CH=CH_2_ polymer, slightly worse than that of C_8_F_17_CH_2_CH_2_OC(O)CH=CH_2_ polymer. These results demonstrate that the branched fluorinated surfactants show more efficiency than straight chain fluorinated surfactants.

Fluorocarbons with CF_3_CF_2_CF_2_C(CF_3_)_2_– group can be obtained using perfluoro-2-methyl-2-pentene as raw material. Structurally, the fluorinated chain of these compounds is the structure of C_4_ and two groups of CF_3_ are located in the chain as branches. Many surfactants containing CF_3_CF_2_CF_2_C(CF_3_)_2_– groups have been made by our group. They showed higher efficiency in a relatively low concentration. Surface tension of aqueous solution (γ_cmc_) and critical micelle concentration (CMC) of these solutions were much lower than those of PFOA (CMC = 31.2 mmol·L^−1^, γ_cmc_ = 24.7 mN·m^−1^), which showed better surface performances than those of PFOA [18,19,20].

Characteristic wetting behavior of solid surfaces is governed by both chemical composition and the geometrical microstructure of the surface [21,22]. Fluorine-rich polymers possess low surface energy to a mass of substrates and, thus, endow the treated surface with excellent water and oil repellency [17,23]. Furthermore, their feature advantages like corrosive liquid resistance, optical transparency, excellent mechanical property and stable weatherability make them appealing candidates for fabricating hydrophobic surface [24]. Among the diverse fluorinated polymers, fluorinated acrylate copolymer has drawn much attention due to their high hydrophobicity, good film-forming properties and excellent adhesion on various substrates [25]. So far, numerous approaches have been reported to improve surface roughness, such as phase separation [26,27], plasma etching [28], sol-gel processes [29,30], electrochemical deposition [31], spray-casting [32] and template methods [33,34]. However, most of these fabrication methods require multiple fabrication steps and use flammable or hazardous solvents, thus limiting the large-area applications of them.

Given the above conclusions, novel fluorine-containing acrylate polymers were designed and synthesized from perfluoro-2-methyl-2-pentene based on fluoro-olefin double bond reactive site, which was regarded less toxic, and hence, minimizing bio-accumulation risks associated with C-8 fluoro-compounds [18,19,20]. The repellency and thermodynamic properties of the polymers were investigated to explore the relationship between structure and performance. This work aims to enrich experimental and theoretical foundations for the development and application of fluorinated coating materials. 

## 2. Experimental

### 2.1. Materials

Methyl methacrylate (MMA), ethyl methacrylate (EMA), butyl methacrylate (BMA), hexyl methacrylate (HMA), iso-octyl methacrylate (OMA), lauryl methlacrylate (LMA), stearyl methacrylate (SMA), methacryloyl chloride, and triethylamine were purchased from Aladdin (Shanghai, China). Perfluoro-2-methyl-2-pentene (D_2_) was supplied by Shanghai Aimojinshan Pharmaceutical Company (Shanghai, China). Ethyl ether and toluene were distilled from sodium wire containing benzophenone, methacrylate monomers were washed with 5 wt % NaOH solution and dried. 2,2-Azobisisobutyronitrile (AIBN) was recrystallized from ethanol. All chemical reagents were of AR grade quality. Solvents were of AR grade quality and dried by the standard methods before use. 

### 2.2. Synthesis of Monomer **5**

#### 2.2.1. Synthesis of Perfluoro-2-methyl-2-pentanol (**4**) 

5 g (0.86 mol) of dry KF was added to 100 mL three-necked flask and the system was purged for three times. Then, 50 mL of *N*, *N*-dimethylacetamide (DMA_C_) and 17.13 g (0.0571 mol) of D_2_ were added in order. The reaction system was stirred in an ice–salt bath (keep the temperature below −7 °C) for 1 h while N_2_O_4_ (10.9 g, 0.118 mol) was introduced under a slight flow of O_2_. A condenser at 78 °C (dry ice-ethanol bath) was used to prevent the overflow of N_2_O_4_ due to the exothermic effect of system. Immediately after addition the reaction system turned dark green, NMR spectra showed that **2** was generated. D_2_ reacted completely, and the dark green disappeared after 18 h. O_2_ was slowly introduced at room temperature for 48 h. NMR spectra showed the formation of Compound **3**, whereupon the reaction system gradually turned pale yellow. 0.62 g of H_3_PO_3_ and 40 mL of water were added to the mixture. A small amount of sulfamic acid was slowly added dropwise to the system to remove excess NO_2_. The mixed system was distilled at atmospheric pressure. Excess KOH was added to the fraction, and it was subjected to vacuum distillation at atmospheric pressure. A small amount of undissolved fraction was distilled into −78 °C of trap, and then the remaining fractions was acidified with H_2_SO_4_ (1 mol/L). The liquid was separated, mixed with 5 mL of H_2_SO_4_ (98%). Compound **4** (12.129 g, yield = 65.7%, purity of 99%) was distilled by vacuum pump into a dry ice cooled trap [35]. ^1^H NMR (400 MHz, DMSO-d6, δ): 10.96 (*d*, *J* = 4.3 Hz, 1H); ^19^F NMR (376 MHz, DMSO-d6, δ): −71.47~−71.51 (m), −80.09 (*d*, *J* = 11.7 Hz), −115.44 (s), −124.05 (s); LRMS (EI), *m*/*z* (%): 69.1(100), 297.0(36.9).

#### 2.2.2. Synthesis of 1,1,1,3,3,4,4,5,5,5-decafluoro-2-(trifluoromethyl)pentan-2-methacrylate (**5**)

The 50-mL jacketed reaction flask was purged for three times using Ar. Then, 4 g (11.9 mmol) of Compound **4**, 2.64 mL (14.75 mmol) of triethylamine and 10 mL of ether were added by sequence. The mixture was stirred in the dry ice-ethanol bath for 2 h, followed by the addition of 1.49 g (14.28 mmol) of methacryloyl chloride at about 0.5 h. The reaction was performed at room temperature overnight. The mixture was filtrated, which was washed by 3% of KHCO_3_, 5 mol/L of hydrochloric acid and saturated NaCl solution for several times. The organic phase was dried by sodium sulfate and the solvent was removed. The mixture was distilled under pressure (8 kPa, 72 °C) to give the target fraction **5** (4.71 g, yield = 98.17%, purity of 99.5%) as colorless liquid. ^19^F NMR (376 MHz, CDCl_3_) δ: −66.08~−66.35 (m), −80.49 (*t*, *J* = 12.4 Hz), −112.57~−113.13 (m), −124.11, −124.73 (m). ^1^H NMR (400 MHz, CDCl_3_) δ: 6.22 (*d*, *J* = 0.7 Hz, 2 H), 5.82 (*t*, *J* = 3.4 Hz, 2 H), 1.98 (*dd*, *J* = 6.8, 0.7 Hz, 6 H). ^13^C NMR (101 MHz, CDCl_3_) δ: 159.97, 133.87, 130.06, 17.99. IR (cm^−1^): 1789.5, 1247.0, 1119.4, 1078.7, 995.9, 736.2, 720.0; LRMS (EI), *m*/*z* (%): 69.1(100), 404.1(29.2); HRMS (EI), *m*/*z* (100%): calcd for C_10_H_5_O_2_F_13_ 404.0082, found 404.0077.

### 2.3. Synthesis of Homopolymer (**6**)

In a 10-mL sealed tube, 7.5 mg (0.01 eq) of AIBN and 1 g (7.8 mmol, 1.0 eq) of Compound **5** were added. The oxygen in the system was purged with argon and sealed. The reaction was placed at 80 °C for 24 h. Then, the system was poured into methanol and repeatedly sedimented to precipitate the solid. The final product was filtered and dried to give the white powdery Product **6** (0.792 g, yield = 79.2%) Analytical calculated for C_10_H_5_F_13_O_2_: C 29.72, H 1.25, F 61.11, found: C 30.06, H 1.51, F 59.91. IR (cm^−1^): 3003.6, 1804.7, 1265.6, 1225.3, 1122.5, 1100.7, 735.8, 718.6.

### 2.4. Synthesis of Copolymers

Free-radical polymerization of the monomers was performed using AIBN as initiator in toluene at 80 °C. The equal amounts of monomers, solvents and initiators were loaded into the polymer tube and then immersed in a Dewar bottle containing liquid nitrogen. After three rounds of freezing and thawing under Ar, the tube was sealed and placed in an oil bath at 80 °C for 48 h. The polymer was purified twice by precipitation into methanol and dried in vacuum.

### 2.5. Preparation of Polymer Film

Glass slides of 15 mm × 15 mm × 1 mm were cleaned three times with ultrasonic wave in acetone solvent. Then, each piece was soaked in 1% hydrochloric acid solution for 30 min, rinsed with distilled water for 10 min, washed with absolute ethanol three times and dried. The polymer was dissolved in trichlorotrifluoroethane to prepare the coating solution of 3 wt % concentration and allowed to stand for 1 h. The pretreated clean glass slides were placed in the coating solution for a moment, then slowly and smoothly removed from the solution and placed at room temperature for 48 h. The solvent naturally evaporated to form the film. Six diaphragms were prepared for each solution to measure the contact angle of water and hexadecane. Every sample was measured three times and averaged.

### 2.6. Characterization

Molecular weight (*M*_n_) and molecular weight distribution (*M*_w_/*M*_n_) were determined by WATERS515 (GPC, Agilent, Palo Alto, CA, USA) using tetrahydrofuran (THF) as the eluent at a flow rate of 1.0 mL/min.

^1^H NMR, ^13^C NMR, and ^19^F NMR spectra were determined on a Brucker AC 400 spectrometer (Bruker, Madison, MA, USA), using CDCl_3_ as a solvent.

IR spectra were measured using Nicolet 380FT-IR instrument (Nicolet, Madison, MA, USA) and was reported in wave-numbers (cm^−1^). LRMS was performed on Agilent 5973 N mass instrument (EI, Agilent, Palo Alto, CA, USA). HRMS was performed on Waters Micromass GTC Premier (EI, Waters, Milford, MA, USA).

The glass transition temperature (*T*_g_) of polymers was measured using a DSC 2920 module (TA Instruments, Delaware, New Castle, DE, USA) with the TA (thermal analyser) Instrument 5100 system, with a scan rate of 10 °C/min. *T*_g_ was taken in the second heating scan as the midpoint of the heat capacity transition between the upper and lower points of deviation from the extrapolated liquid and glass lines.

A simultaneous DSC-TGA instrument (Netzsch, Bayern, Germany) and was used to characterize the decomposing temperature (*T*_d_). The temperature range was from 20 to 600 °C at a heating rate of 15 °C/min with N_2_ protection. 

Contact angles were measured on a Drop Shape Analysis System DSA100 (Krüss GmbH, Bayern, Germany) at room temperature.

The morphology of the films was carried out by a scanning electron microscopy (SEM) (JSM-6390LV, JEOL, Tokyo, Japan) or field emission scanning electron microscopy (FE-SEM) (NANOSEM450, FEI, Hillsboro, OR, USA). 

Atomic force microscopy (AFM) images were taken by a Bruker Dimension FastScan Veeco DI MultiMode SPM (Bruker, Madison, MA, USA) in the tapping mode of the film on the glass substrate.

X-ray photoelectron spectroscopy (XPS) was recorded on a 250XI ESCALAB photoelectron spectrometer (Thermo Fisher Scientific, Waltham, MA, USA).

## 3. Results and Discussion

### 3.1. Synthesis Analysis 

**Scheme 1:**
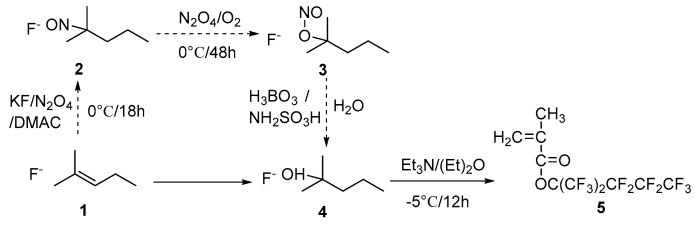
The synthetic route of the fluorine-containing acrylate monomer **5** based on D_2_.

The synthesis of perfluoro-2-methyl-2-pentanol (**4**) was shown in Scheme 1. Perfluoro-2-methyl-2-pentanol was carried out by a one-pot method from an inexpensive starting material (D_2_) and common reagents. Then, fluorinated alcohol was converted into an unsaturated ester (monomer **5**) by a condensation reaction with methacryloyl chloride. All the reactions were mild, easy-handled and cheap. Besides, the target product had low cost and high yield, which was promisingly applied to the industrial production.

**Scheme 2:**
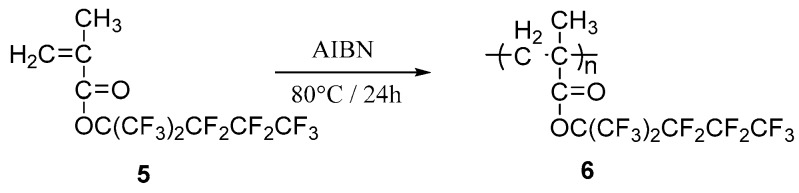
Preparation of homopolymer **6**.

Homopolymer of **5** was successfully synthesized under neat conditions (Scheme 2). This route was environmentally friendly and economical. However, homopolymers were insoluble in conventional solvents, so GPC characterization data cannot be obtained.

**Scheme 3:**
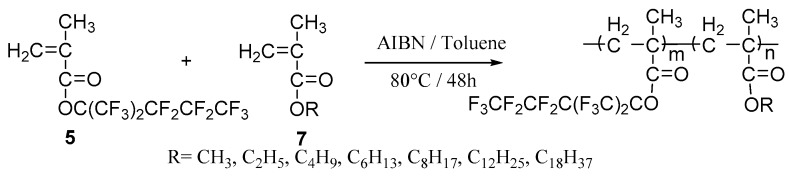
The synthetic route of the fluoroacrylate copolymer.

For oil- and water-repellent applications, compounds should be most conveniently used in an aqueous composition. In order to improve the solubility of polymers, methacrylate **7** with different alkyl chain length (chain length *n* = 1, 2, 4, 6, 8, 12, 18) were chosen to copolymerize with **5**. In previous studies, our group found that when the following polymerization conditions were selected: 20% solution in toluene, 0.03 equiv. of AIBN, and 2 h, a high yield and low *M*_w_/*M*_n_ of copolymers were obtained [17]. Under this optimized reaction condition, we synthesized a series of fluorine-containing methacrylic polymers (Scheme 3).

### 3.2. GPC, ^1^H-NMR, FT-IR, Elemental Analysis Results of Polymers

The ratio of fluorinated monomer and alkyl methacrylate was varied to study the effect of the amount of **5** in the copolymers on the yield of polymerization and properties of the products (*M*_n_, *M*_w_/*M*_n_, *T*_g_, *T*_d_, θ_water_, θ_Bioctyl_, mol % and F %); the results are shown in Table 1 (the key spectras and images of contact angles were presented in Figures S1–S75 and Tables S1–S35 in the Supplementary Materials). The experimental results demonstrated that, with the variation of molar ratio of fluorinated monomer and alkyl methacrylate ranged from 1:1 to 1:8 in the case of the same monomer, the degree of polymerization and *M*_w_/*M*_n_ of the product tend to increase commonly. The *M*_n_ of each copolymer was up to 10000, *M*_w_/*M*_n_ of most copolymers were near 2.00 and the yield of every sample was more than 59%. 

**Figure 1:**
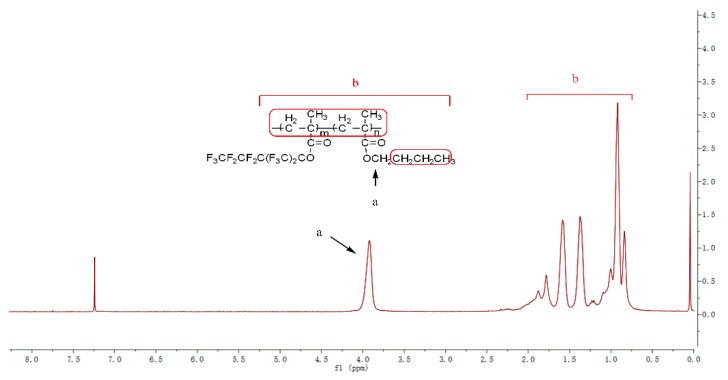
Proton NMR spectrum of poly(**5**-co-BMA) with feed molar ratio of 5 to BMA was 1:6.

The composition of poly(**5**-co-**7**) (the polymers was copolymers of **5** and **7**) was calculated from elemental analysis and ^1^H-NMR. Figure 1 shows the ^1^H NMR spectrum of poly(**5**-co-BMA). The proton signal laid at 3.92 ppm (Positon a) which was assigned to the –OCH_2_– group in BMA was easy to be identified from the NMR spectrum. The proton signals laid from 0.52 to 2.25 ppm (Positon b) represented the remaining CH_3_– and –CH_2_– groups in the copolymer. So, the molar ratio of the fluorinated monomer **5** in the polymer could be calculated as 0.204 according to the Fineman–Ross method [36]. Elemental analysis showed that the fluorine content of poly(**5**-co-BMA) with feed molar radio of **5** to **7** equal to 1:6 was 22.96% which was consistent with ^1^HNMR results (23.51%).

**Figure 2:**
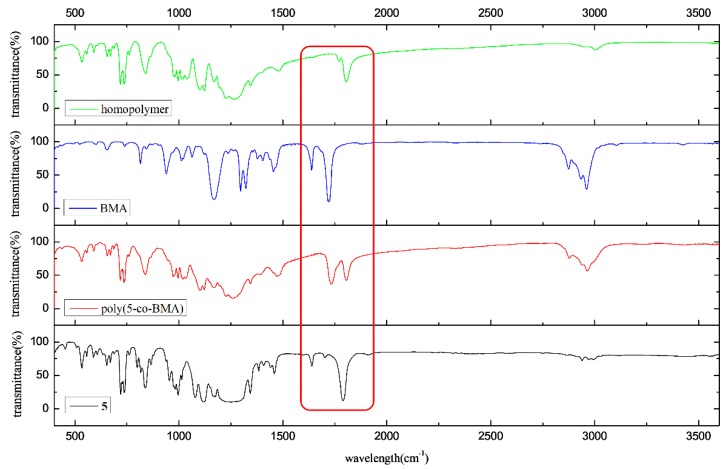
FT-IR spectrum of poly(**5**-co-BMA), homopolymer, butyl methacrylate and fluoromonomer **5**.

Figure 2 showed the FTIR spectrum of poly(**5**-co-BMA) with feed molar ratio of **5** to BMA was 1:1, homopolymer **6**, BMA and monomer **5**. In the curve of poly(**5**-co-BMA), the wide absorption band from 1805 cm^−1^ (–C=O of homopolymer) to 1730 cm^−1^ (–C=O of BMA) indicated lots of **5** and BMA were introduecd into coploymer molecule. Comparing the curves of homopolymer **6** and poly(**5**-co-BMA) with that of monomer **5** and BMA, the disappearance of the wide band from 1640 to 1620 cm^−1^ (–C=C– of BMA and **5**) indicated that BMA had reacted with **5** and self-polymerization of **5** had occurred at the site of the double bond. 

### 3.3. Thermal Properties Analysis

**Figure 3:**
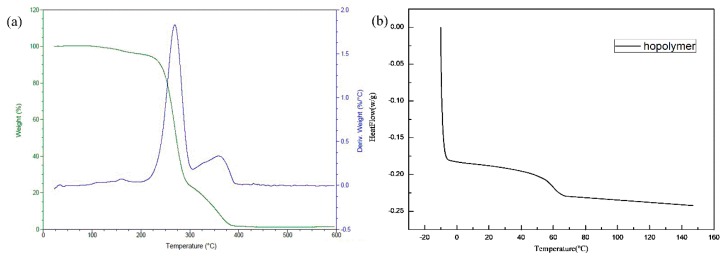
(**a**) TGA trace of homopolymer, (**b**) DSC curve of homopolymer.

TGA curves of homopolymer **6** and *T*_d_ of copolymers are shown in Figure 3 and Table 1. It could be observed that the initial degradation temperature of 6 was about 220 °C and the homopolymer degraded completely at 400 °C. With the increase of the ratio of alkyl methacrylate **7**, the decomposition temperature tends to increase. All copolymers had excellent thermal stability, degradation temperatures of copolymers with different molar ratio of **5** to **7** were all about 220–250 °C except those for poly(**5**-co-SMA), which indicated that the amount of **5** in the copolymer had less of an effect on the thermal stability of polymers. However, the degradation temperature of poly(**5**-co-SMA) was significantly higher than other polymers and was increased with the increasing of molar ratio of **5** to **7**. 

From Table 1, it was found that the value of *T*_g_ gradually approached that of homopolymer of **7** as the content of **5** decreased and *T*_g_ of the polymer tended to that of homopolymer **6** as the content of monomer **5** increased in case of the same monomers [37]. *T*_g_ tended to decrease as the length of the hydrocarbon chain increased when the monomers were different. The reason might be that the fluorine-containing side chain hindered the rotation and the motion of the molecular chain [38], which affected *T*_g_ of the copolymer. As shown in Table 1, *T*_g_ of the polymer fluctuated in the range of −51~103.8 °C. *T*_g_ can be adjusted by controlling the feed ratio of different monomers to adapt to the technical specifications of different applications.

### 3.4. Surface Properties Analysis

**Figure 4:**
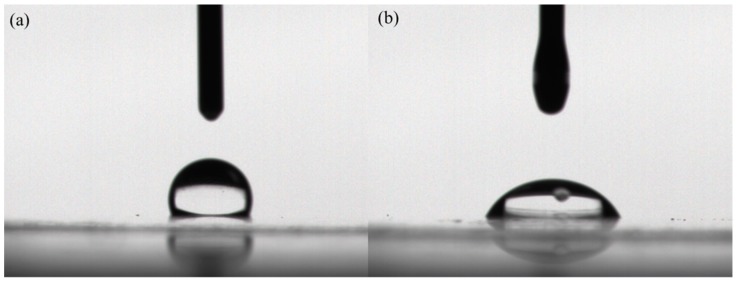
Contact angle of reagent on the surface of hopolymer film: (**a**) water; (**b**) n-hexadecane.

The film of homopolymer 6 had strong water and oil repellency (Figure 4). The contact angle of water on the film of homopolymer **6** (113.84°) were similar with that of ((CF_3_)_2_CF)(C_3_F_7_)CHOC(O)CH**_2_**=CH_2_ homopolymer (about 117°), but greater than that of C_6_F_13_(CF_3_)CHOC(O)CH=CH_2_ homopolymer (about 107°) at the same concentration. The contact angle of n-hexadecane on the homopolymer’s film (65.55°) was slightly smaller than that of C_6_F_13_(CF_3_)CHOC(O)CH=CH_2_ homopolymer (about 74°), but close to that of ((CF_3_)_2_CF)(C_3_F_7_)CHOC(O)CH_2_=CH_2_ homopolymer (about 68°) [8].

As can be seen from Table 1, the contact angles of water and n-hexadecane increased with the increase of fluorine content because the water and oil repellency of the polymer are caused by the fluorine-containing group. From Table 1, we knew that the contact angle decreased linearly with the decrease of **5** at the beginning, and then decreased at a subdued pace for most copolymers. The reason may be that the fluorinated polymethacrylate, as the side chain of the copolymer, moved to the surface easily during the formation of the film. When the content of fluoromonomer decreased, the movement of fluorine-containing segments might be restricted, thus the contact angle decreased at a linear rate [39]. Besides, the water repellency was significantly reduced when the fluorine content of the copolymer was less than 20% while the oil repellency was significantly reduced when the monomer molar ratio of **7**:**5** was more than 6. When *n* (R = C*_n_*H_2*n*+1_) ≥ 8, the hydrophobic properties of the copolymer can also be better than those of C_6_F_13_(CF_3_)CHOC(O)CH=CH_2_ homopolymer. We speculate that when the lengths of alkyl chains are 8 and 12, alkyl chains and fluorine chains can play a synergistic role for the water repellency of copolymers, since alkyl chains also have certain hydrophobicity. The same reason can be extended to the curve increases as the molar ratio of monomer **7** increases. However, the hydrophobicity decreases with the increase of the monomer **7** when the alkyl chain length is 18, which may be due to the long alkyl chain covering part of the fluorocarbon chain. At the same time, as the alkyl chain is lipophilic, the oil repellency decreases with the increase of the alkyl chain length [40]. 

The static wettability of a solid surface can be investigated and characterized through measuring the liquid equilibrium contact angle (CA). For a flat and chemically homogeneous surface, the CA (θ_e_) is only determined by the surface free energy of the solid substrate and given by the classical Young’s equation (Equation (1)):

(1) cos θ_e_ = (γ_sv_ − γ_sl_)/γ_lv_

where γ is the surface tension, and sv, sl, and lv are solid–air, solid–liquid, and liquid–air interfaces. For a rough surface where air pockets are trapped underneath the liquid, the CA (θ_c_) can be described by the equation from Cassie and Baxter (Equation (2)) [41]:

(2) cos θ_c_ = *f*_s_ cos θ_e_ + *f*_v_

where *f*_s_ is the liquid/solid contact area divided by the projected area, and *f*_v_ is the liquid/vapor contact area divided by the projected area. Equation (2) indicates that apparent CAs (θ_c_) depends on two factors: surface free energy (θ_e_) and surface geometrical structure (*f*_s_ and *f*_v_).

Surface free energy of poly(**5**-co-LMA = 1:15) and homopolymer **6** was determined and compared by measuring CAs (θ_e_) of their films for different test liquids (calculated by the method Wu). The corresponding CAs of poly(**5**-co-LMA = 1:15) and homopolymer **6** with water, and n-hexadecane as well as the surface free energy were shown in Table 1. Homopolymer **6** had a very low surface energy. The extremely low surface free energy of homopolymer **6** may originate from the unique structure of the polymer with the branched fluorocarbon chain, which endows the treated glass with excellent oil repellency [42]. In addition, the surface free energy of poly(**5**-co-LMA = 1:15) was apparently higher than that of homopolymer **6**. The water contact angles of poly(**5**-co-LMA = 1:15) kept in 113.23°, which indicated that the surface roughness was the major influencing factor (Equation (2)).

**Figure 5:**
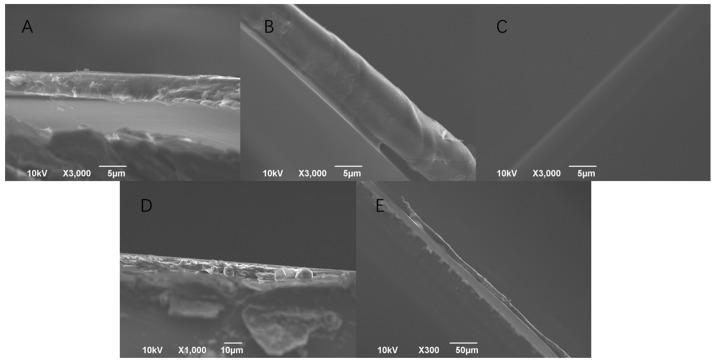
SEM images of (**A**,**D**) homopolymer **6**, (**B**,**E**) poly(**5**-co-LMA = 1:15) and (**C**) homopolymer of LMA coating transect on the glass substrate.

The surface profiler step tester and SEM were used in the analysis of the surface of poly(**5**-co-LMA = 1:15), homopolymer of LMA and homopolymer **6** films. The results of the step tester showed that there was a thin film with a thickness of about 350 nm on the surface of glass substrate, and the thicknesses of the three polymer films were similar. It can be seen from SEM that the homopolymer **6** film and homopolymer of LMA film were smooth (Figure 5A,C) and their molecular distribution was even (Figure 5C,D). The surface of poly(**5**-co-LMA = 1:15) film was out-of-flatness (Figure 5B) in the section and its surface roughness was large (Figure 5E). However, it can be seen that the thickness of the films was about 350 nm, which was consistent with the results of the step tester. It was further verified that the introduction of methacrylate caused the surface roughness of the film to increase.

**Figure 6:**
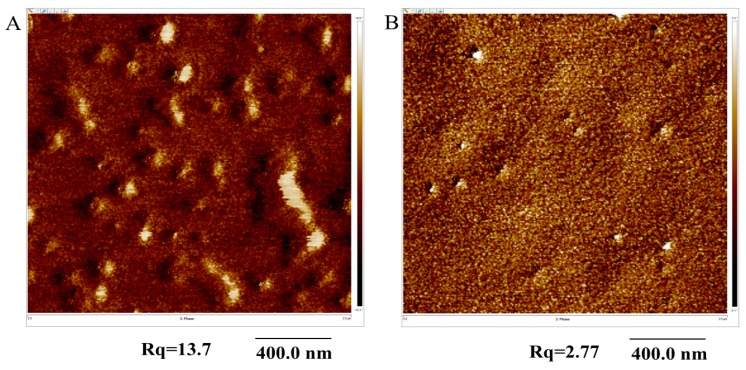
AFM images of (**A**) poly(**5**-co-LMA = 1:15) film and (**B**) homopolymer **6** film.

In order to further characterize the surface morphology of the material surface, Bruker Dimension FastScan was employed to measure the film surface roughness, and films were coated on glass slides as described above. *R*_q_ (root-mean-squared roughness) is the most common roughness parameter to give useful information about the sample surface, i.e., larger values mean greater topographical variation in the image. From the height image in Figure 6, we can see that as alkyl methacrylate was introduced, the roughness of the surface increased. The surface roughness played a significant role in surface properties, the increase of surface roughness led to a similar contact angle of water between poly(**5**-co-LMA = 1:15) and homopolymer **6**. So, the surface roughness of the membrane had a great influence on the hydrophobicity and oil thinning, and the surface roughness increased with the hydrophobicity. Besides, according to the relevant literature [43], the phase diagram showed that the brighter regions represent the harder materials. As you can see from the graph, the brightness area of Figure 6A was dense. The introduction of methacrylate increased the hardness of the films, thereby enhancing the wear resistance of the coating material.

**Figure 7:**
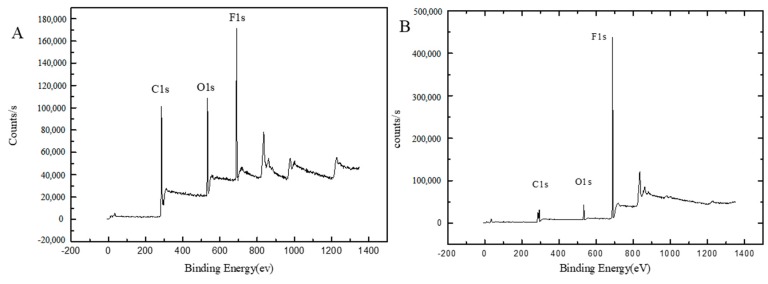
XPS spectra of (**A**) poly(**5**-CO-LMA = 1:15) film and (**B**) homopolymer **6** film.

XPS was utilized in this work to investigate the chemical composition of the polyacrylate films on glass substrates and results are shown in Figure 7. The signals at 688, 529, 283 eV were assigned to the F 1*s*, O 1*s* and C 1*s*, respectively [44,45]. The atomic percentages of F atoms of two different films measured from XPS. The poly(**5**-co-LMA = 1:15) contained 2.3% fluorine theoretically, while the experimental value was 16.55%, indicating that the organic fluorinated segment tended to migrate onto the surface and occupied it during the formation of the latex film, thus can effectively reduce the surface free energy of the film. But it was much lower than that of homopolymer **6** (56.86%), the introduction of methacrylate can improve the roughness, but reduced the content of fluorine-containing segments. When the fluorine content was lower than a certain value, the oil-thinning property decreased. Because the surface tension of n-hexadecane is very low, maintaining a higher contact angle on the solid surface requires a lower surface free energy, that is, a higher fluorine content.

In view of the above results, it is not difficult to realize water repellency because of the high surface tension of water (~72 mN/m) [46]. Hydrophobic surfaces achieved by the combination of surface roughening and lowering the surface free energy have been widely reported [47,48,49,50,51]. When the molar ratio of **5** to LMA is 1:15, the hydrophobicity of poly(**5**-co-LMA) can reach that of homopolymer 6 because of its high roughness. So, it can be used as a potential hydrophobic material. However, in order to achieve oil repellency, the surface free energy of a substrate lower than 20 mN/m is necessary because typical surface tensions of oils are 20–30 mN/m [42,51,52,53,54]. The high fluorine content of homopolymer 6 gives it low surface free energy, and it is a potential oil displacement material.

## 4. Conclusions

A novel fluorine-containing methacrylate monomer **5** was successfully prepared by two steps from perfluoro-2-methyl-2-pentene. All the reactions were easy and of mild conditions, low cost and high yields, which could be promisingly applied to the industrial production. Homopolymer **6** was obtained in high yields without solvent; **6** had excellent repellent properties which were similar with polymers containing ((CF_3_)_2_CF)(C_3_F_7_)CHOC(O)CH_2_=CH_2_, but superior to polymers containing primarily straight-chain perfluorohexyl (C_6_F_13_–) moieties. Nevertheless, since **6** was insoluble in conventional solvents, its application was limited.

In this paper, methacrylates with different alkyl chain length were copolymerized with **5** to achieve a series of copolymers. The structure of copolymer was confirmed by GPC, elemental analysis and ^1^H-NMR and FT-IR. The *M*n of each copolymer was up to 10,000, and *M*_w_/*M*_n_ of the copolymers were near 2.00 when the radio of **7** to **5** was less than 6. The contact angle and *T*_g_ could be adjusted by controlling the monomer feed ratio to meet the technical requirements of different practical applications. It was found that as the content of monomer **5** increased, *T*_g_ of the polymer tended to that of homopolymer **6**. By changing the ratio of feeding, *T*_g_ fluctuated within the range of −51~103.8 °C. All the polymers had stable thermal decomposition performance. The contact angle of water and n-hexadecane increased with the increase of fluorine content. Water repellency remarkably decreased when the fluorine content of the copolymer was less than 20%, while oil repellency remarkably decreases when the monomer molar ratio of **7** to **5** was more than 6. 

In summary, the homopolymer and copolymers we produced can be used as low surface energy materials to impart repellency and thermal properties of various substrate films. Poly(**5**-co-LMA = 1:15) and homopolymer **6** can be used as potential water repellent and oil repellent materials. By introducing other functional monomers, this kind of copolymers containing the –C(CF_3_)_2_CF_2_CF_2_CF_3_ group can be synthesized for specific use.

## Figures and Tables

**Table 1:** Characteristics of the polymers ^a^.

Polymer	5:7 ^b^	Yield ^c^ (%)	*M*n ^d^ (×10^4^)	*M*_w_/*M*_n_^d^	*T*_g_^e^ (°C)	*T*_d_^f^ (°C)	θ_water_^g^ (°)	θ_Bioctyl_^g^ (°)	5:7 ^h^ (%)	F ^i^ (%)
**6**	0	79	–	–	59.47	248.27	113.84	65.55	–	58.07
Poly(**5**-co-BMA)	1:1	59	1.07	1.22	52.76	238.97	102.37	46.23	80.4	42.92
	1:2	63	1.41	1.43	51.27	236.73	100.03	44.52	27.6	27.73
	1:6	87	2.85	5.81	46.21	245.21	99.74	43.27	22.0	22.96
	1:8	76	2.16	1.83	42.82	243.73	97.41	42.68	14.4	17.40
	1:15	71	4.50	4.12	41.64	234.02	95.32	40.47	6.8	9.41
Poly(**5**-co-MMA)	1:1	66	1.02	1.55	49.40	232.34	85.06	52.70	87.8	–
	1:2	74	1.02	1.31	56.02	253.24	60.74	48.62	60.8	–
	1:6	81	1.56	1.43	68.65	249.97	32.49	45.37	36.2	–
	1:8	81	1.87	1.60	84.80	244.18	30.57	42.35	29.6	–
	1:15	68	1.67	1.62	103.80	241.27	18.58	36.53	15.2	–
Poly(**5**-co-EMA)	1:1	59	1.36	1.25	58.13	240.72	106.83	54.90	83.6	–
	1:2	77	1.45	1.74	53.71	241.09	101.64	50.53	30.8	–
	1:6	67	2.14	1.69	53.34	236.24	99.93	45.03	22.0	–
	1:8	59	3.13	1.91	52.15	241.08	95.64	42.10	10.4	–
	1:15	57.9	3.70	1.951	53.10	249.92	97.86	39.64	8.2	–
Poly(**5**-co-HMA)	1:1	68	1.90	1.66	11.28	229.64	103.37	51.78	74.8	–
	1:2	69	1.27	1.74	12.12	231.20	96.46	49.13	27.6	–
	1:6	79	2.72	8.83	1.76	239.61	97.39	36.96	13.6	–
	1:8	78	2.71	5.11	−0.61	242.75	95.87	35.05	8.8	–
	1:15	88	2.05	3.13	−5.06	246.26	98.12	34.75	4.0	–
Poly(**5**-co-OMA)	1:1	58	1.04	1.44	3.43	234.90	97.20	41.81	74.8	–
	1:2	64	1.37	2.07	5.12	217.04	103.28	30.31	21.1	–
	1:6	67	2.49	4.13	6.94	242.10	109.28	27.04	12.8	–
	1:8	61	1.94	2.92	6.89	240.13	109.47	22.20	8.0	–
	1:15	94	1.81	2.12	9.58	248.13	109.20	18.26	3.2	–
Poly(**5**-co-LMA)	1:1	59	1.10	1.53	−1.60	220.47	102.59	37.66	49.6	–
	1:2	65	1.57	1.70	−25.08	234.45	99.10	33.51	20.8	–
	1:6	67	2.24	2.20	−48.8	234.39	95.09	26.48	14.8	–
	1:8	67	2.26	2.15	−51.17	234.12	92.02	26.04	9.6	–
	1:15	88	2.31	2.24	−44.76	248.65	113.23	19.51	2.4	–
Poly(**5**-co-SMA)	1:1	67	1.63	1.81	14.36	283.71	107.18	37.06	34.4	–
	1:2	74	2.23	2.37	25.61	283.35	106.99	36.39	19.6	–
	1:6	91	5.13	5.51	29.76	344.39	104.42	30.64	10.0	–
	1:8	97	4.13	3.78	30.69	349.30	99.68	19.34	5.2	–
	1:15	80	2.45	2.41	33.48	354.89	95.98	5.26	0.8	–

^a^: Reaction conditions: 20% solution in toluene, 0.03 equiv. of AIBN, 80 °C in Ar; ^b^: Feed molar ratio of **5** to **7**; ^c^: Isolated yield; ^d^: The number-average molecular weight (*M*n) and the polydispersity (*M*_w_/*M*_n_) were determined by GPC; ^e^: *T*_g_ As determined by DSC; ^f^: *T*_d_ As determined by TGA; ^g^: Contact angles of water and n-hexadecane on polymer film (θ_water_ and θ_Bioctyl_); ^h^: Molar ratios of **5** and **7** in the copolymer was determined by ^1^H NMR spectroscopy; ^i^: As determined by elemental analysis.

## Supplementary Materials

The following are available online at http://www.mdpi.com/1996-1944/11/11/2258/s1, ^1^H NMR, ^19^F NMR, ^13^C NMR for the novel compounds and GPC, contact angle results of all the polymers were provided.
