# Supplementary material for: Preparation and Surface Properties Study of Novel Fluorine-Containing Methacrylate Polymers for Coating
Source: Materials (Basel). 2018 Nov 13;11(11):2258. doi: 10.3390/ma11112258 (PMC6266613; doi:10.3390/ma11112258)
Supplement: Supplementary file 1 [file materials-11-02258-s001.pdf]

materials

Supporting Information

Copies of ^{1}H NMR, ^{19}F NMR, ^{13}C NMR and GPC

1. Compound 4: Perfluoro-2-methyl-2-pentanol: 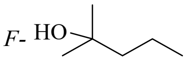

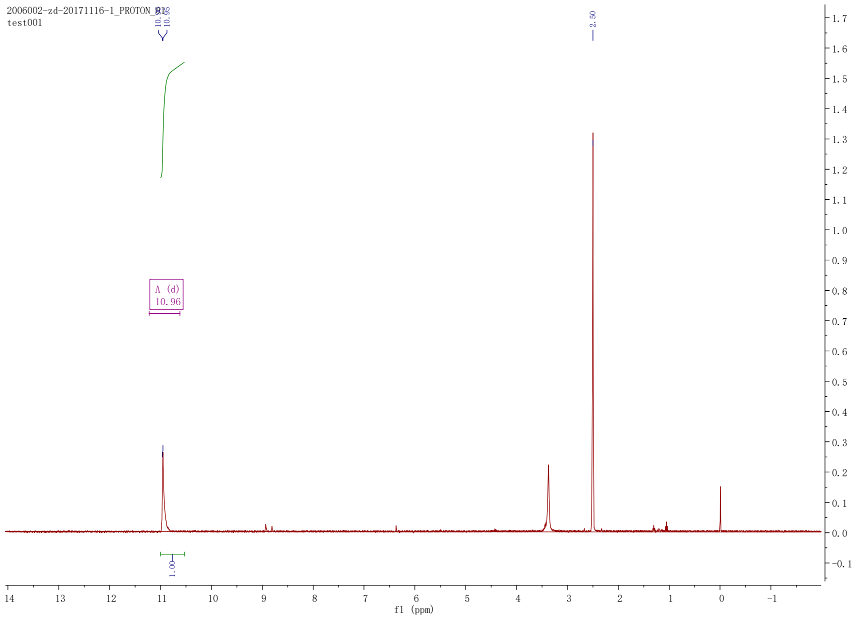

Figure S1. ^{1}H NMR (DMSO-d_6, 400 MHz).

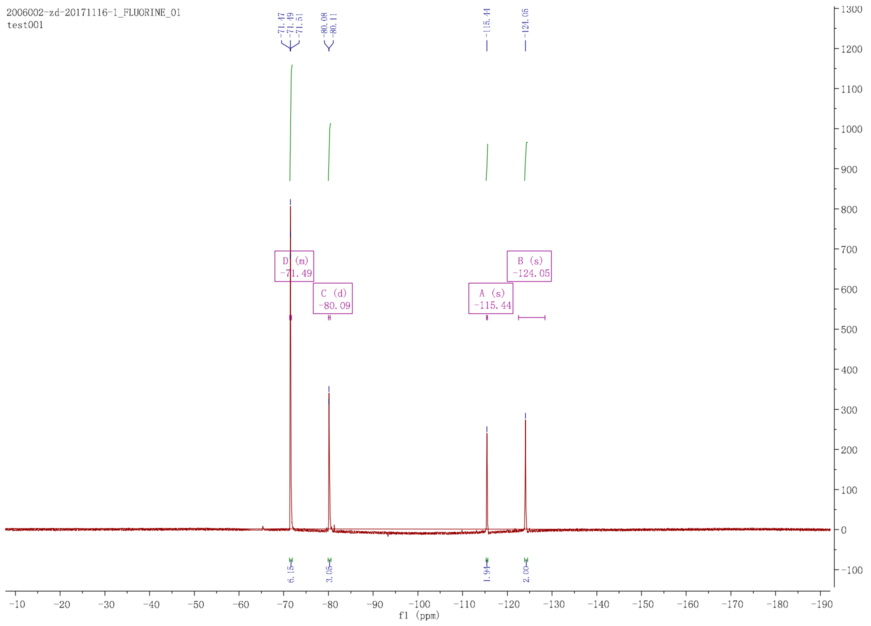

Figure S2. ^{19}F NMR (DMSO-d_6, 376 MHz).

2. Compound 5

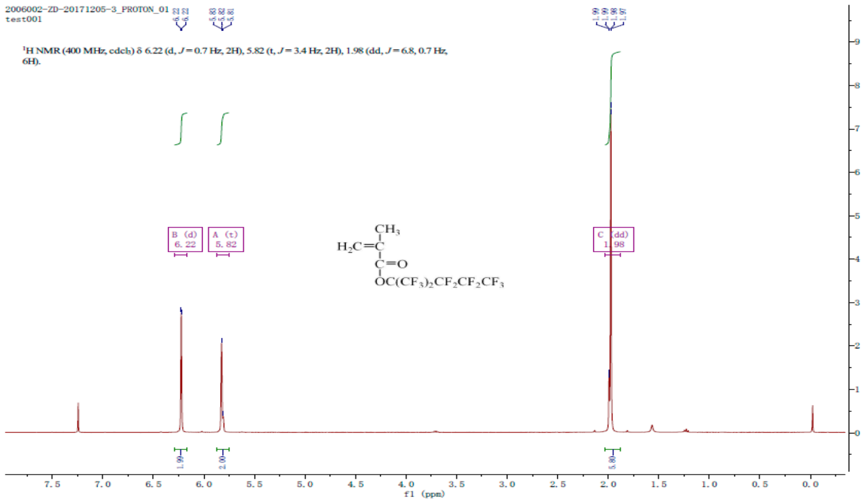

Figure S3. ^{1}H NMR ($CDCl_3$, 400 MHz).

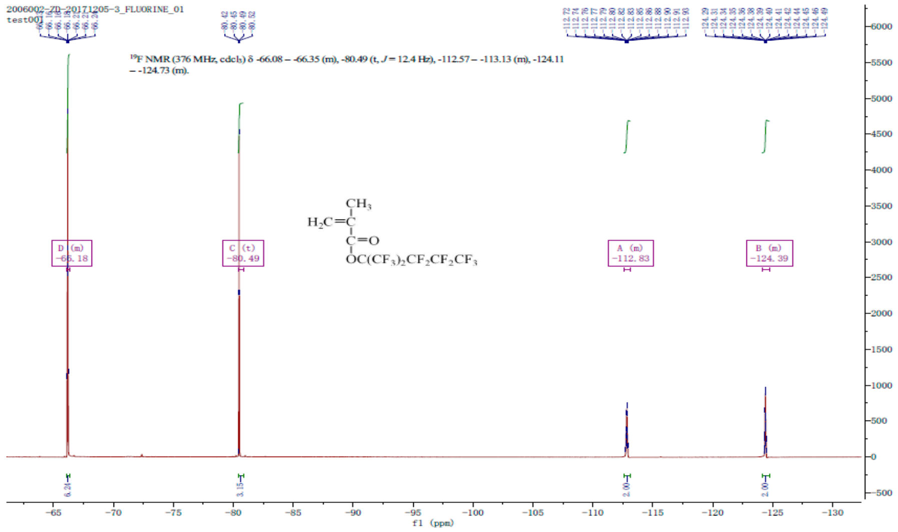

Figure S4. ^{19}F NMR ($CDCl_3$, 376 MHz).

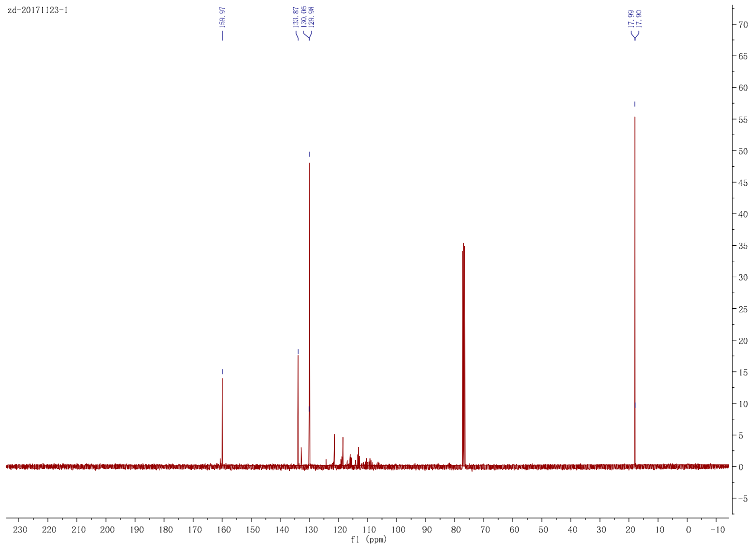

Figure S5. ^{13}C NMR (101 MHz, $CDCl_3$).

3. Compound of Poly (5-co-BMA) (1:1)

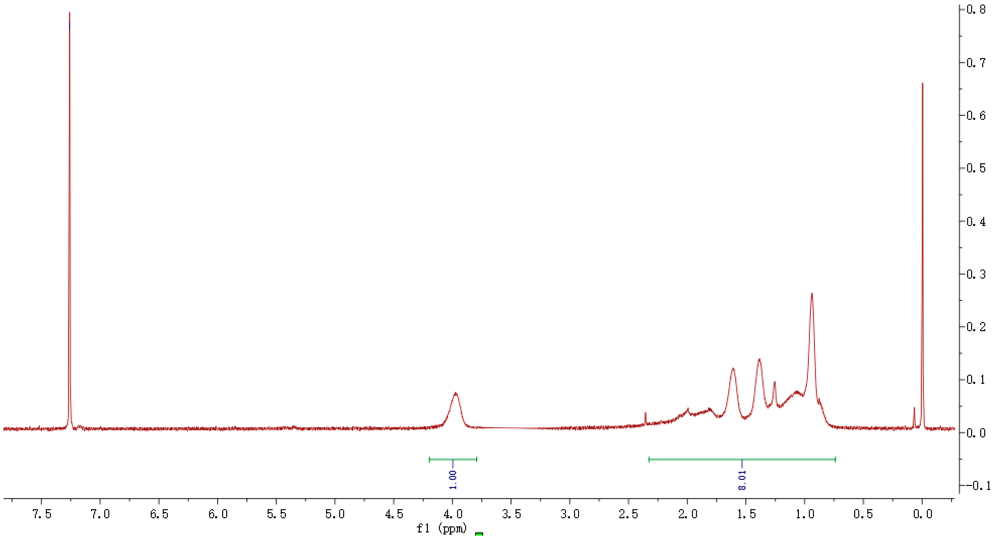

Figure S6. ^{1}H NMR ($CDCl_3$, 400 MHz).

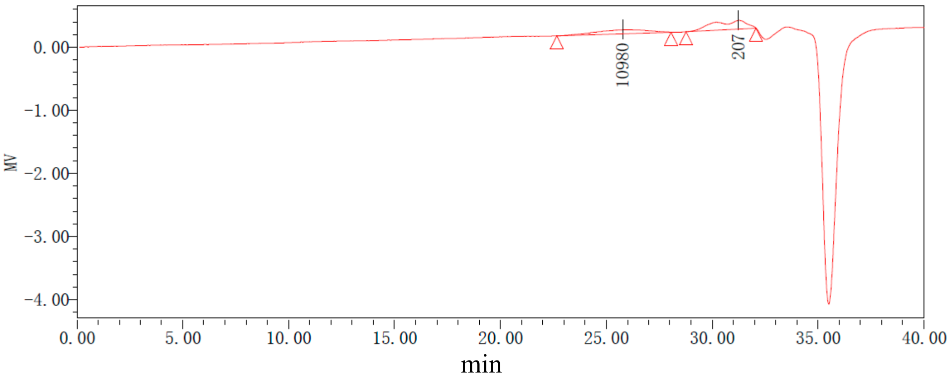

Table S1. GPC results.

M_n (D)	M_w (D)	MP	M_z (D)	$M_z + 1$ (D)	PDI
10686	13028	16043	10980	19471	1.219122

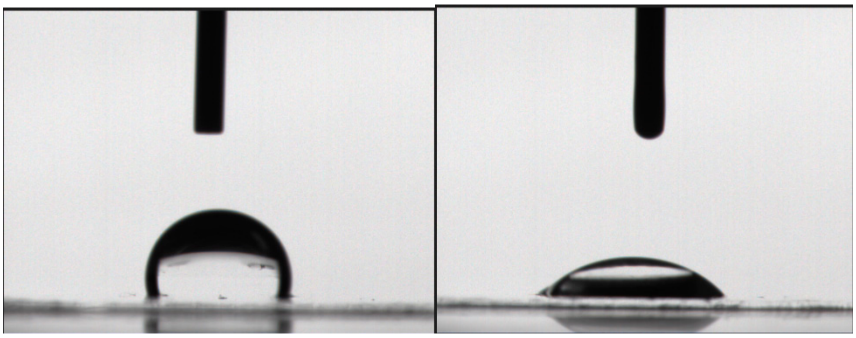

Figure S7. Contact Angle with Water and n-Hexadecane.

4. Compound of Poly (5-co-BMA) (1:2)

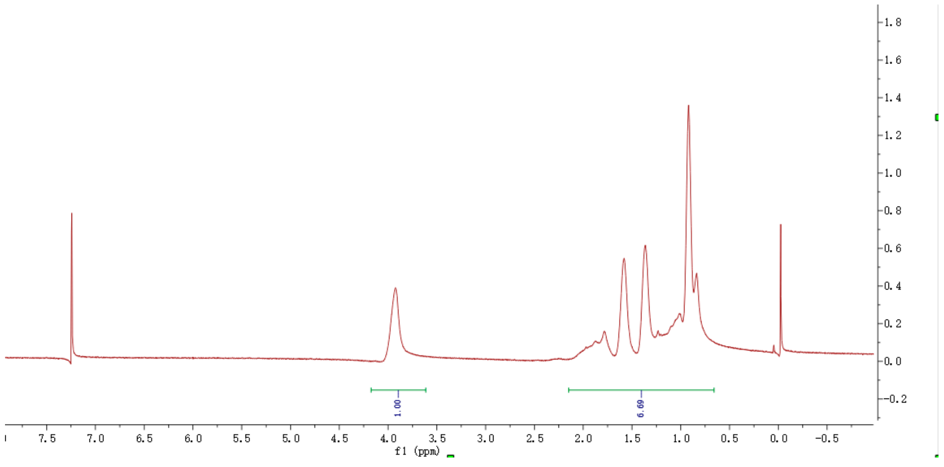

Figure S8. ^1H NMR ($CDCl_3$, 400 MHz).

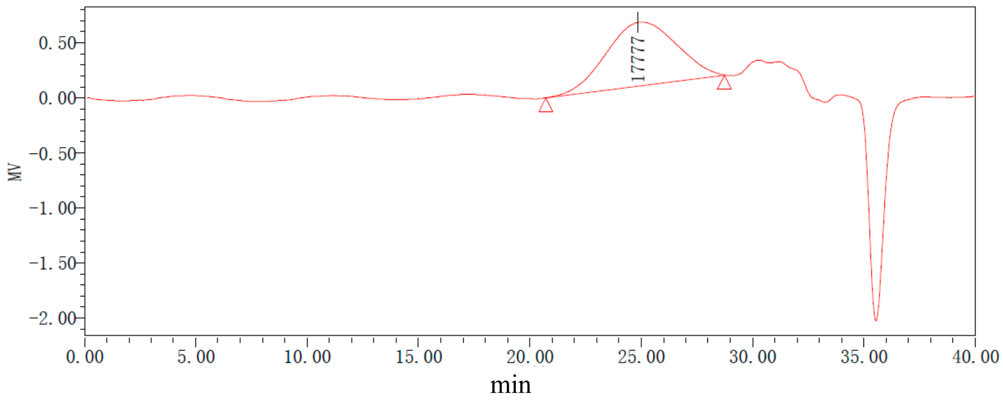

Table S2. GPC results.

M_n (D)	M_w (D)	MP	M_z (D)	$M_z + 1$ (D)	PDI
14094	20170	17777	28824	39443	1.431164

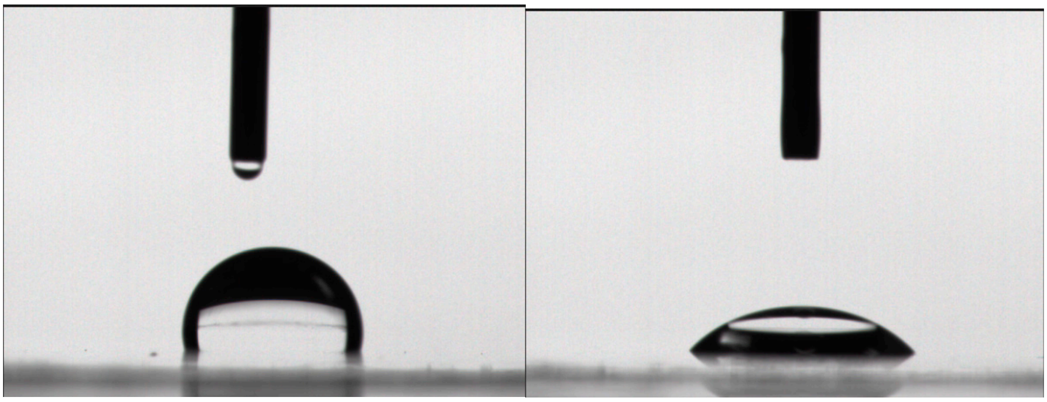

Figure S9. Contact Angle with Water and n-Hexadecane.

5. Compound of Poly (5-co-BMA) (1:6)

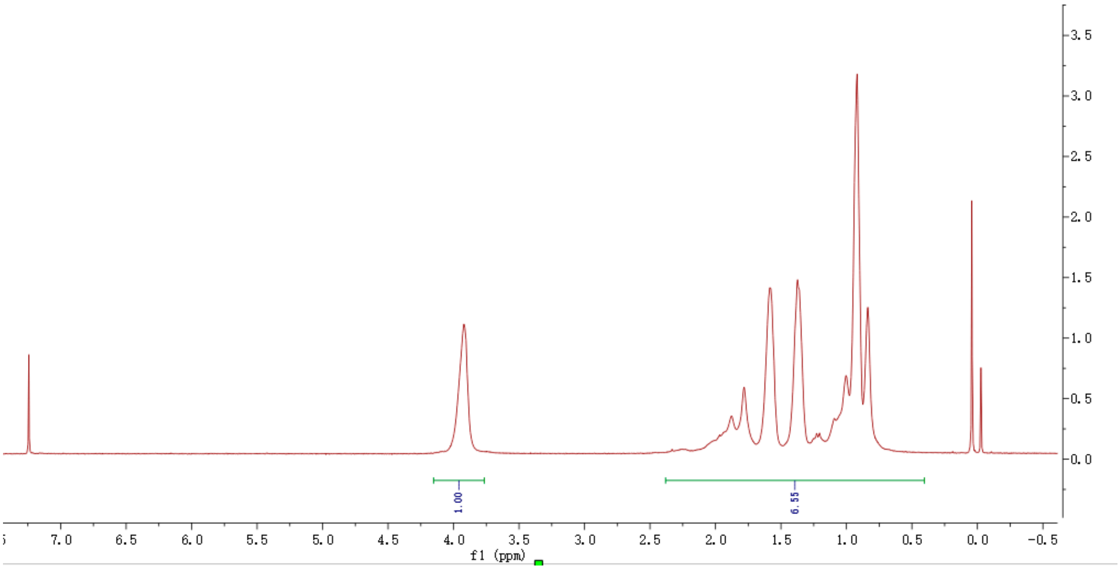

Figure S10. ^{1}H NMR ($CDCl_3$, 400 MHz).

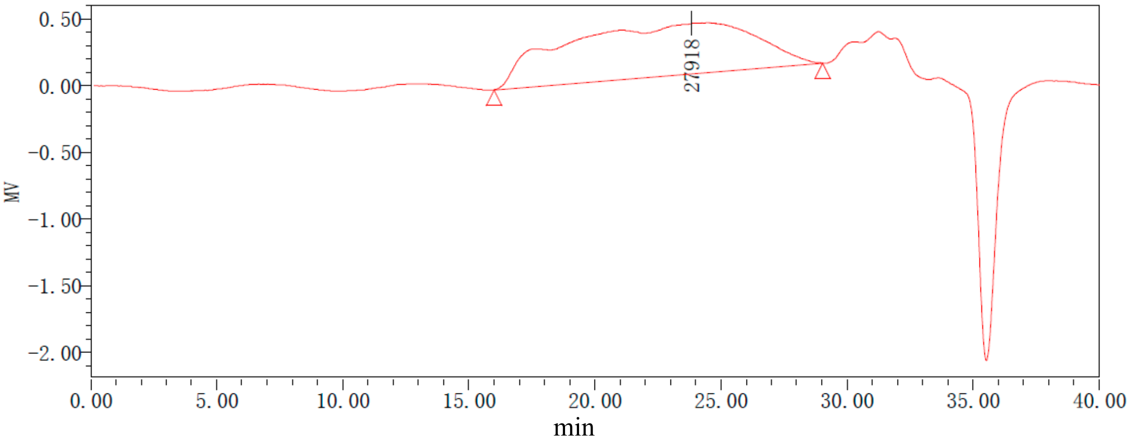

Table S3. GPC results.

M_n (D)	M_w (D)	MP	M_z (D)	M_z + 1 (D)	PDI
28459	165261	27918	538068	861607	5.806930

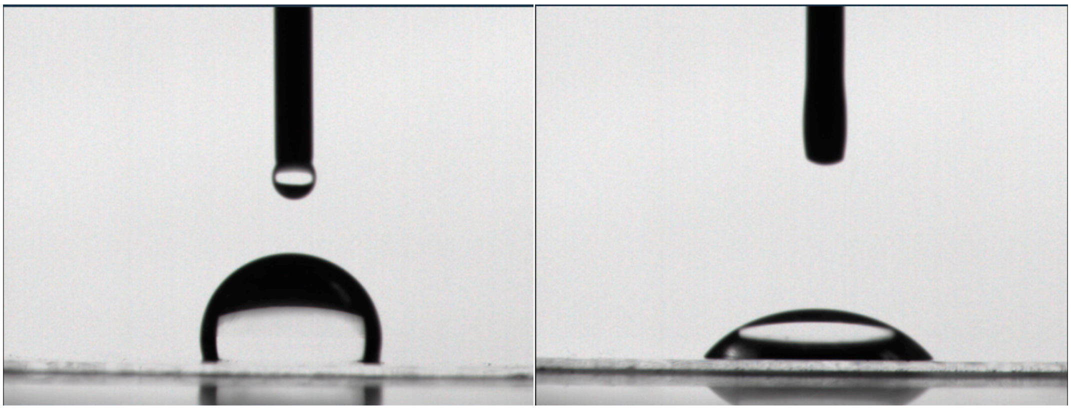

Figure S11. Contact Angle with Water and n-Hexadecane.

6. Compound of Poly (5-co-BMA) (1:8)

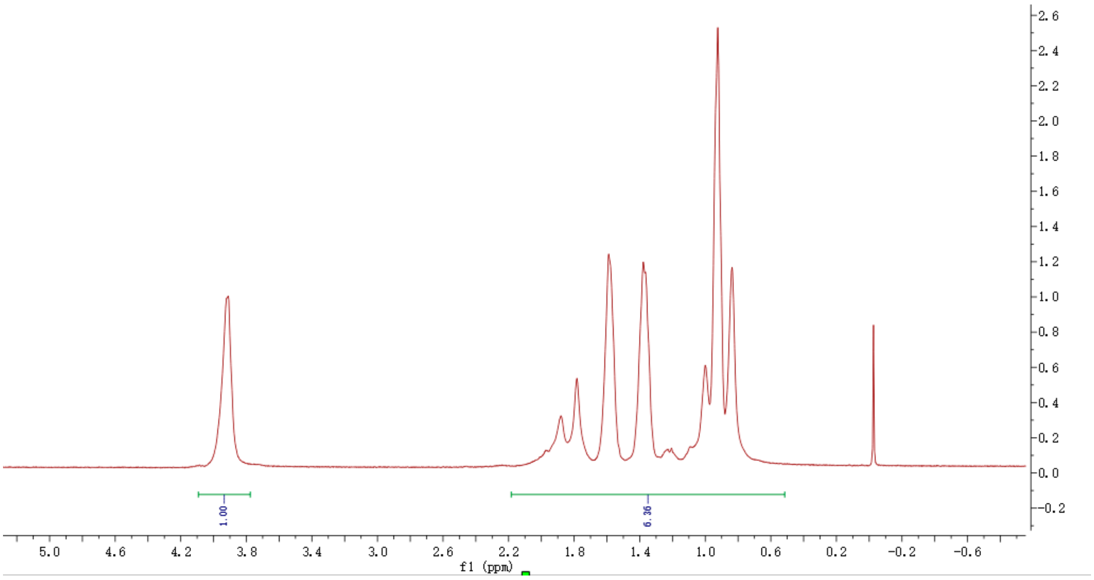

Figure S12. ^{1}H NMR ($CDCl_3$, 400 MHz).

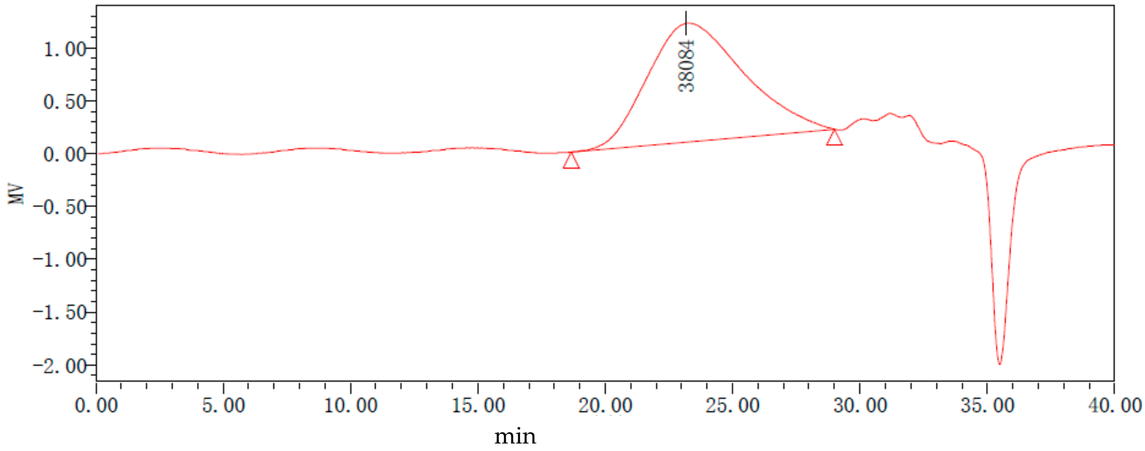

Table S4. GPC results.

M_n (D)	M_w (D)	MP	M_z (D)	$M_z + 1$ (D)	PDI
21609	39510	38084	63225	88432	1.828393

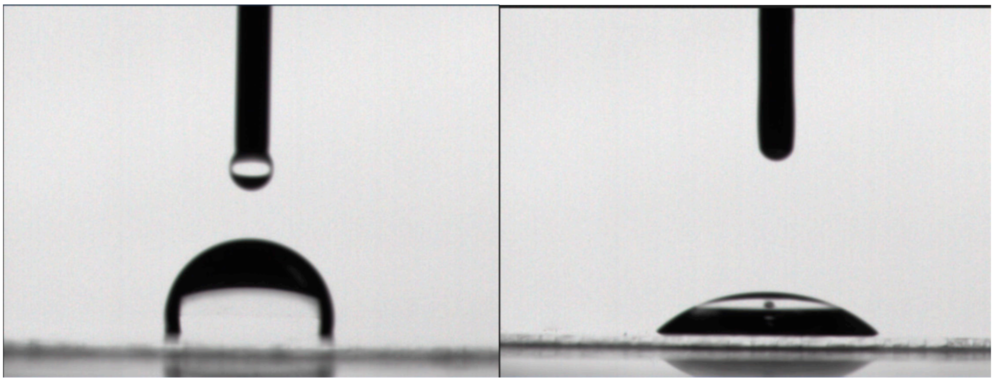

Figure S13. Contact Angle with Water and n-Hexadecane.

7. Compound of Poly (5-co-BMA) (1:15)

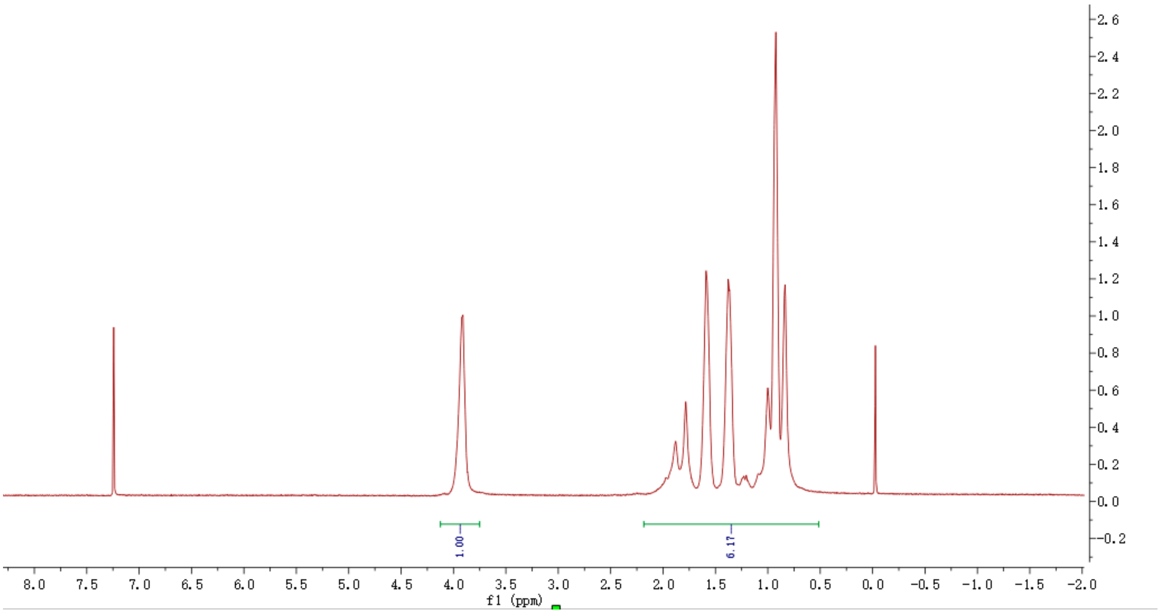

Figure S14. ${}^{1}H$ NMR ($CDCl_3$, 400 MHz).

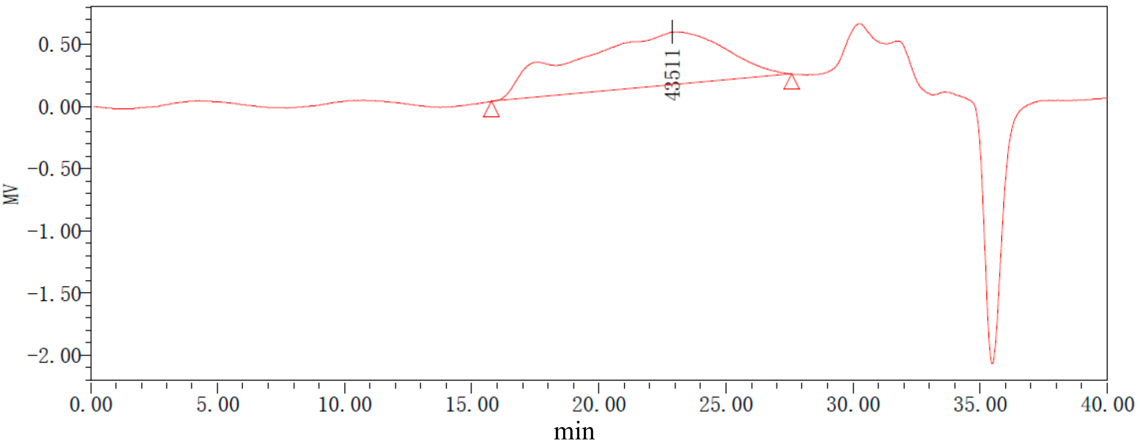

Table S5. GPC results.

M_n (D)	M_w (D)	MP	M_z (D)	M_z +1 (D)	PDI
44983	185349	43511	558085	897990	4.120455

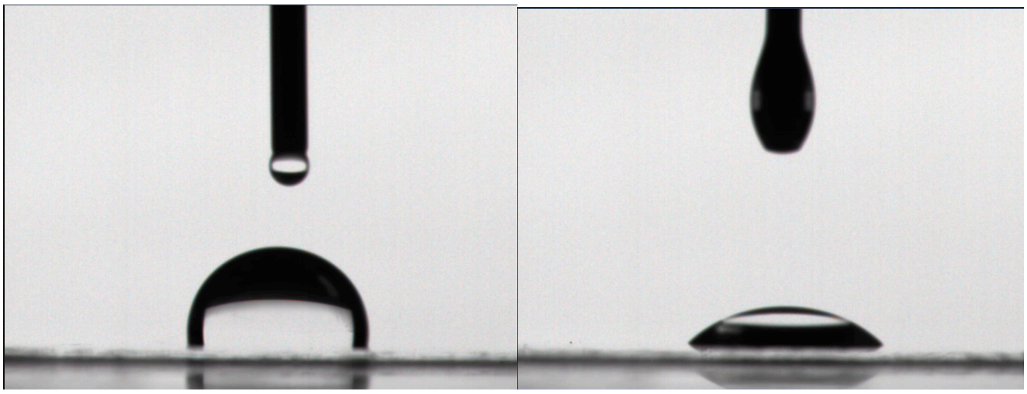

Figure S15. Contact Angle with Water and n-Hexadecane.

8. Compound of Poly (5-co-MMA) (1:1)

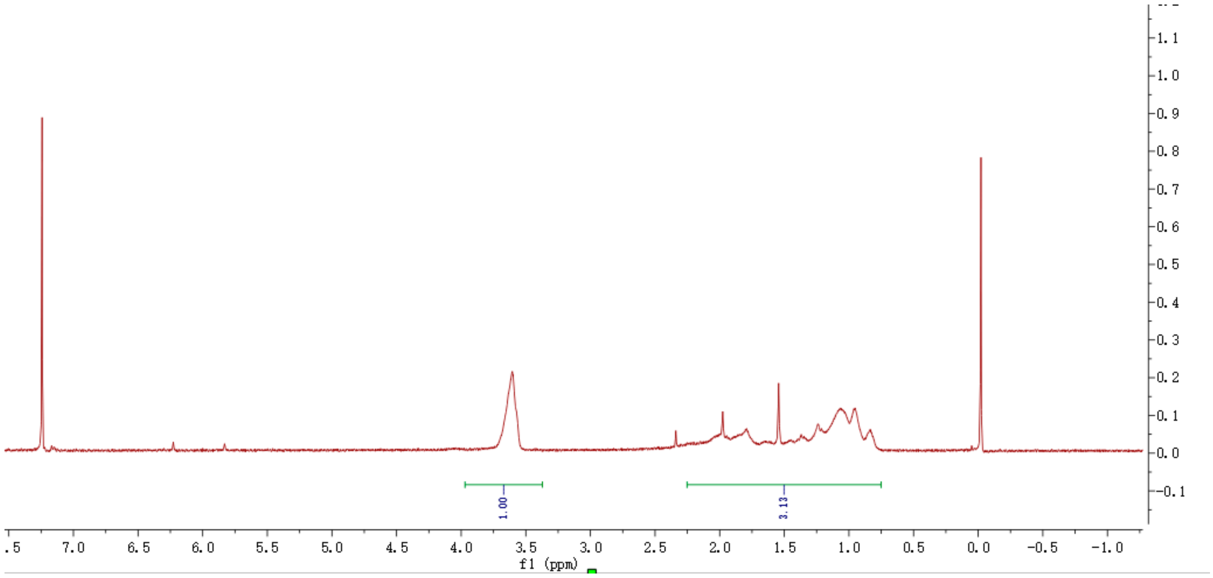

Figure S16. ^1H NMR ($CDCl_3$, 400 MHz).

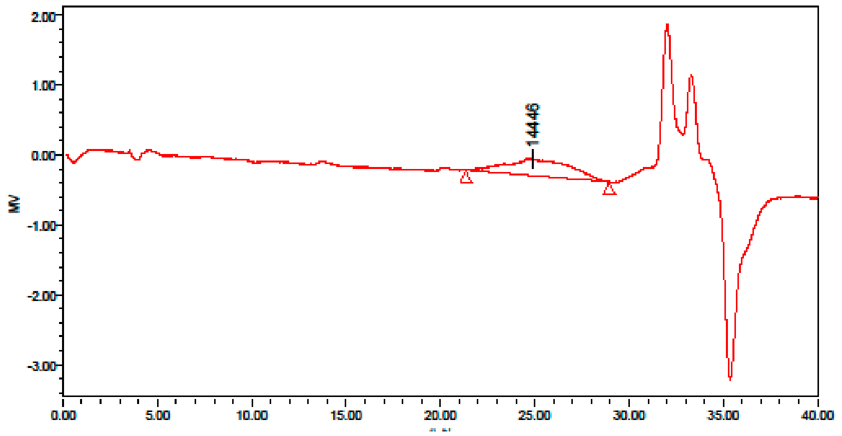

Table S6. GPC results.

M_n (D)	M_w (D)	MP	M_z (D)	$M_z + 1$ (D)	PDI
10195	15762	14446	24792	35193	1.546086

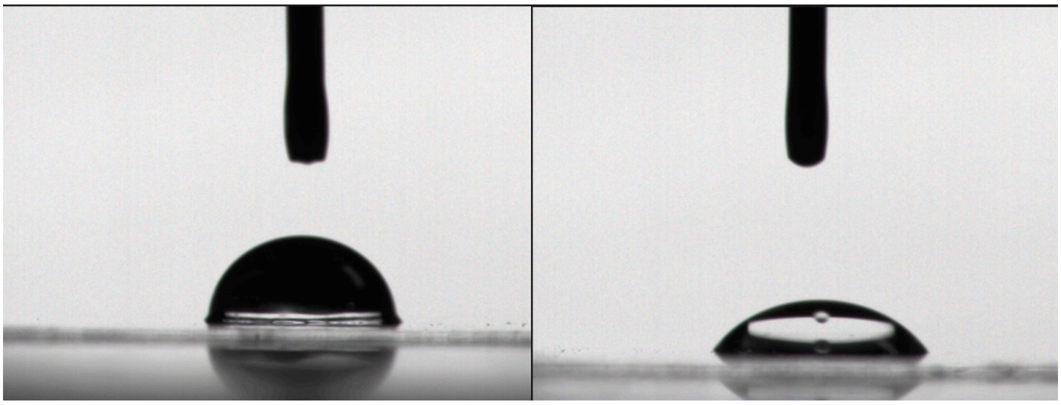

Figure S17. Contact Angle with Water and n-Hexadecane.

9. Compound of Poly (5-co-MMA) (1:2)

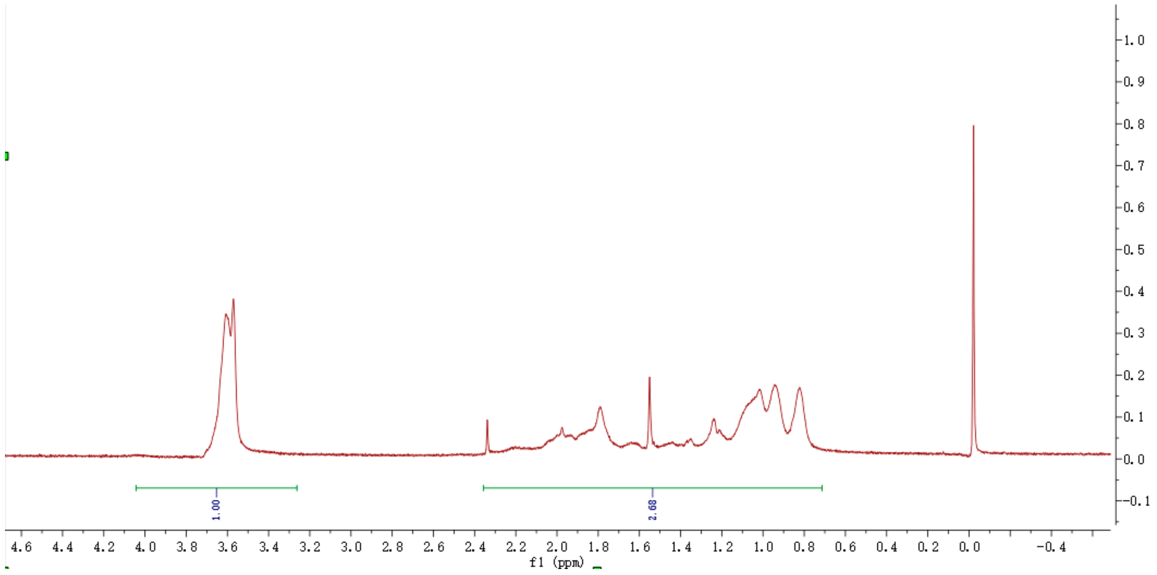

Figure S18. ${}^{1}H$ NMR ($CDCl_3$, 400 MHz).

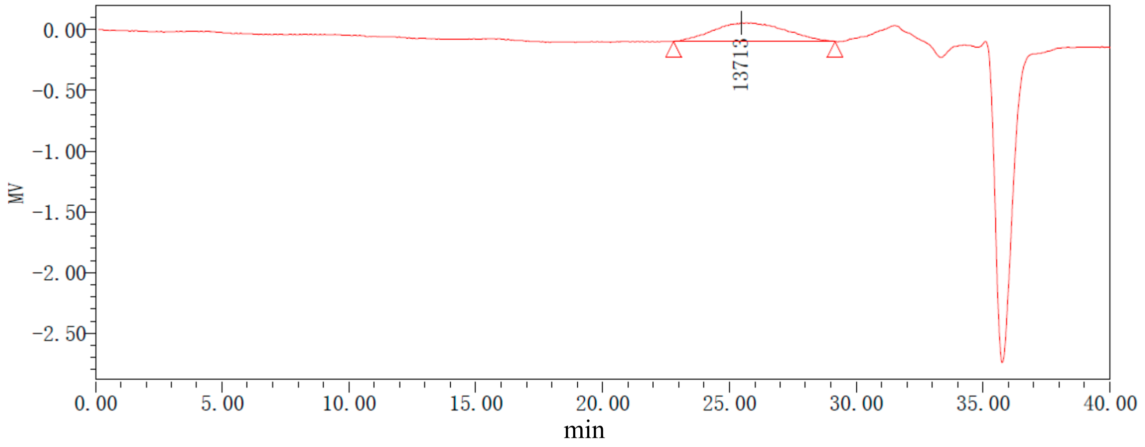

Table S7. GPC results.

M_n (D)	M_w (D)	MP	M_z (D)	$M_z + 1$ (D)	PDI
10210	13359	13713	17094	20969	1.308438

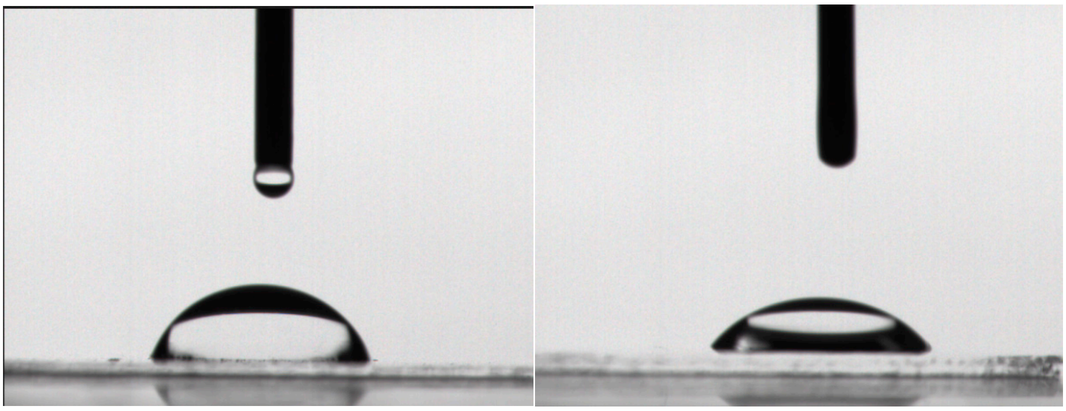

Figure S19. Contact Angle with Water and n-Hexadecane.

10. Compound of Poly(5-co-MMA) (1:6)

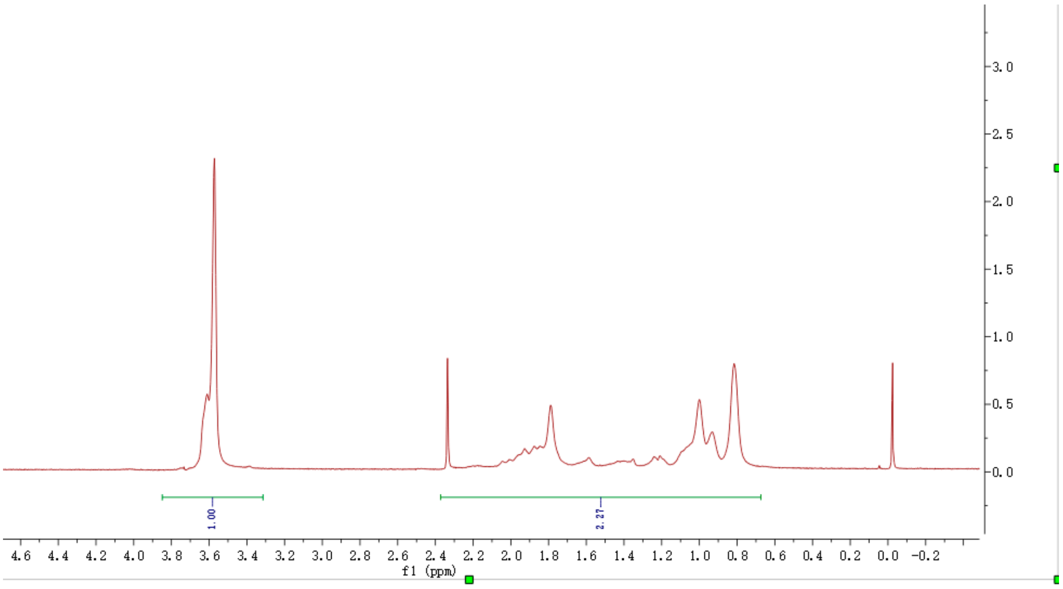

Figure S20. ^1H NMR ($CDCl_3$, 400 MHz).

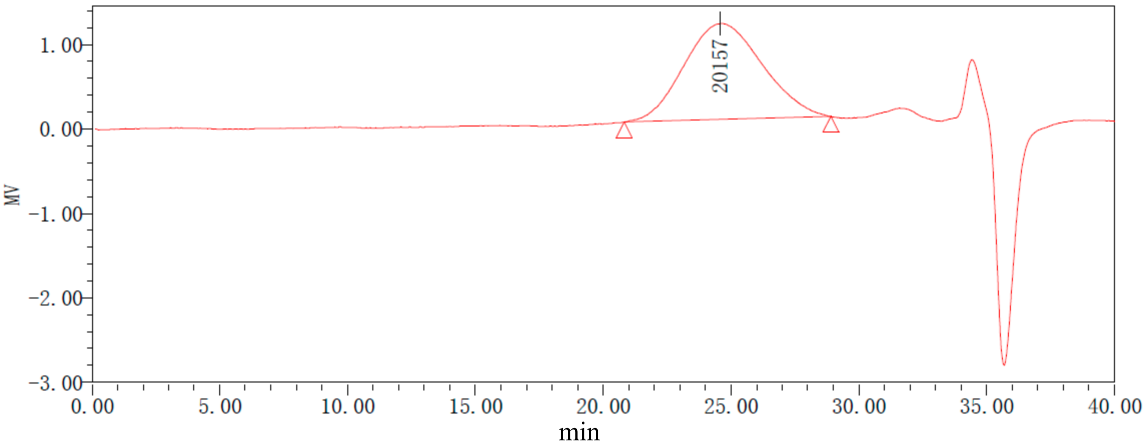

Table S8. GPC results.

M_n (D)	M_w (D)	MP	M_z (D)	$M_z + 1$ (D)	PDI
15593	22327	20157	31089	40976	1.431868

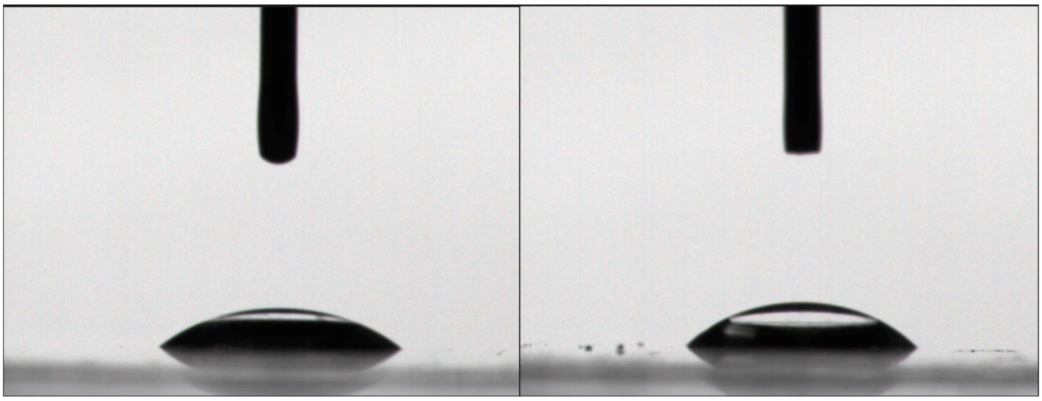

Figure S21. Contact Angle with Water and n-Hexadecane.

11. Compound of Poly(5-co-MMA) (1:8)

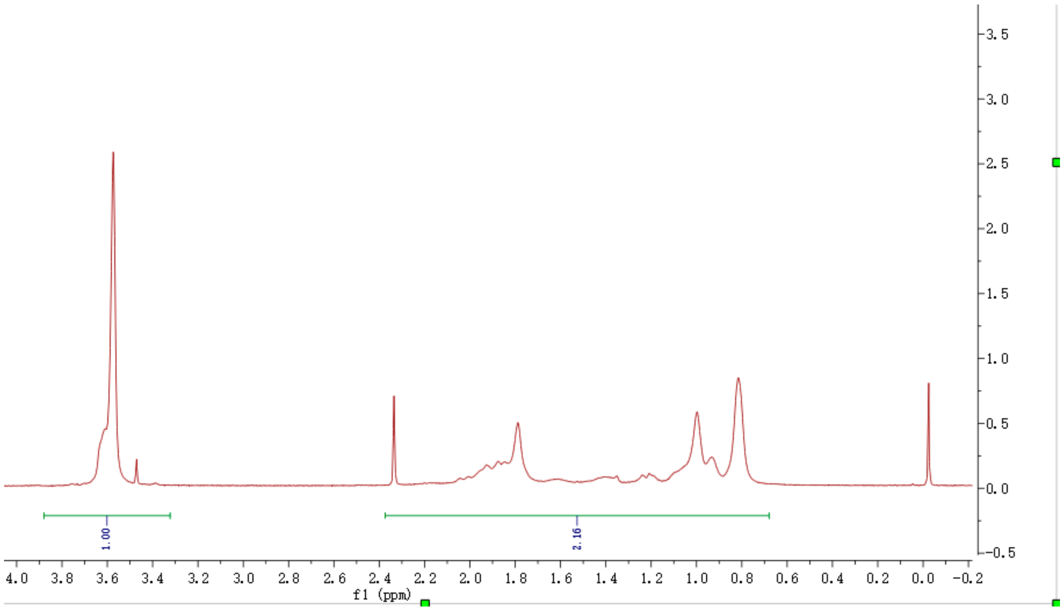

Figure S22. ^{1}H NMR ($CDCl_3$, 400 MHz).

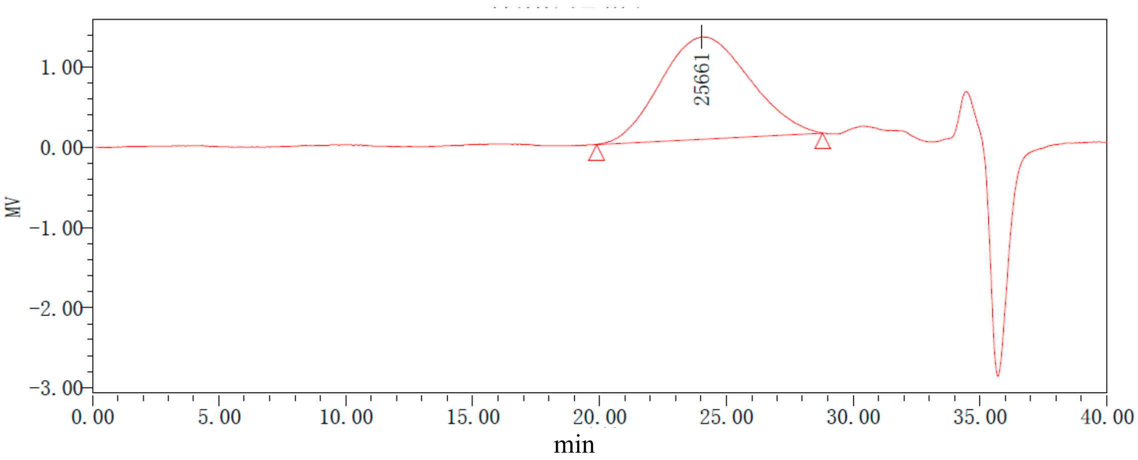

Table S9. GPC results.

M_n (D)	M_w (D)	MP	M_z (D)	$M_z + 1$ (D)	PDI
18710	29939	25661	45514	62849	1.600163

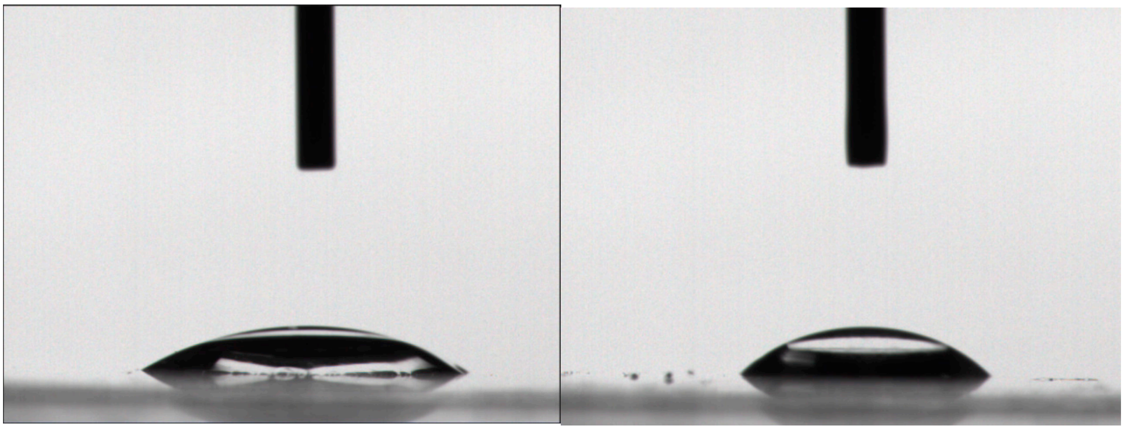

Figure S23. Contact Angle with Water and n-Hexadecane.

12. Compound of Poly(5-co-MMA) (1:15)

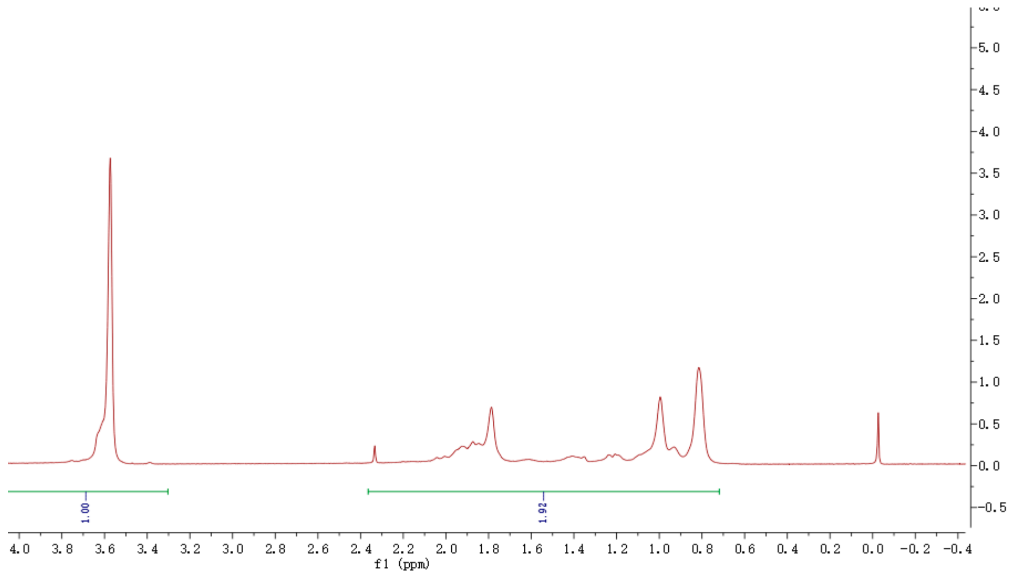

Figure S24. ^1H NMR ($CDCl_3$, 400 MHz).

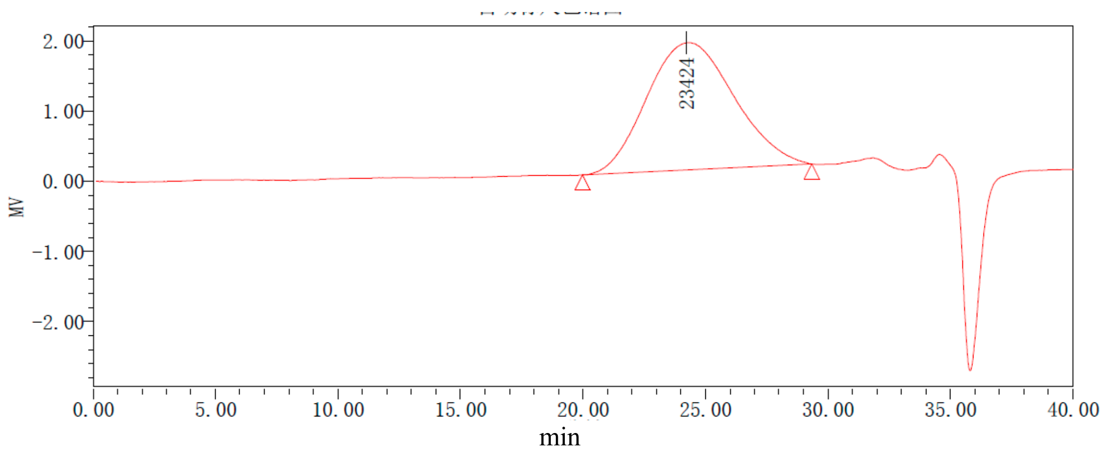

Table S10. GPC results.

M_n (D)	M_w (D)	MP	M_z (D)	$M_z + 1$ (D)	PDI
16657	27020	23424	41165	56811	1.622089

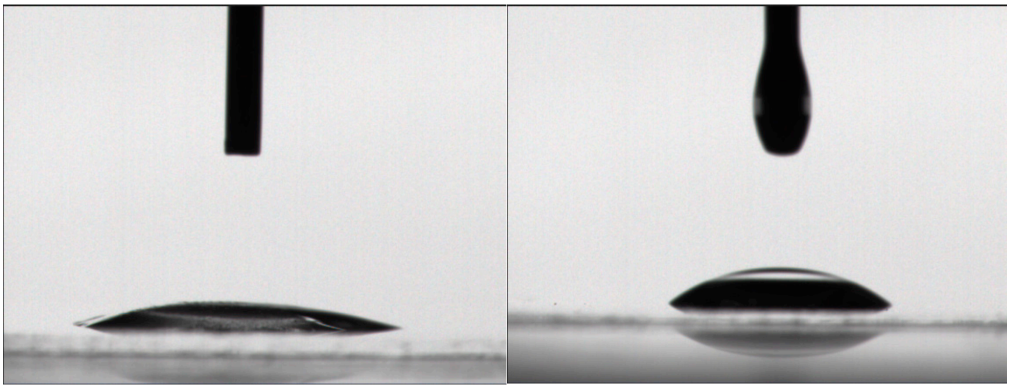

Figure S25. Contact Angle with Water and n-Hexadecane.

13. Compound of Poly(5-co-EMA) (1:15)

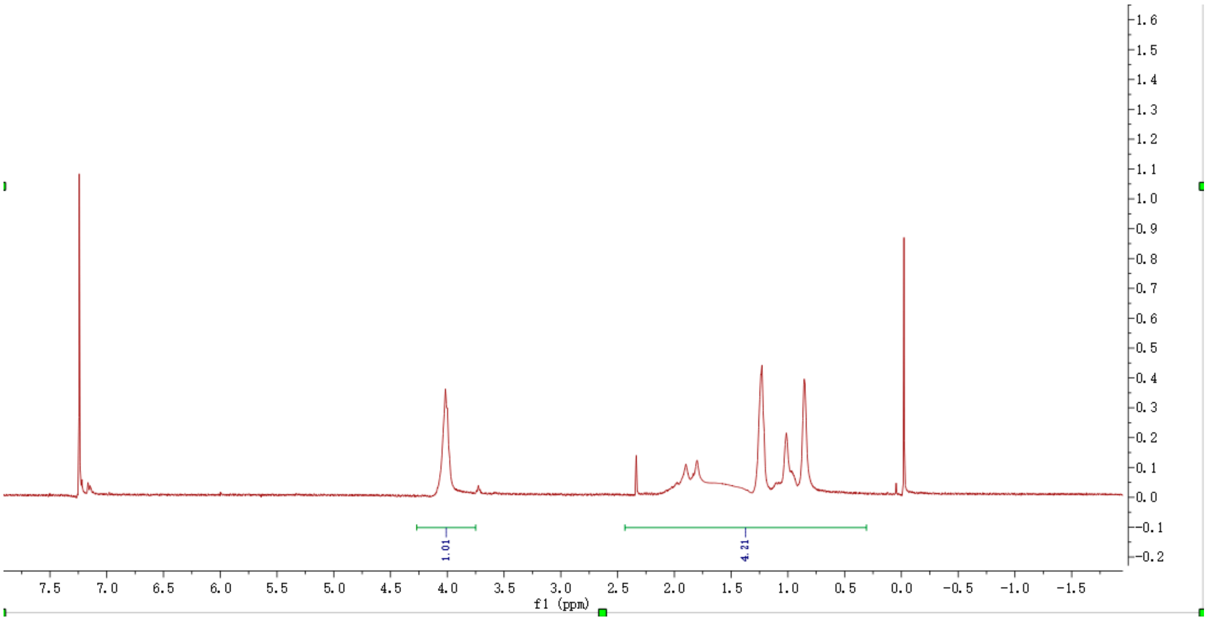

Figure S26. ^1H NMR ($CDCl_3$, 400 MHz).

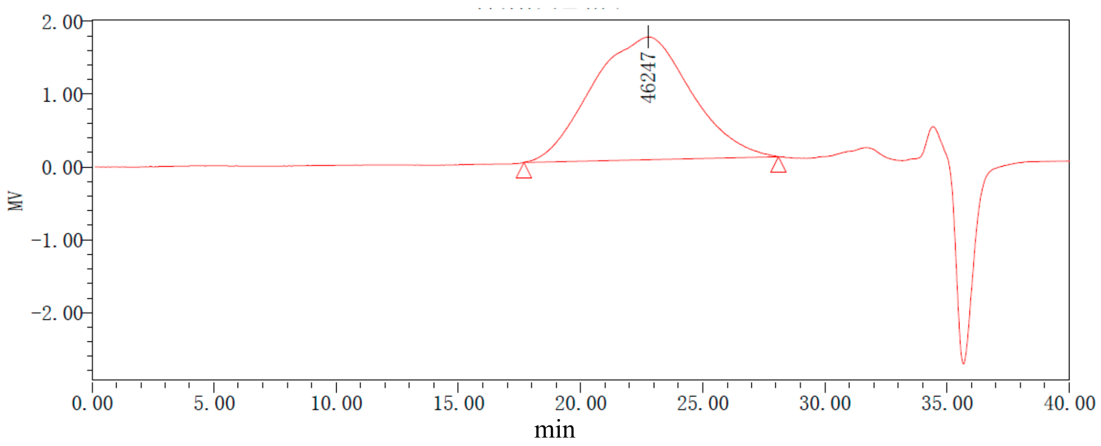

Table S11. GPC results.

M_n (D)	M_w (D)	MP	M_z (D)	$M_z + 1$ (D)	PDI
36968	72112	46247	125805	186522	1.950671

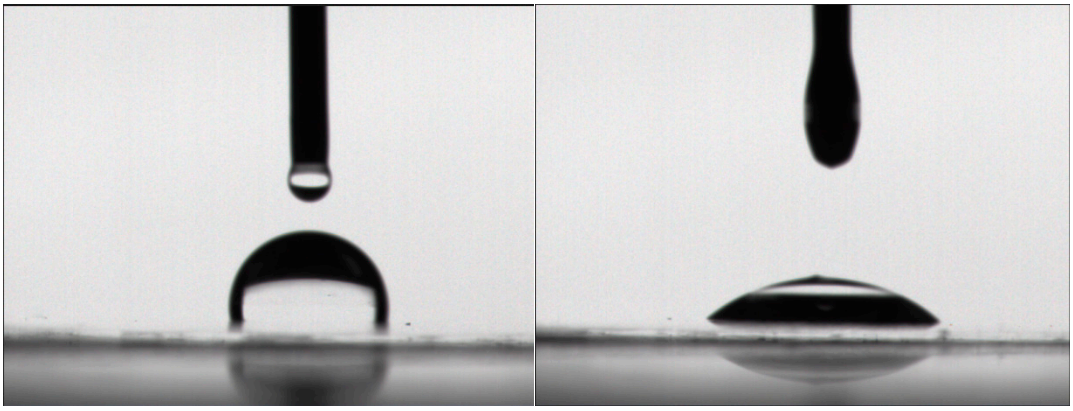

Figure S27. Contact Angle with Water and n-Hexadecane.

14. Compound of Poly(5-co-EMA) (1:8)

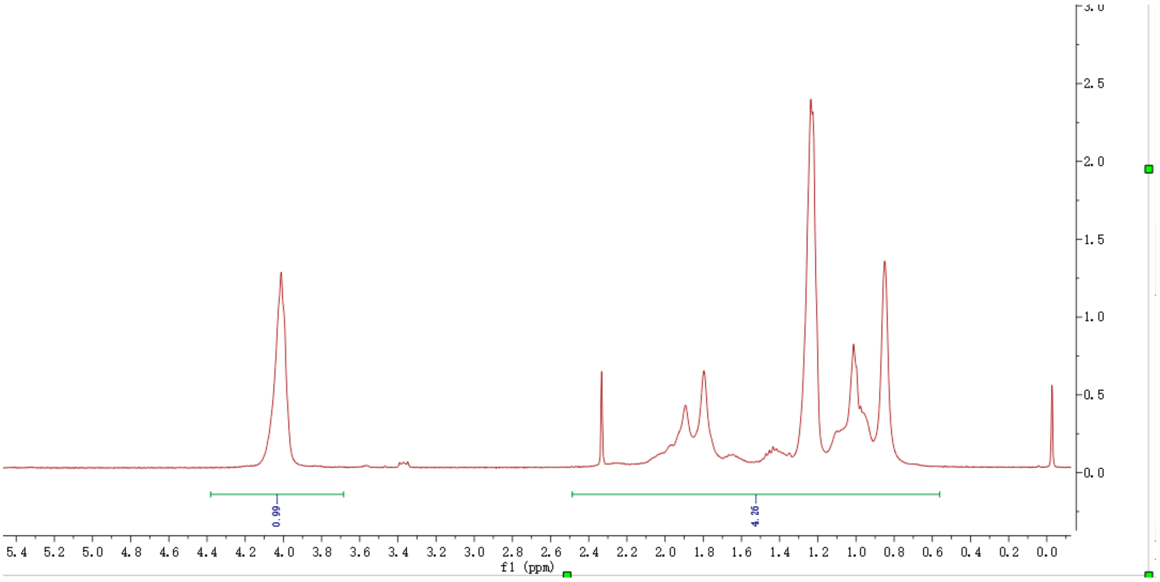

Figure S28. ^{1}H NMR ($CDCl_3$, 400 MHz).

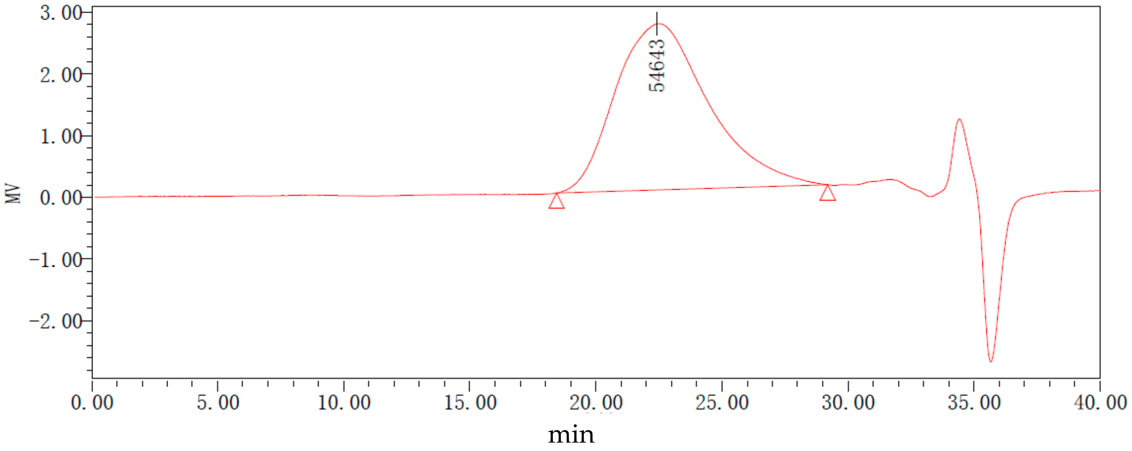

Table S12. GPC results.

M_n (D)	M_w (D)	MP	M_z (D)	$M_z + 1$ (D)	PDI
31278	59834	54643	93593	126984	1.913009

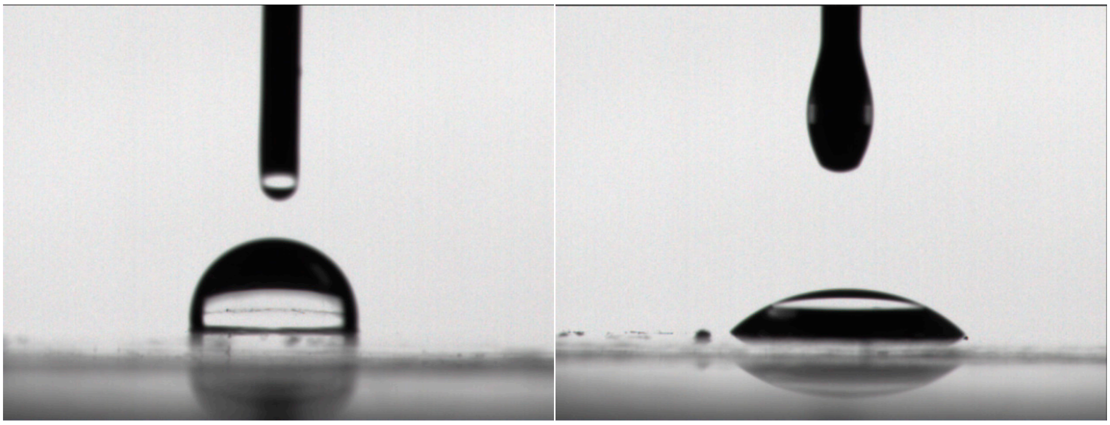

Figure S29. Contact Angle with Water and n-Hexadecane.

15. Compound of Poly(5-co-EMA) (1:6)

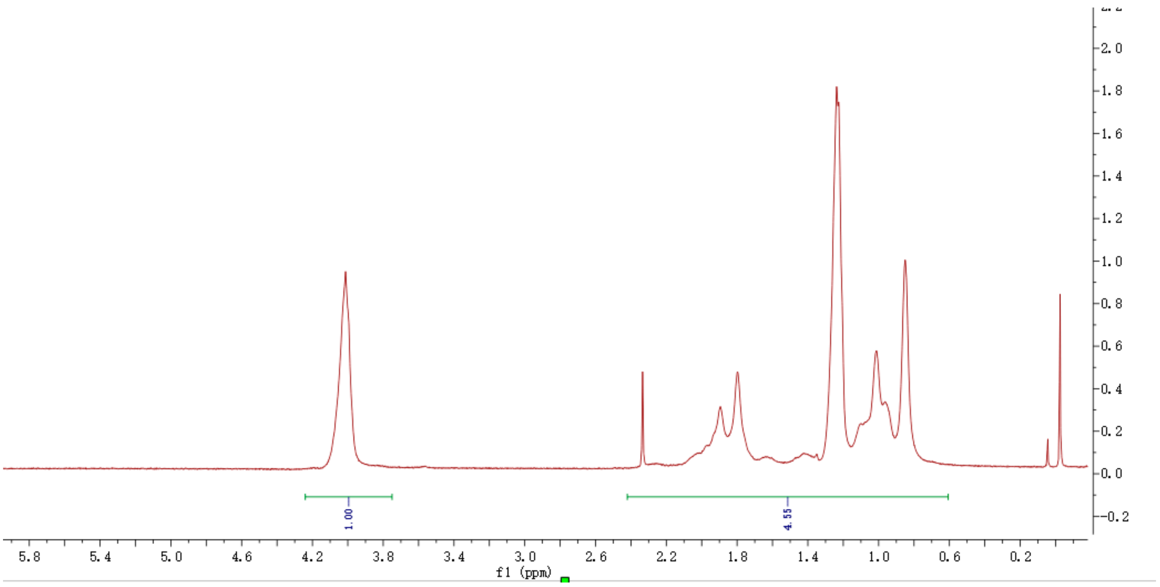

Figure S30.^{1}H NMR ($CDCl_3$, 400 MHz).

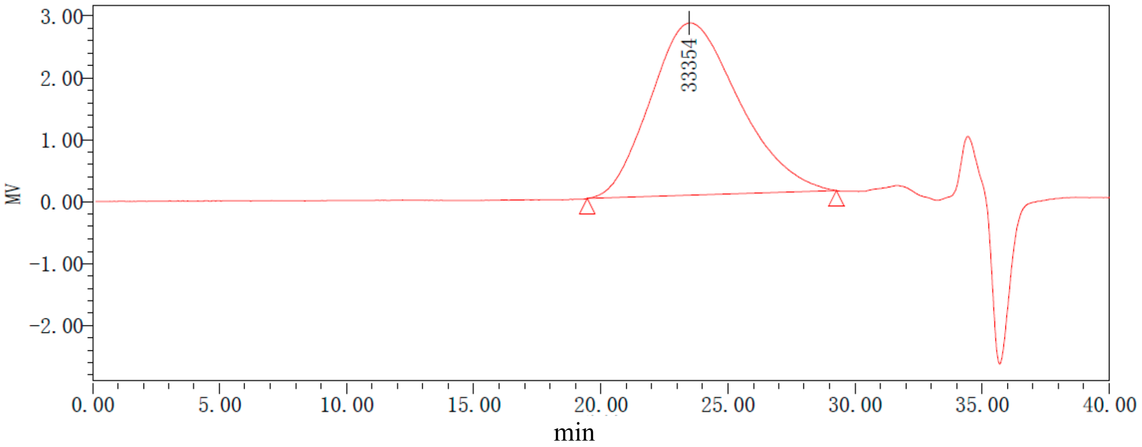

Table S13. GPC results.

M_n (D)	M_w (D)	MP	M_z (D)	$M_z + 1$ (D)	PDI
21391	36209	33354	55343	75681	1.692668

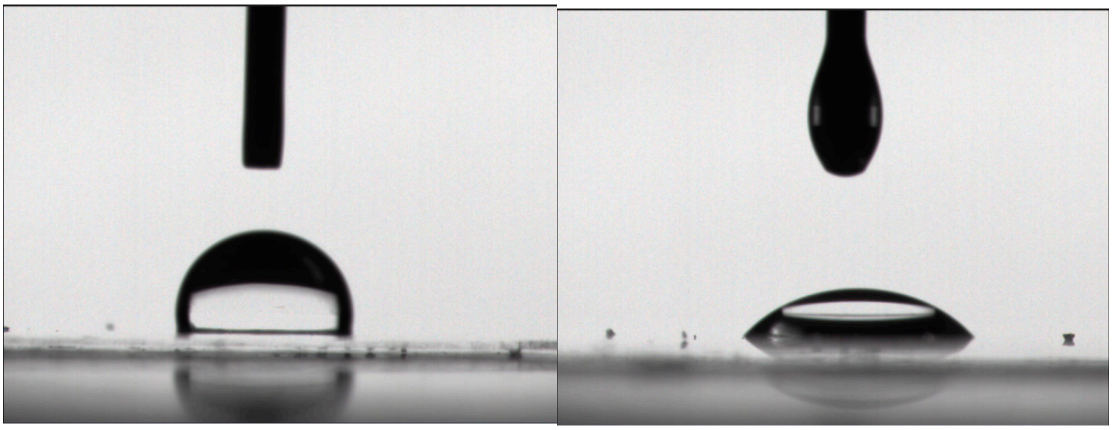

Figure S31. Contact Angle with Water and n-Hexadecane.

16. Compound of Poly(5-co-EMA) (1:2)

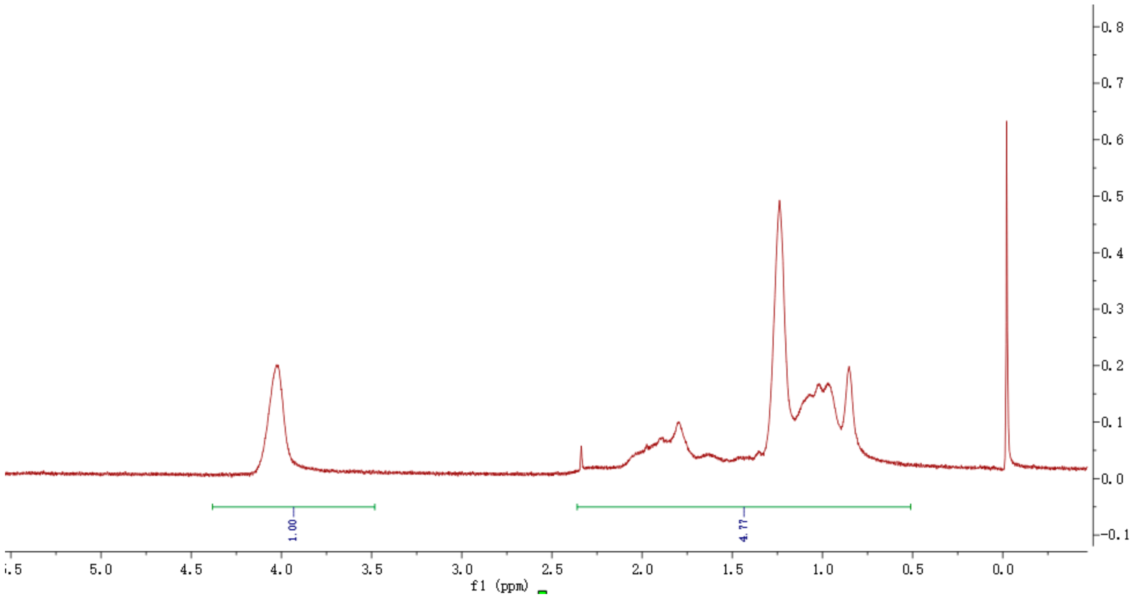

Figure S32.^{1}H NMR ($CDCl_3$, 400 MHz).

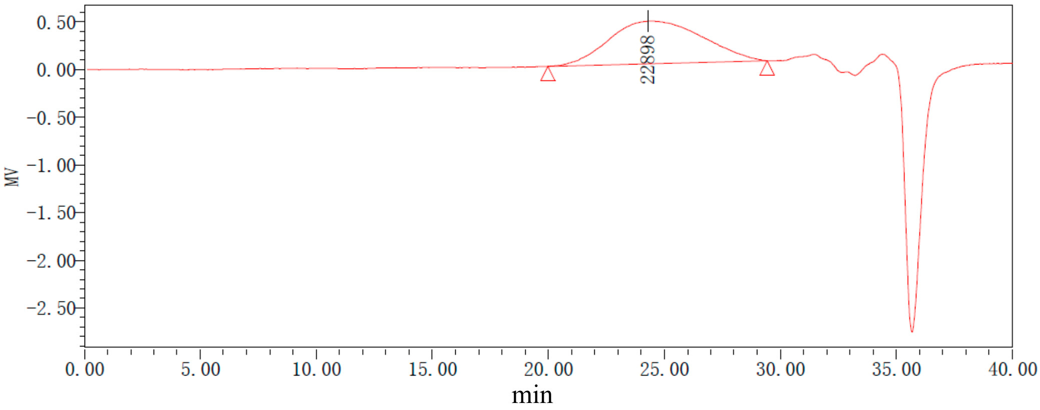

Table S14. GPC results.

M_n (D)	M_w (D)	MP	M_z (D)	$M_z + 1$ (D)	PDI
14538	25291	22898	41182	59177	1.739608

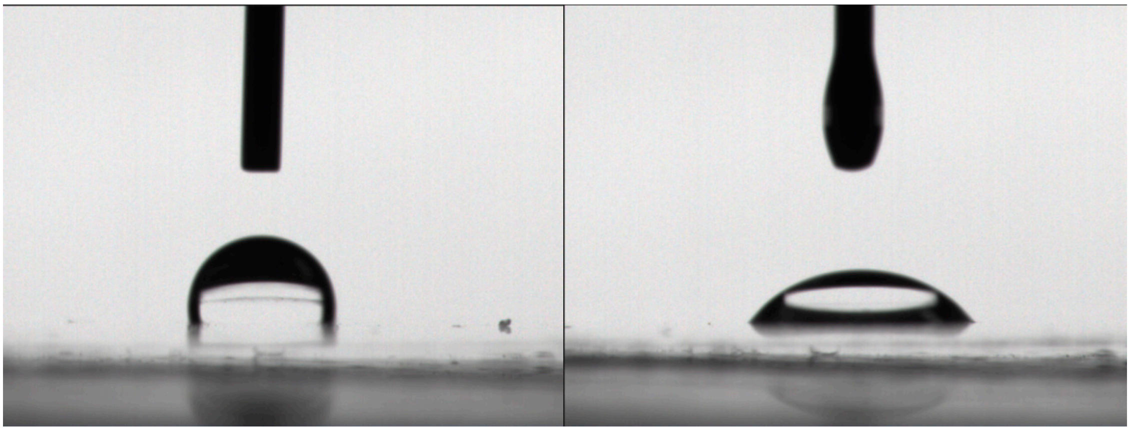

Figure S33. Contact Angle with Water and n-Hexadecane.

17. Compound of Poly(5-co-EMA) (1:1)

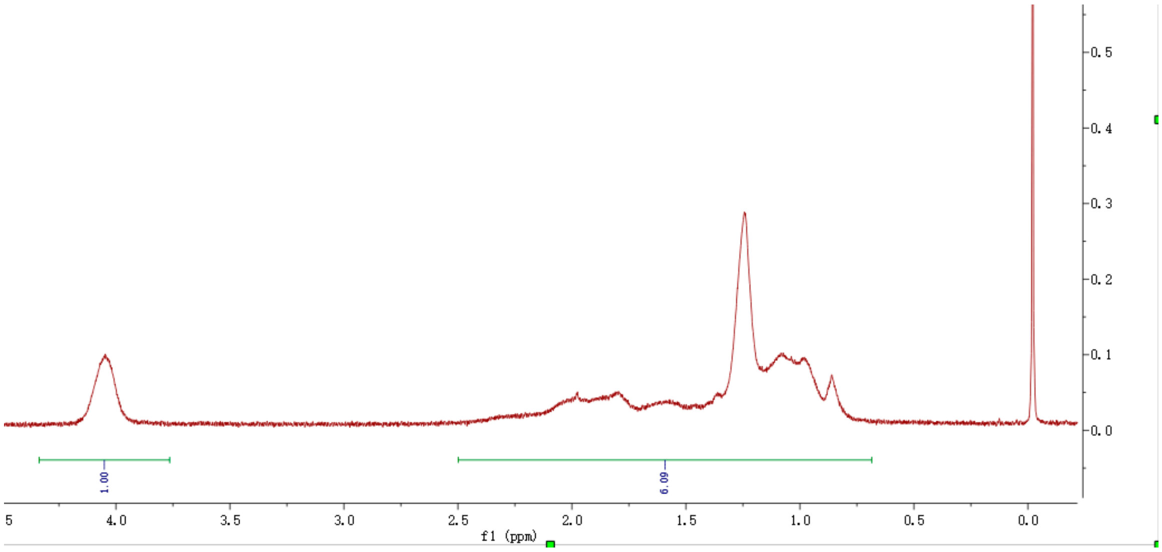

Figure S34.^{1}H NMR ($CDCl_3$, 400 MHz).

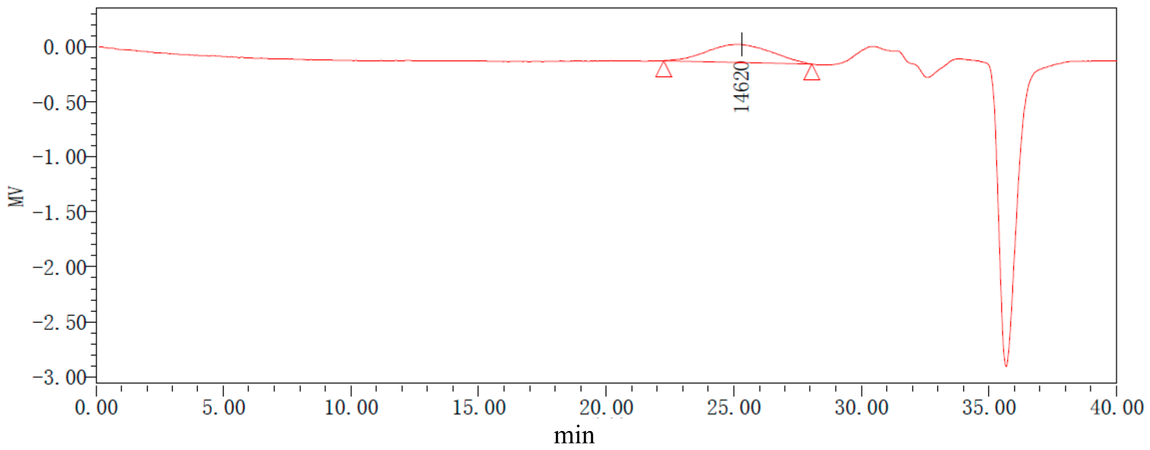

Table S15. GPC results.

M_n (D)	M_w (D)	MP	M_z (D)	M_z + 1 (D)	PDI
13584	16966	14620	21232	26051	1.248996

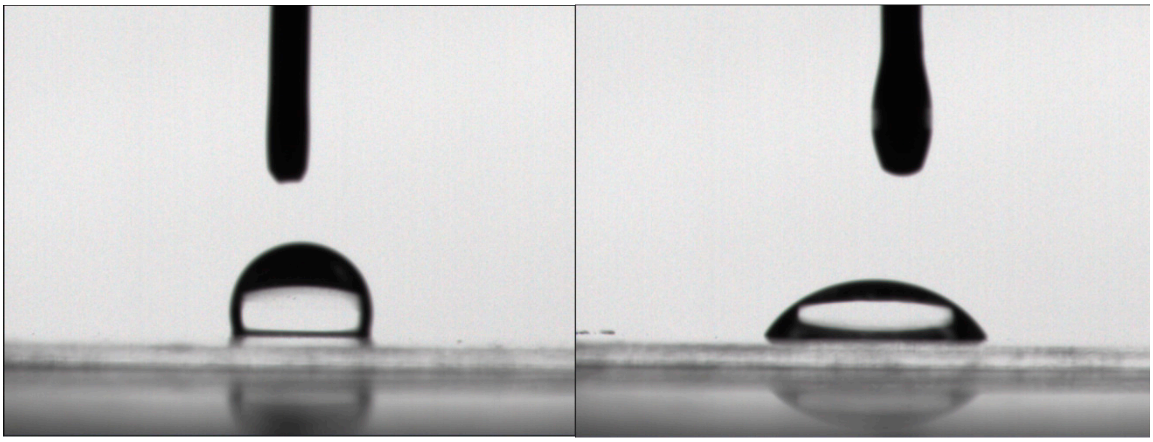

Figure S35. Contact Angle with Water and n-Hexadecane.

18. Compound of Poly(5-co-HMA) (1:15)

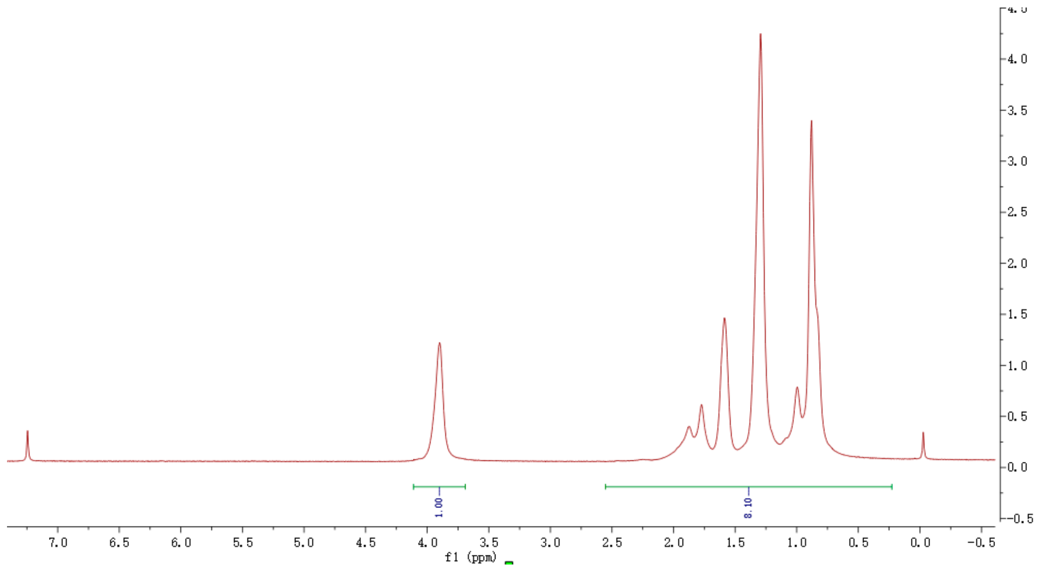

Figure S36. ^{1}H NMR ($CDCl_3$, 400 MHz).

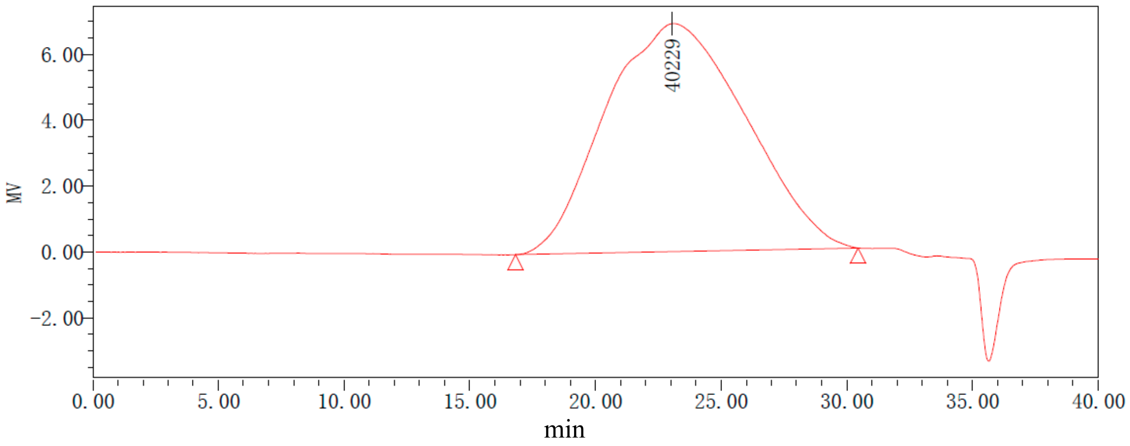

Table S16. GPC results.

M_n (D)	M_w (D)	MP	M_z (D)	$M_z + 1$ (D)	PDI
20518	64145	40229	152163	260728	3.126285

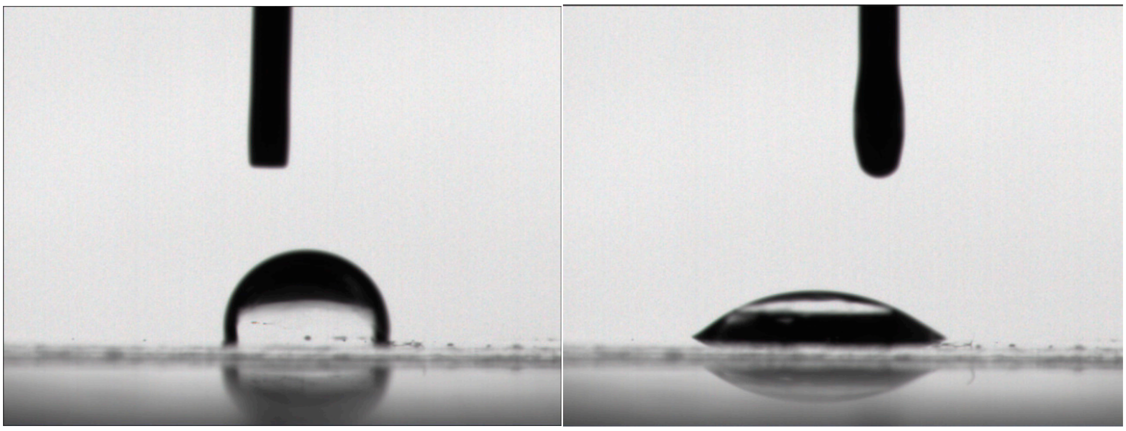

Figure S37. Contact Angle with Water and n-Hexadecane.

19. C.ompound of Poly(5-co-HMA) (1:8)

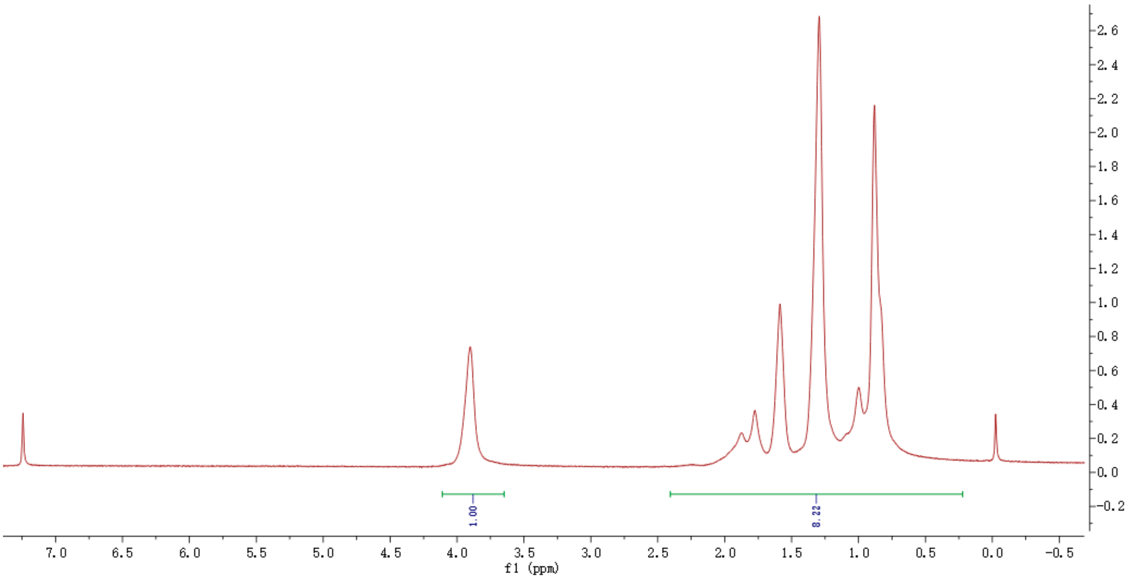

Figure S38. ^{1}H NMR ($CDCl_3$, 400 MHz).

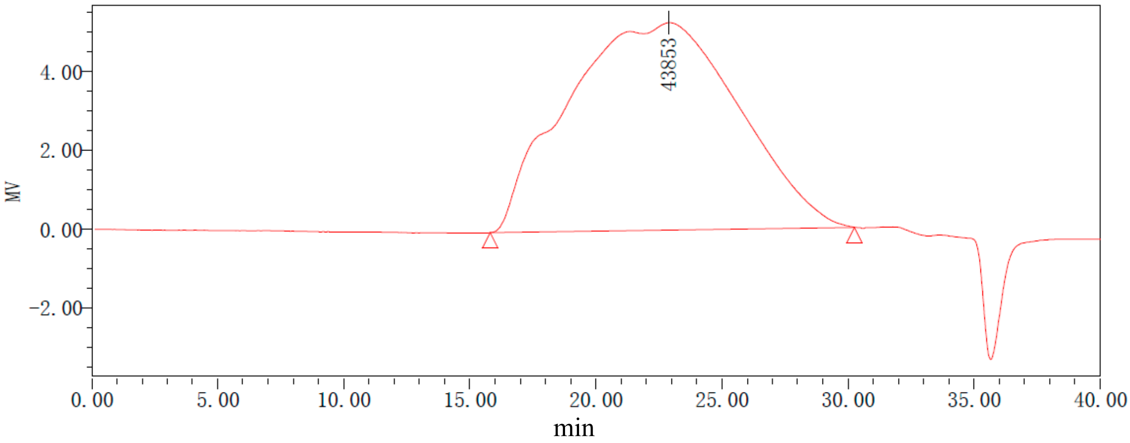

Table S17. GPC results.

M_n (D)	M_w (D)	MP	M_z (D)	$M_z + 1$ (D)	PDI
27071	138428	43853	468579	848319	5.113542

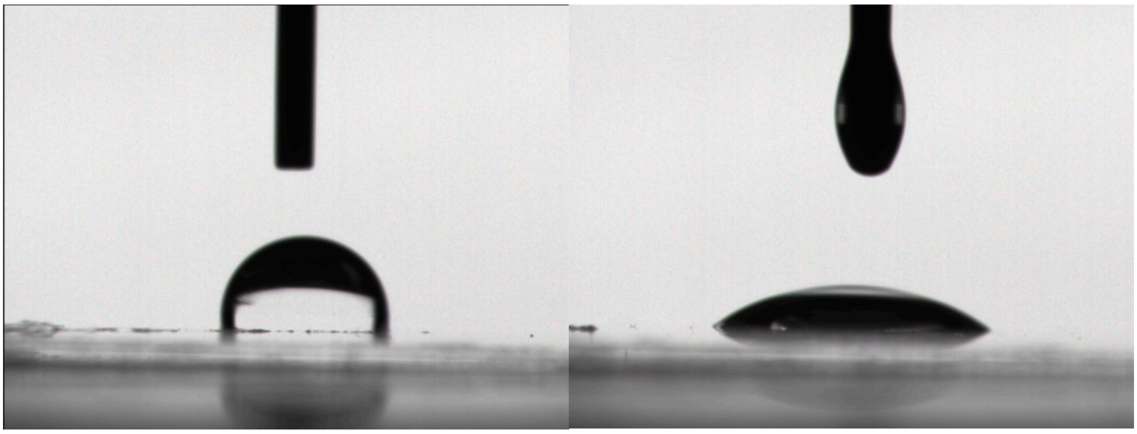

Figure S39. Contact Angle with Water and n-Hexadecane.

20. Compound of Poly(5-co-HMA) (1:6)

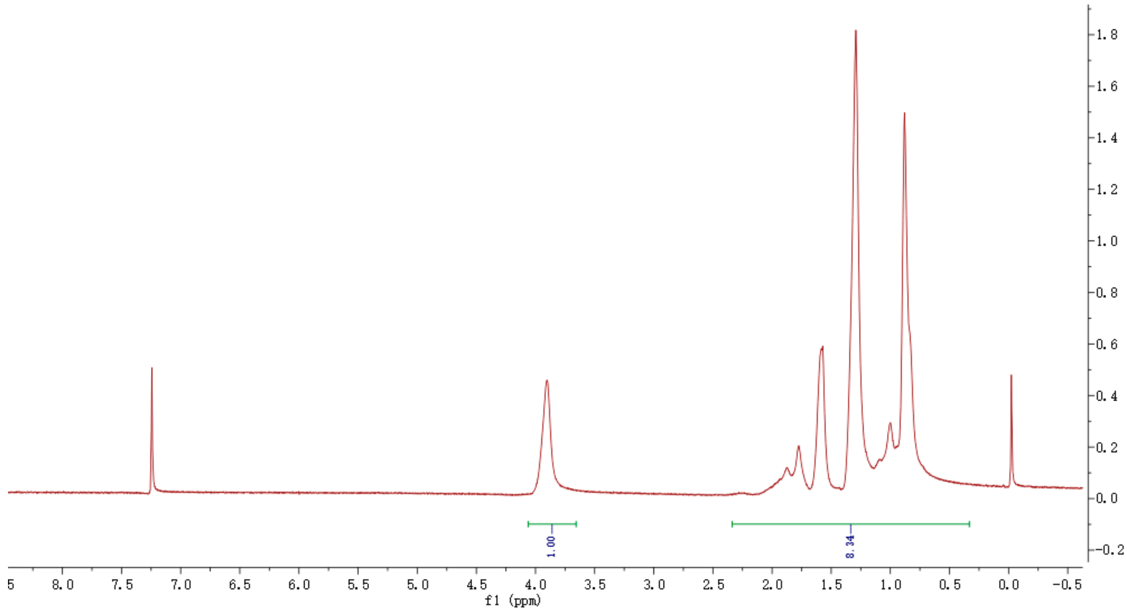

Figure S40. ¹H NMR ($CDCl_3$, 400 MHz).

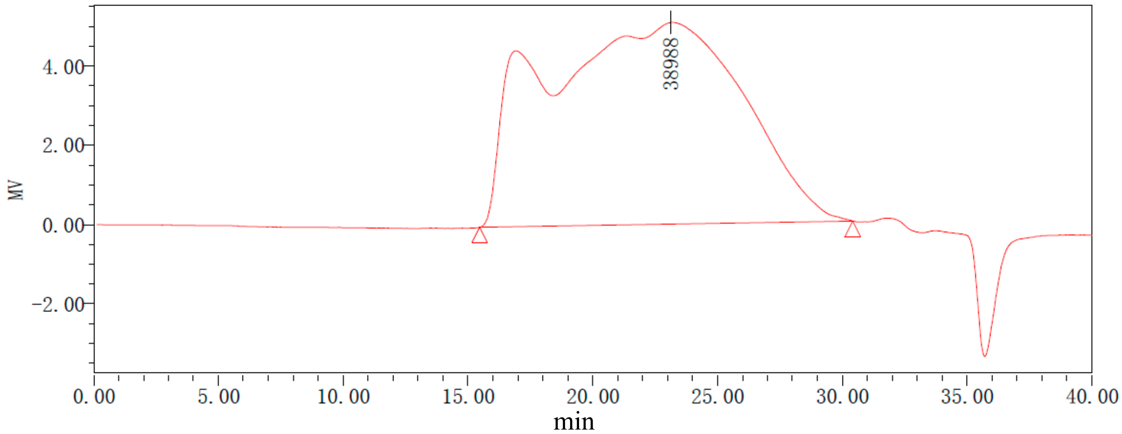

Table S18. GPC results.

M_n (D)	M_w (D)	MP	M_z (D)	$M_z + 1$ (D)	PDI
27239	240433	38988	938870	1493989	8.826767

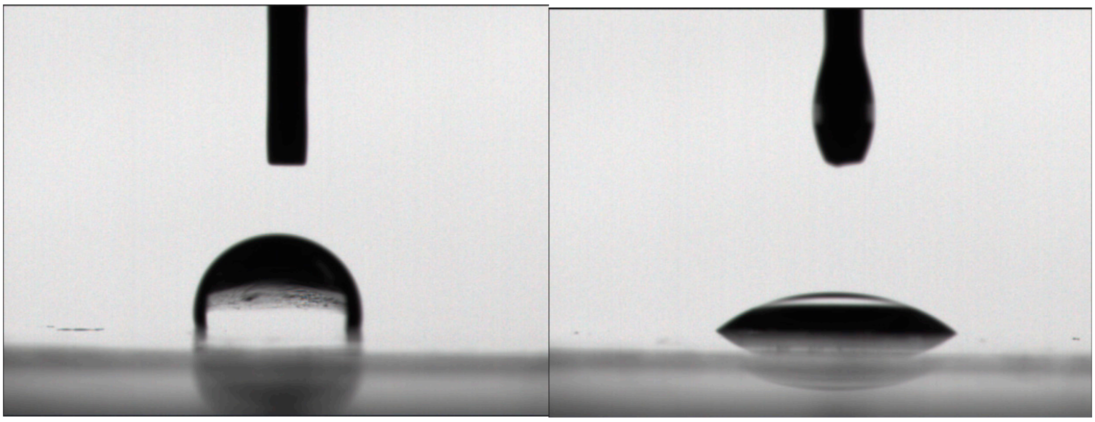

Figure S41Contact Angle with Water and n-Hexadecane.

21. Compound of Poly(5-co-HMA) (1:2)

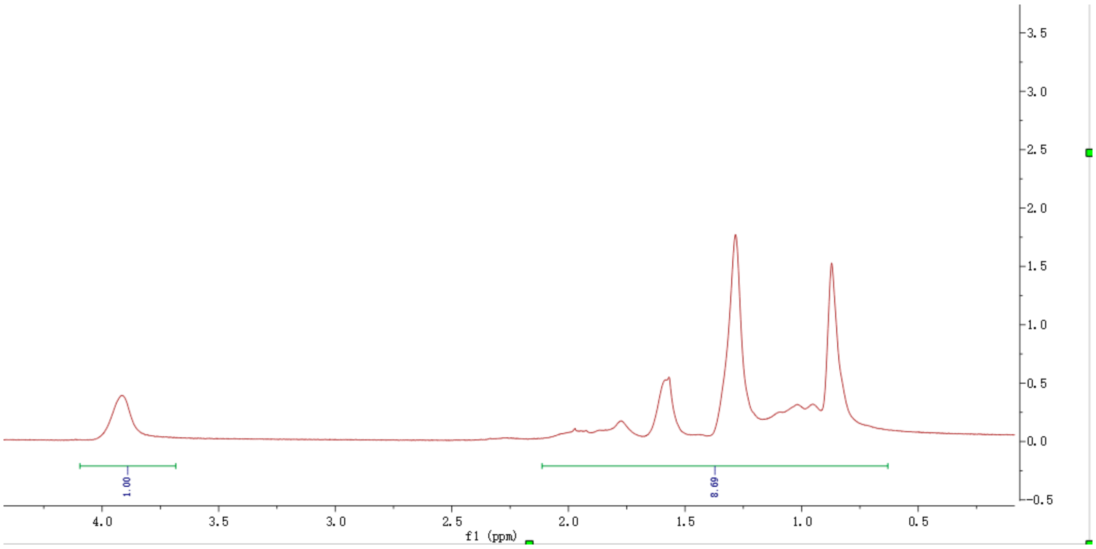

Figure S42. ^1H NMR ($CDCl_3$, 400 MHz).

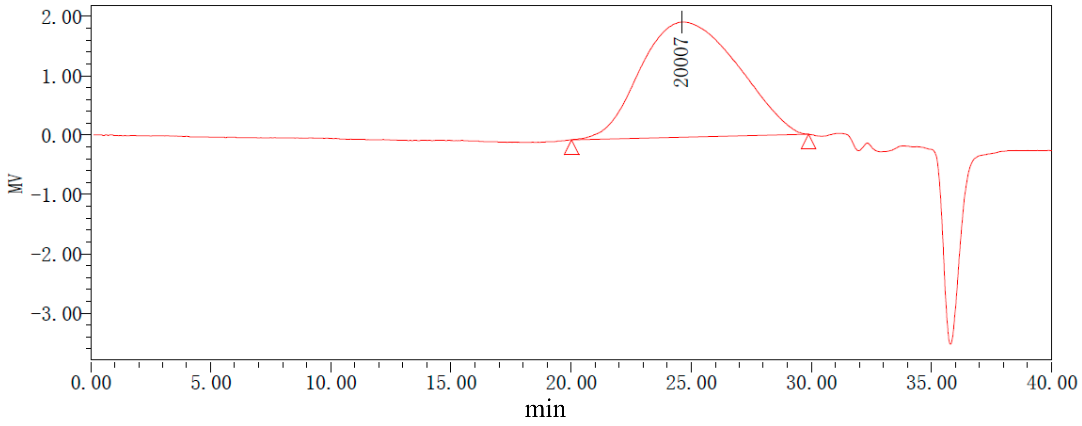

Table S19. GPC results.

M_n (D)	M_w (D)	MP	M_z (D)	$M_z + 1$ (D)	PDI
12680	22058	20007	35839	51788	1.739677

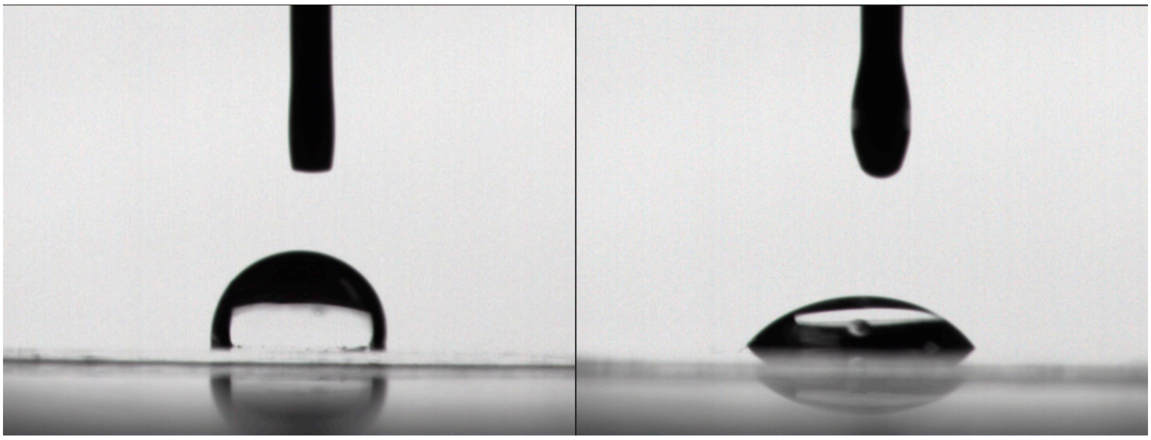

Figure S43. Contact Angle with Water and n-Hexadecane.

22. Compound of Poly(5-co-HMA) (1:1)

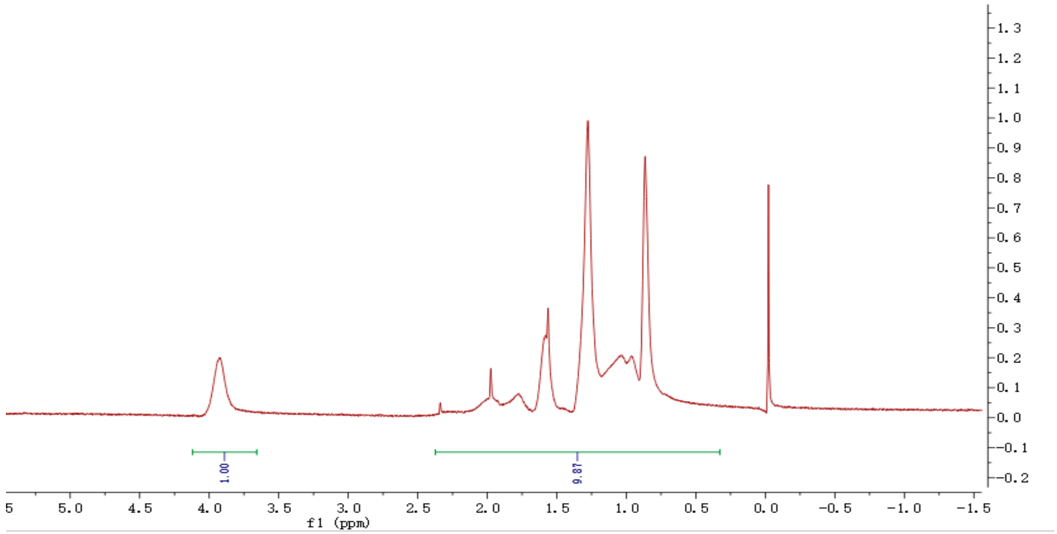

Figure S44. ^1H NMR ($CDCl_3$, 400 MHz).

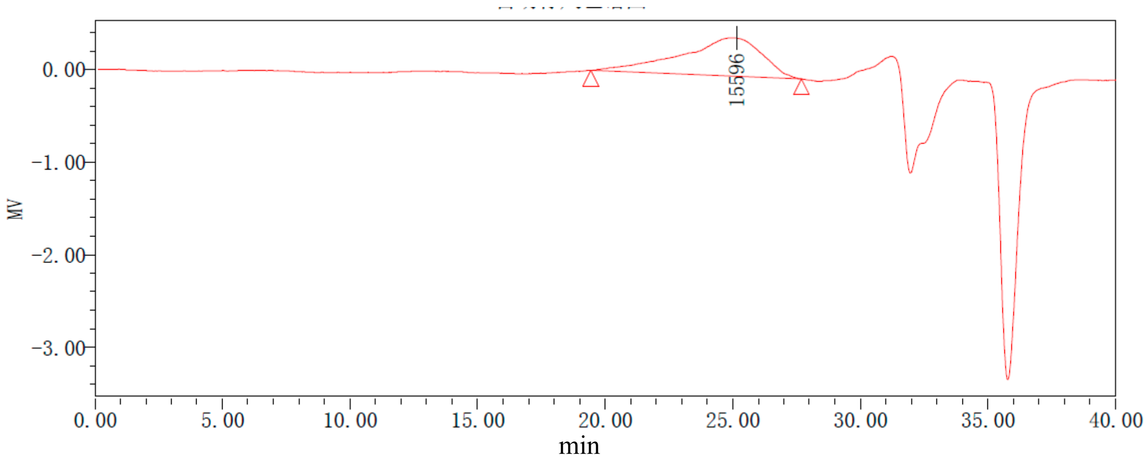

Table S20. GPC results.

M_n (D)	M_w (D)	MP	M_z (D)	$M_z + 1$ (D)	PDI
19019	31622	15596	58417	92315	1.662623

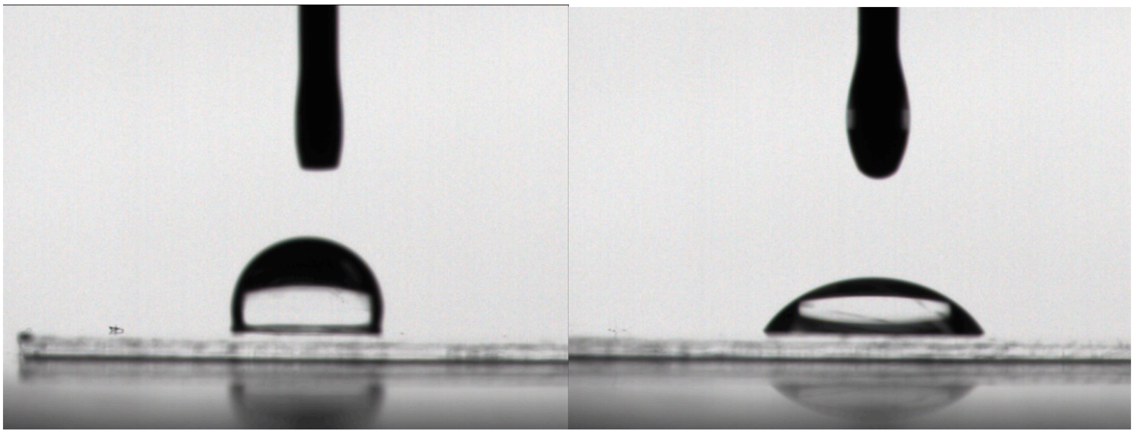

Figure S45. Contact Angle with Water and n-Hexadecane.

23. Compound of Poly(5-co-OMA) (1:15)

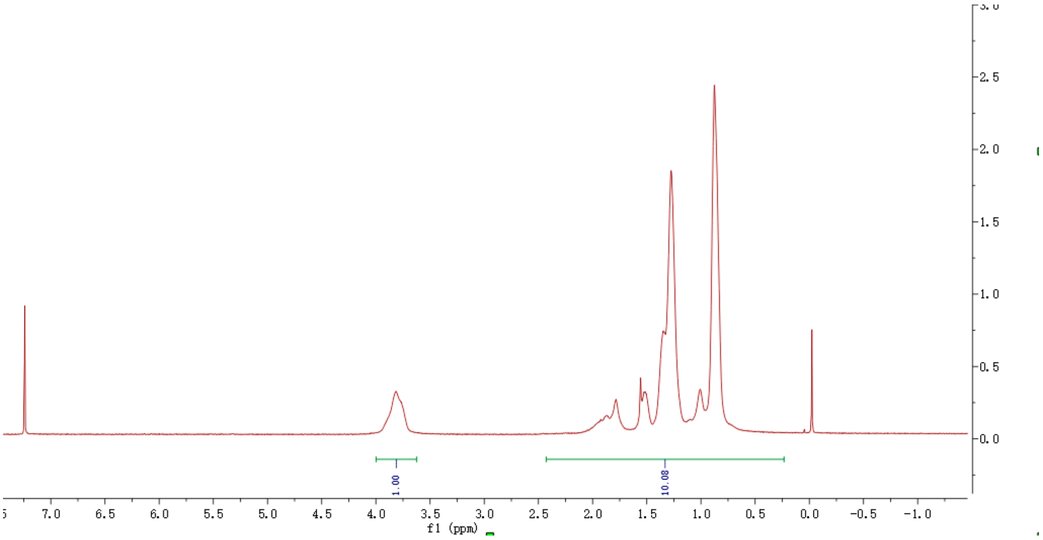

Figure S46. ^1H NMR ($CDCl_3$, 400 MHz).

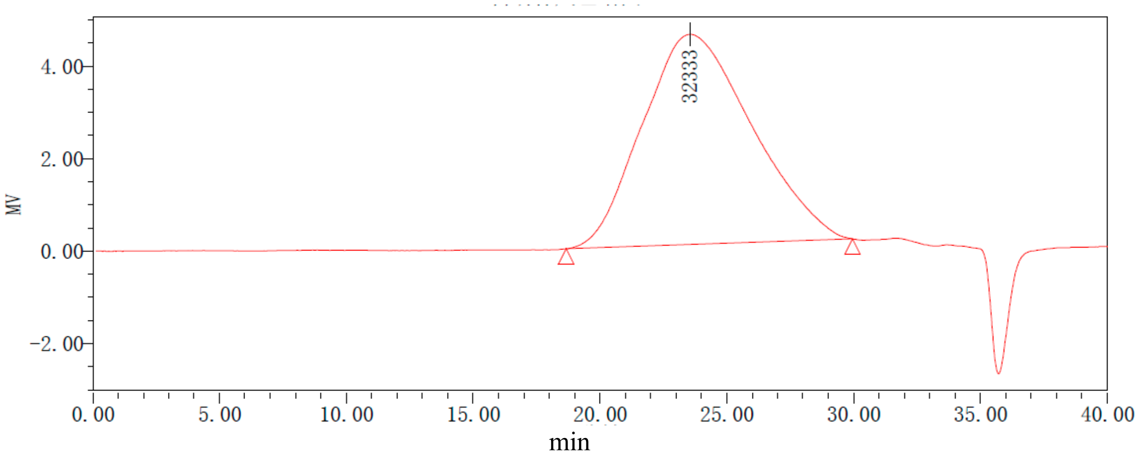

Table S21. GPC results.

M_n (D)	M_w (D)	MP	M_z (D)	$M_z + 1$ (D)	PDI
18079	38266	32333	69065	102692	2.116577

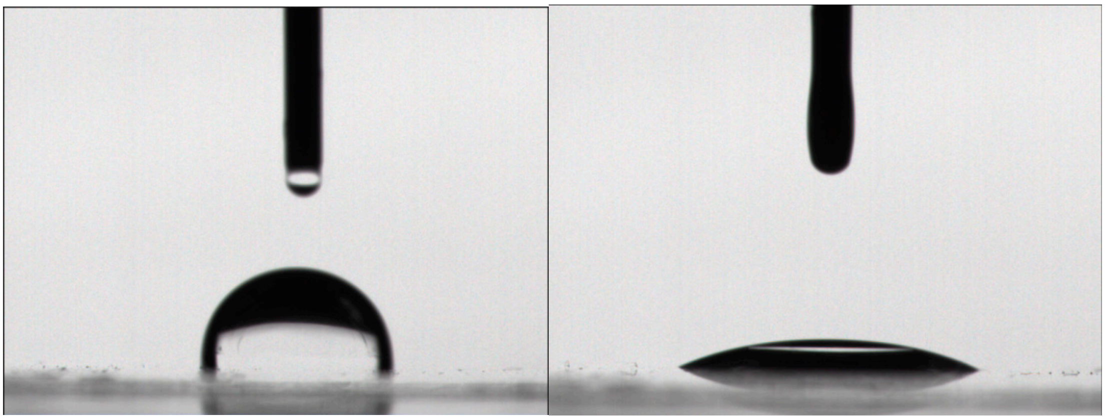

Figure S47. Contact Angle with Water and n-Hexadecane.

24. Compound of Poly(5-co-OMA) (1:8)

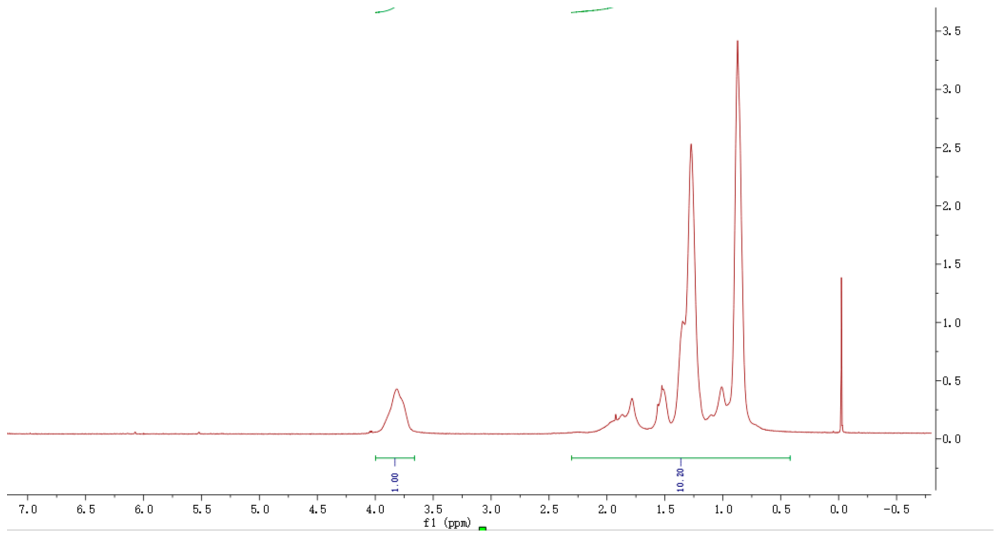

Figure S48. ^{1}H NMR ($CDCl_3$, 400 MHz).

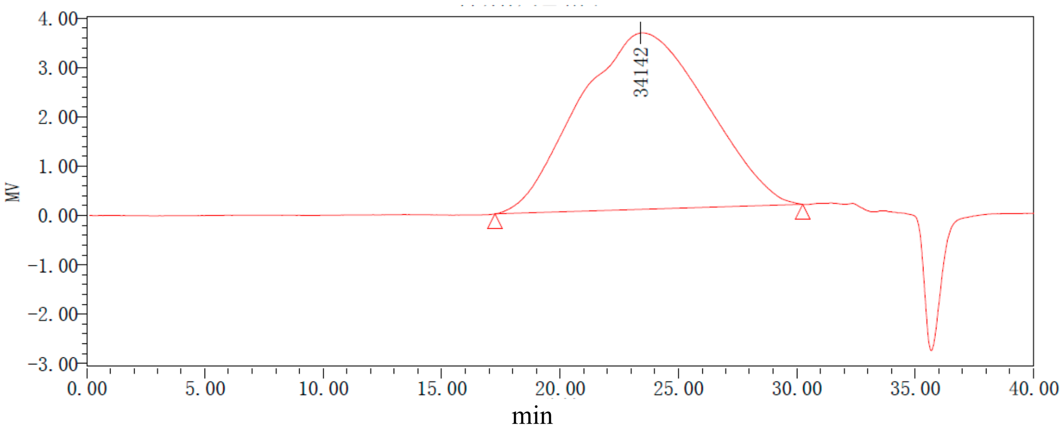

Table S22. GPC results.

M_n (D)	M_w (D)	MP	M_z (D)	$M_z + 1$ (D)	PDI
19372	56644	34142	133017	222816	2.924048

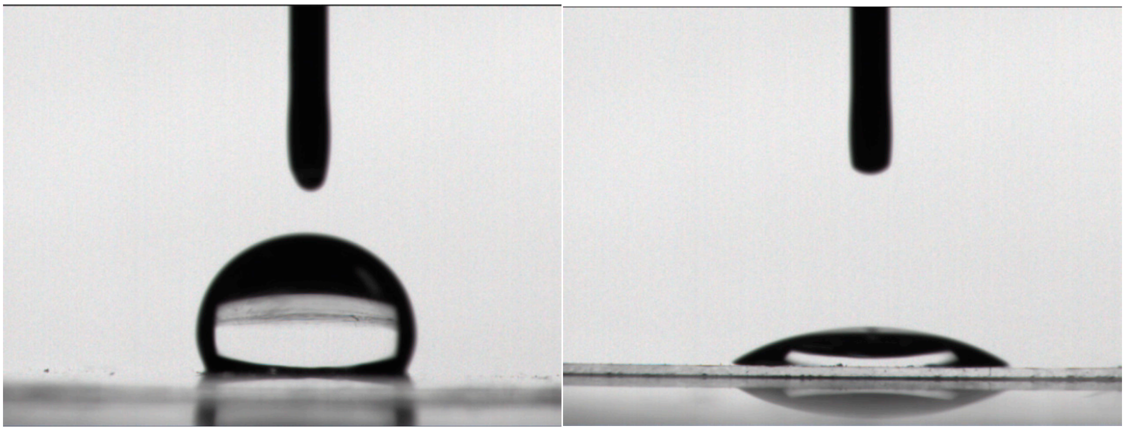

Figure S49. Contact Angle with Water and n-Hexadecane.

25. Compound of Poly(5-co-OMA) (1:6)

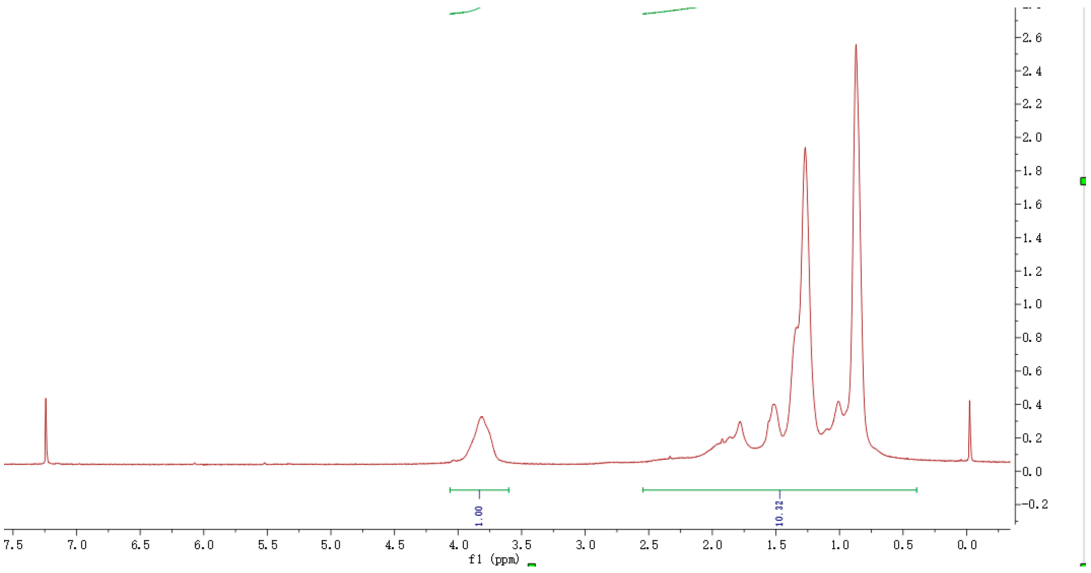

Figure S50. ^1H NMR ($CDCl_3$, 400 MHz).

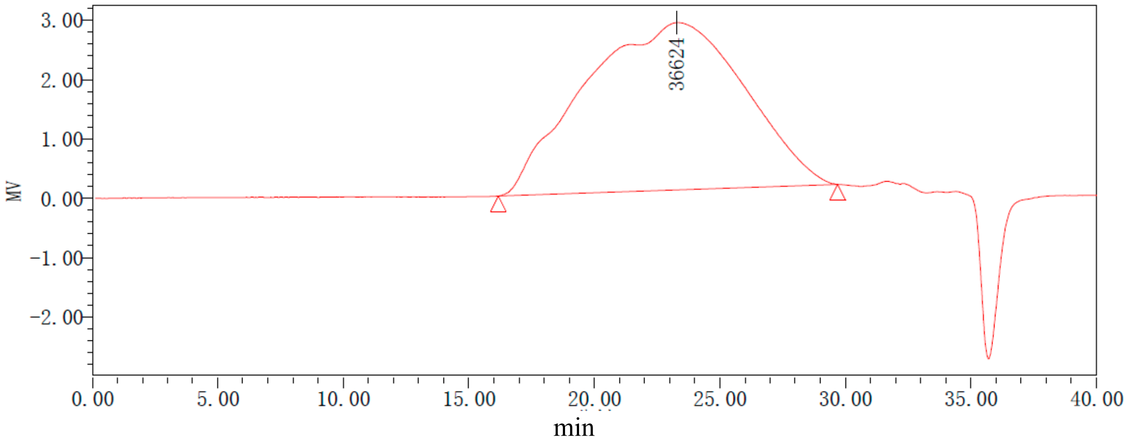

Table S23. GPC results.

M_n (D)	M_w (D)	MP	M_z (D)	$M_z + 1$ (D)	PDI
24889	102840	36624	315148	564801	4.131881

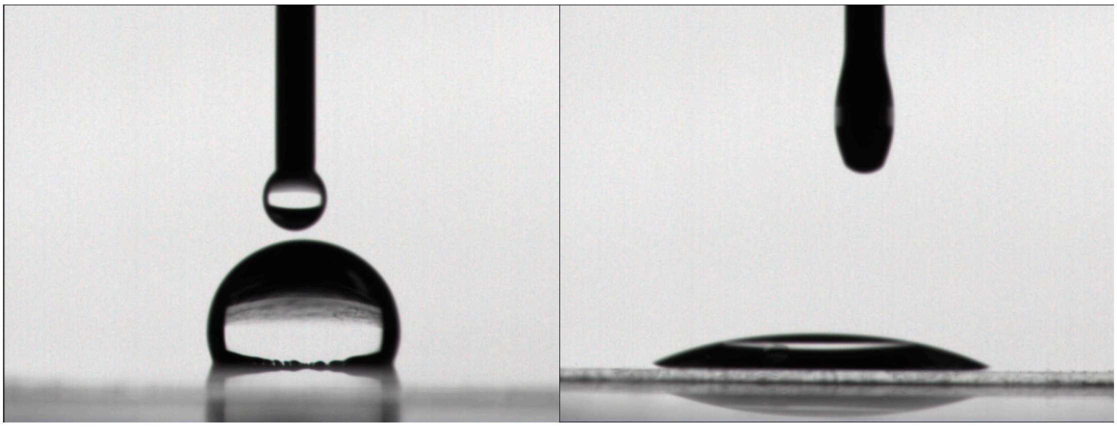

Figure S51. Contact Angle with Water and n-Hexadecane.

26. Compound of Poly(5-co-OMA) (1:2)

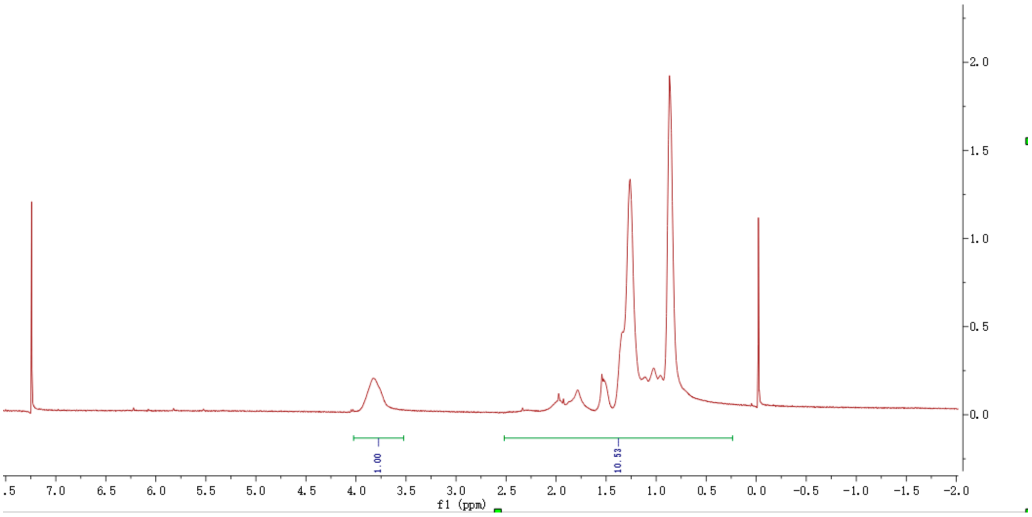

Figure S52. ^1H NMR ($CDCl_3$, 400 MHz).

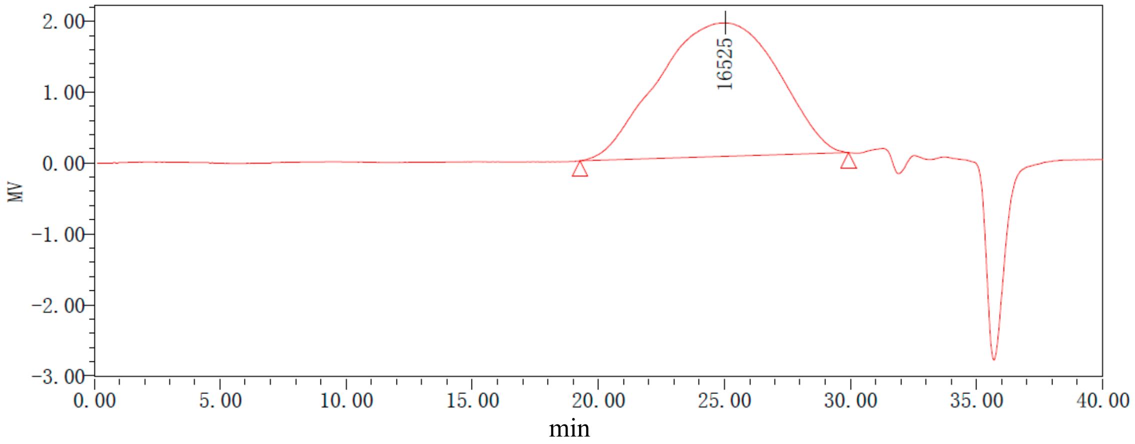

Table S24. GPC results.

M_n (D)	M_w (D)	MP	M_z (D)	$M_z + 1$ (D)	PDI
13677	28256	16525	54506	83455	2.066034

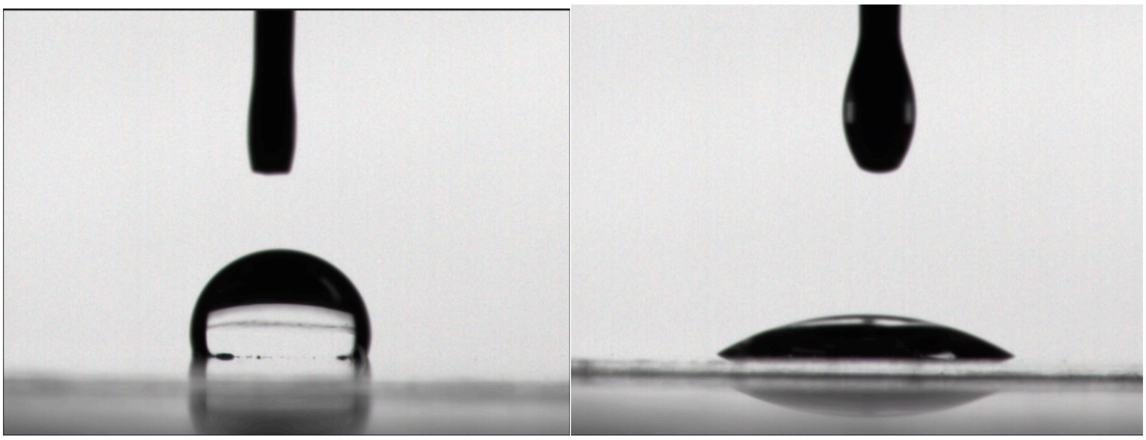

Figure S53. Contact Angle with Water and n-Hexadecane.

27. Compound of Poly(5-co-OMA) (1:1)

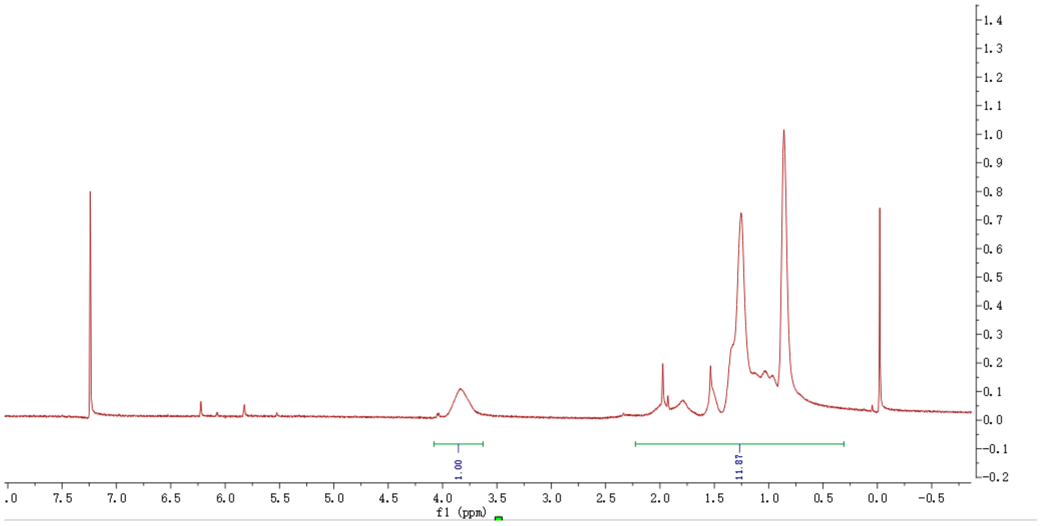

Figure S54. ^1H NMR ($CDCl_3$, 400 MHz).

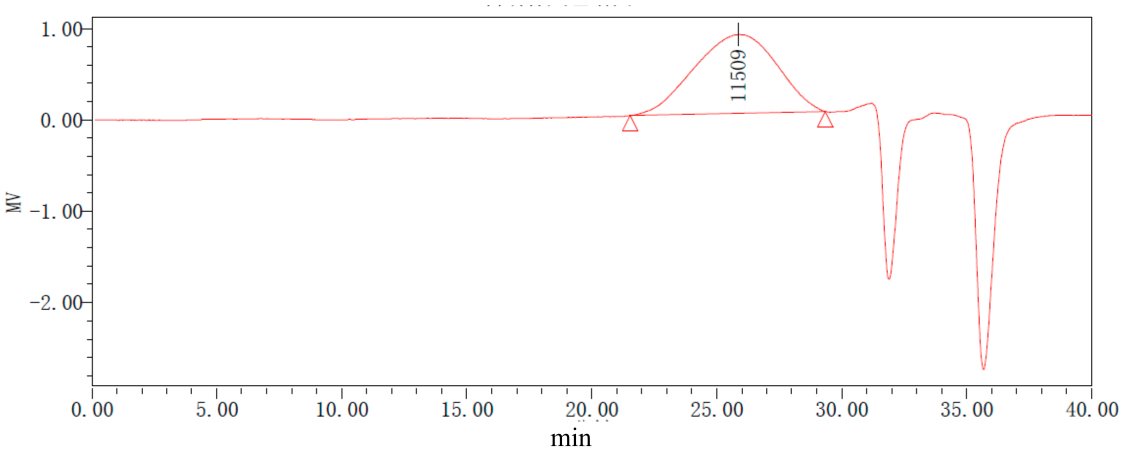

Table S25. GPC results.

M_n (D)	M_w (D)	MP	M_z (D)	$M_z + 1$ (D)	PDI
10396	15011	11509	21769	29876	1.444013

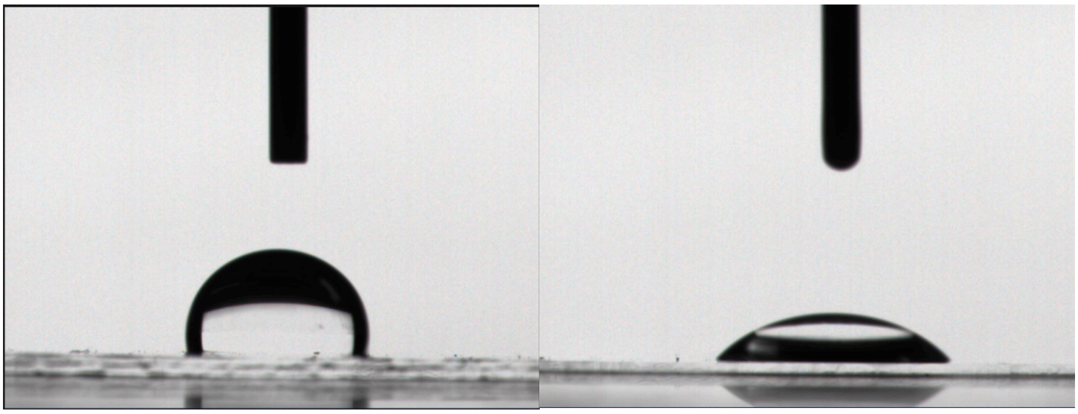

Figure S55. Contact Angle with Water and n-Hexadecane.

28. Compound of Poly(5-co-LMA) (1:15)

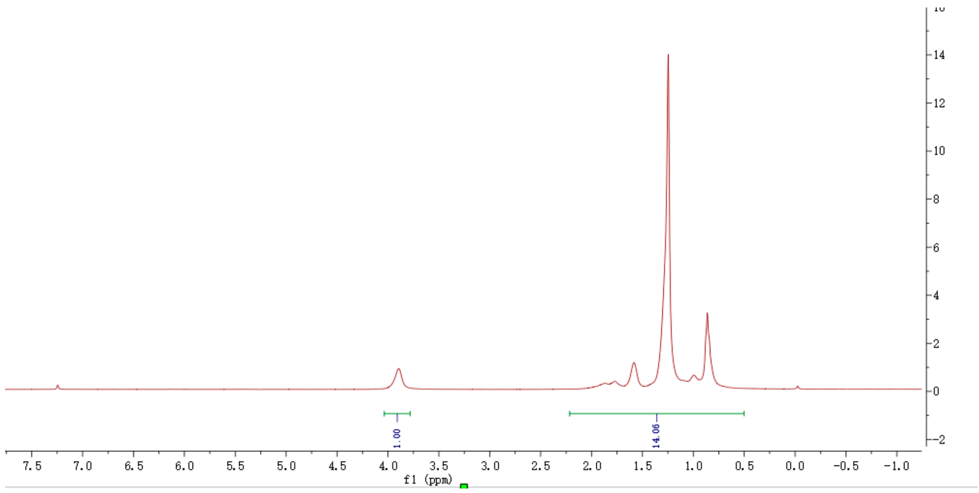

Figure S56. ^{1}H NMR ($CDCl_3$, 400 MHz).

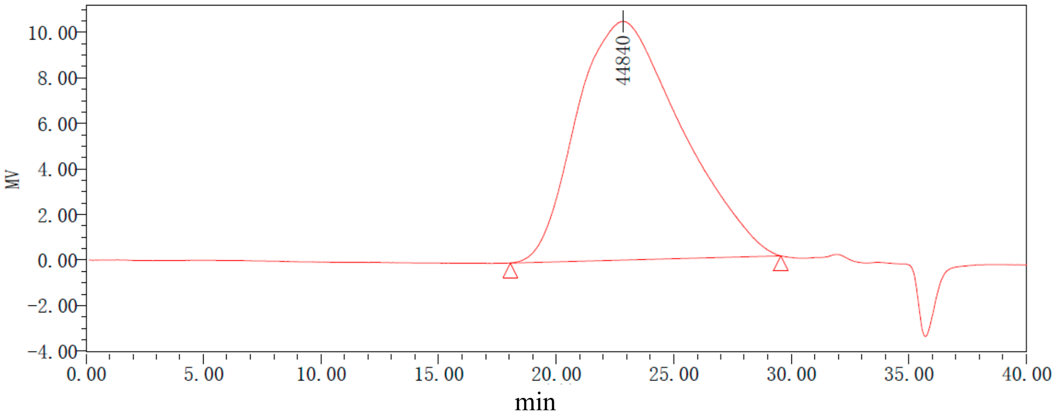

Table S26. GPC results.

M_n (D)	M_w (D)	MP	M_z (D)	M_z + 1 (D)	PDI
23063	51761	44840	91655	132066	2.244307

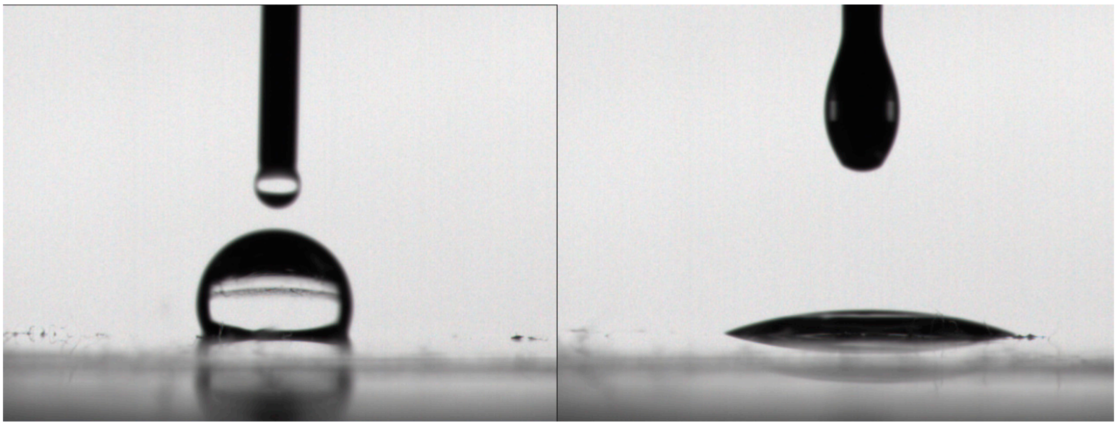

Figure S57. Contact Angle with Water and n-Hexadecane.

29. Compound of Poly(5-co-LMA) (1:8)

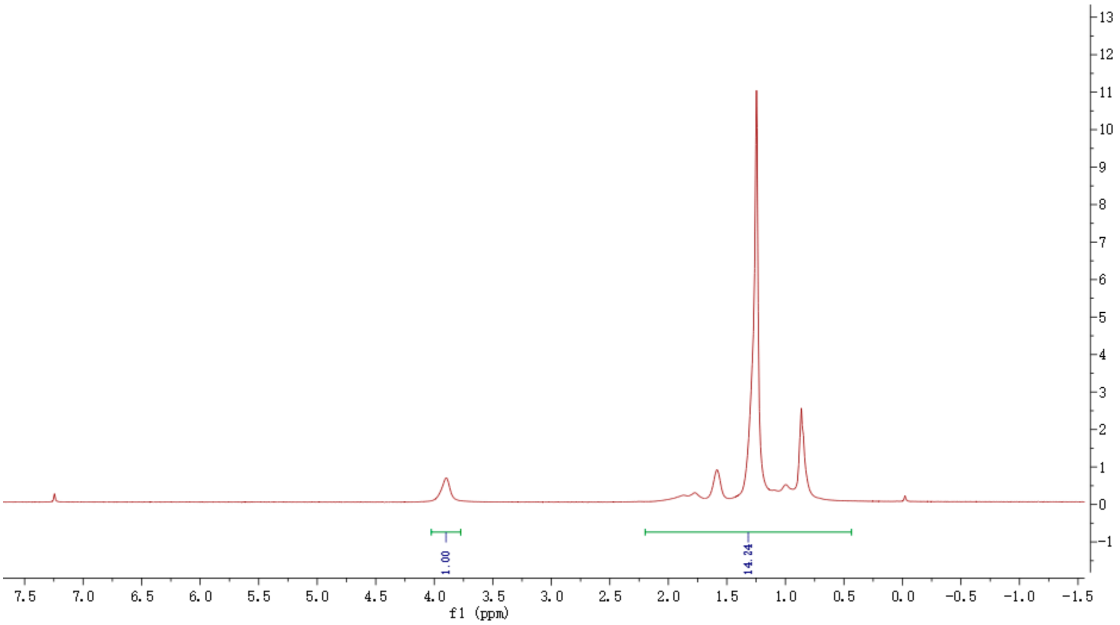

Figure S58. ^1H NMR ($CDCl_3$, 400 MHz).

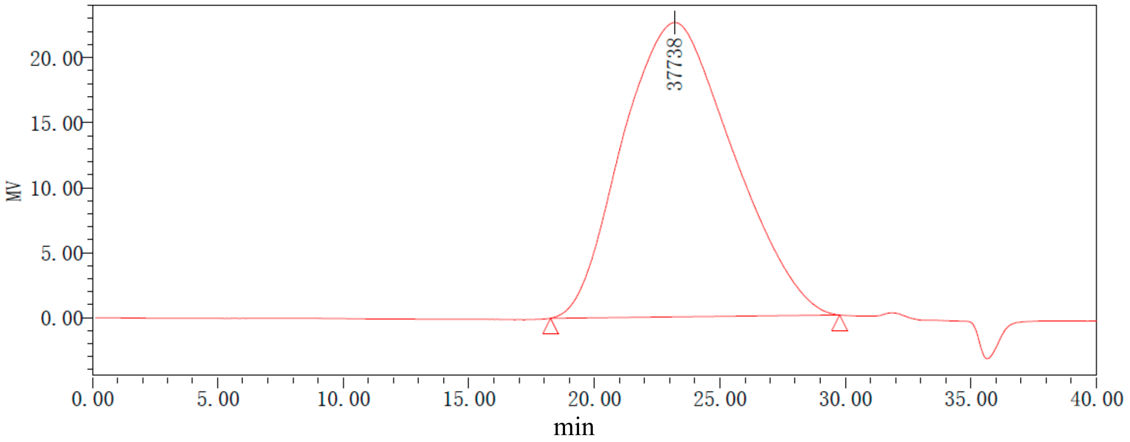

Table S27. GPC results.

M_n (D)	M_w (D)	MP	M_z (D)	$M_z + 1$ (D)	PDI
22597	48628	39495	87725	129197	2.151993

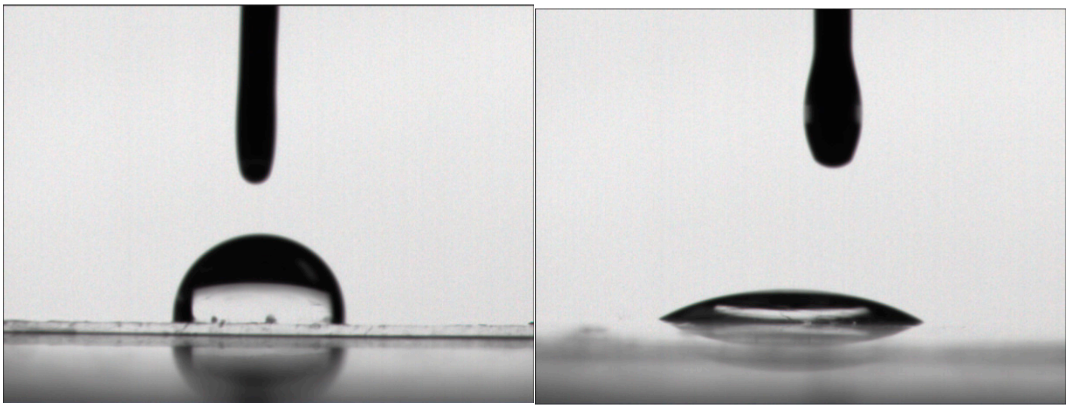

Figure S59. Contact Angle with Water and n-Hexadecane.

30. Compound of Poly(5-co-LMA) (1:6)

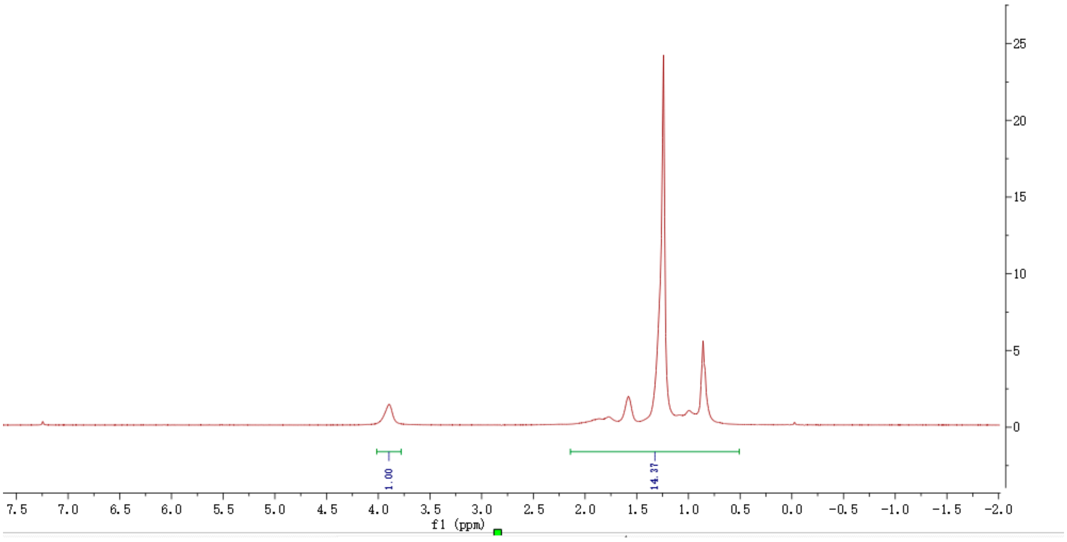

Figure S60. ^1H NMR ($CDCl_3$, 400 MHz).

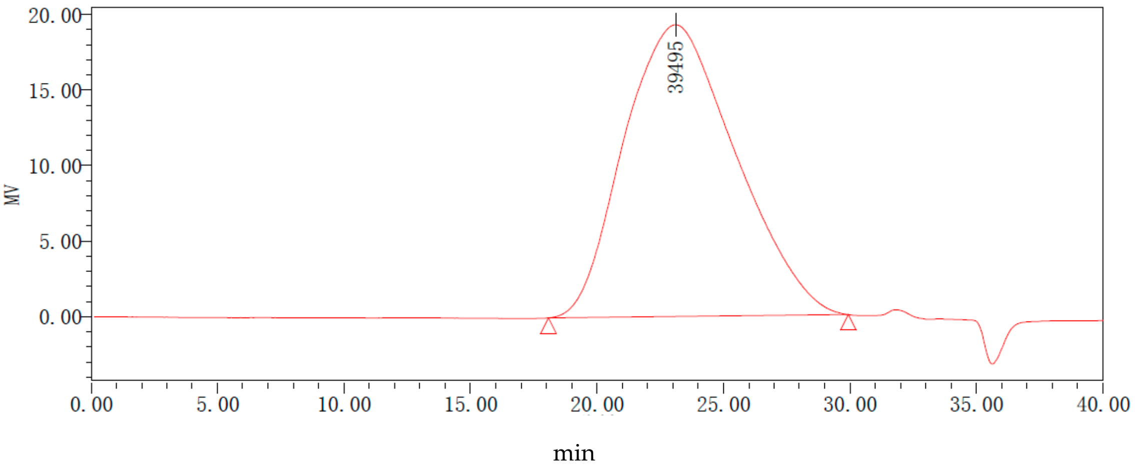

Table S28. GPC results.

M_n (D)	M_w (D)	MP	M_z (D)	$M_z + 1$ (D)	PDI
22431	49283	39495	88874	130985	2.197061

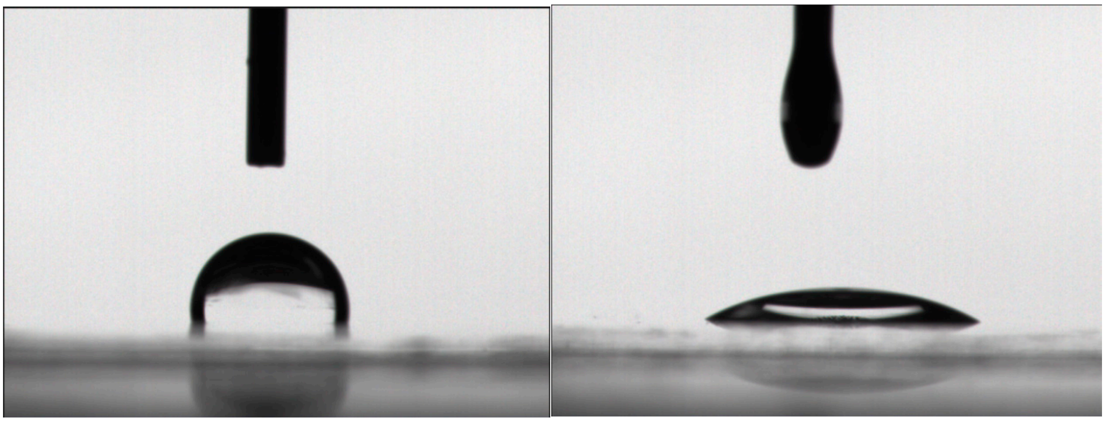

Figure S61. Contact Angle with Water and n-Hexadecane.

31. Compound of Poly(5-co-LMA) (1:2)

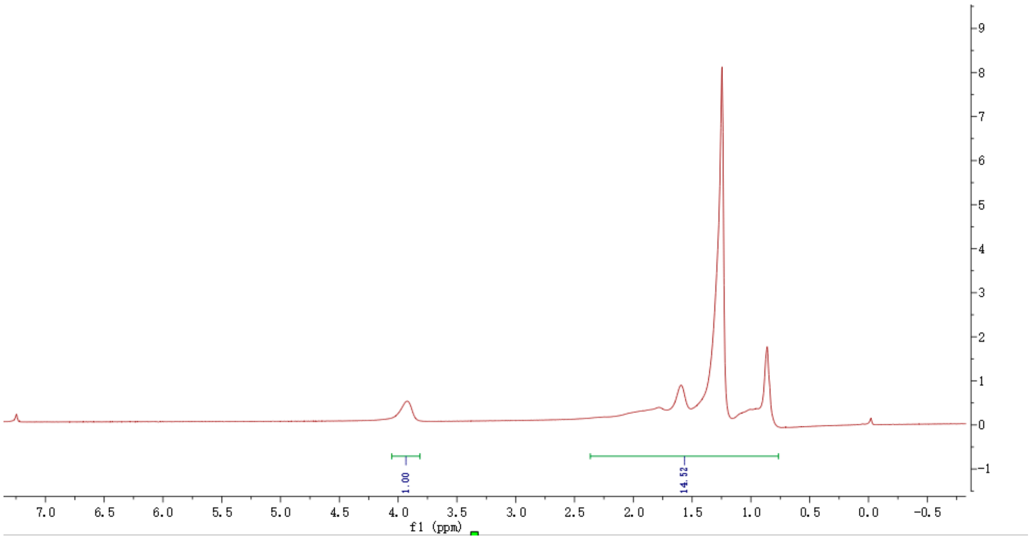

Figure S62. ^1H NMR ($CDCl_3$, 400 MHz).

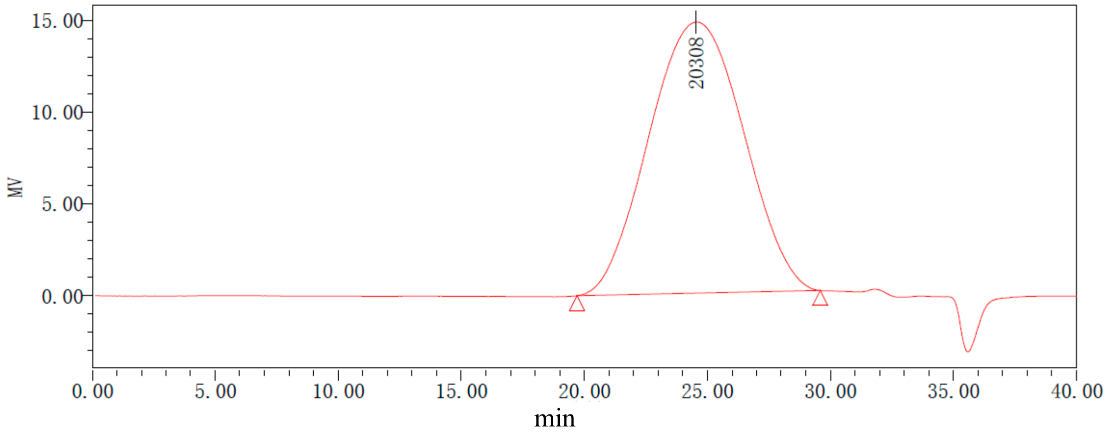

Table S29. GPC results.

M_n (D)	M_w (D)	MP	M_z (D)	$M_z + 1$ (D)	PDI
15746	26784	20308	43939	64006	1.701009

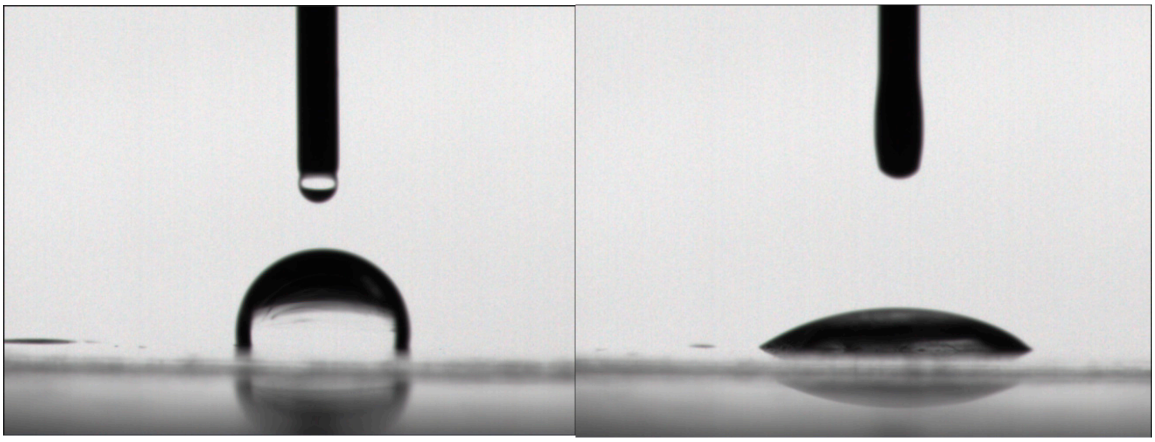

Figure S63. Contact Angle with Water and n-Hexadecane.

32. Compound of Poly(5-co-LMA) (1:1)

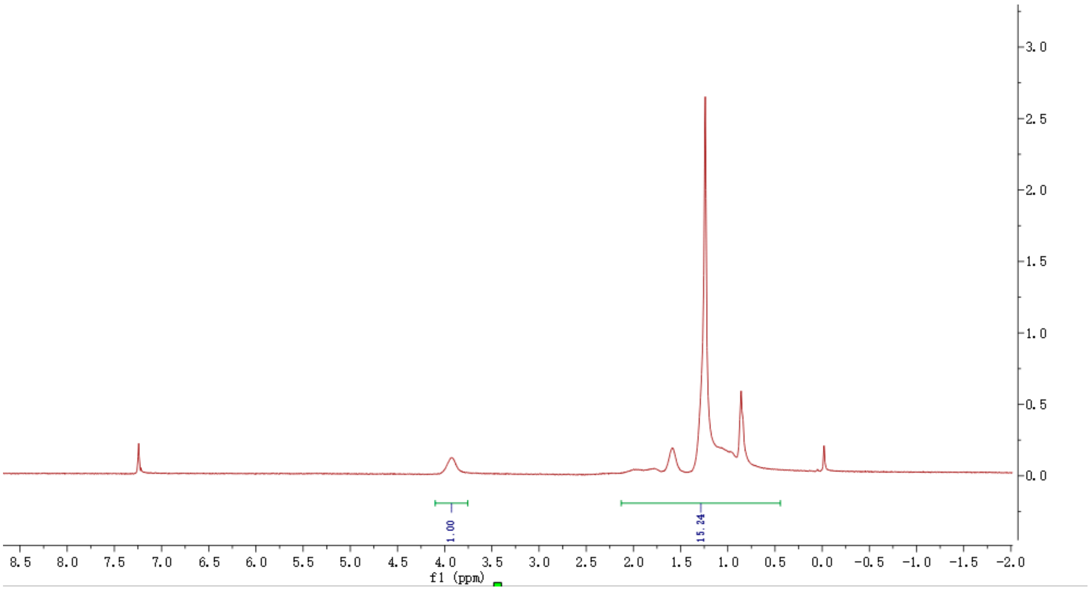

Figure S64. ^{1}H NMR ($CDCl_3$, 400 MHz).

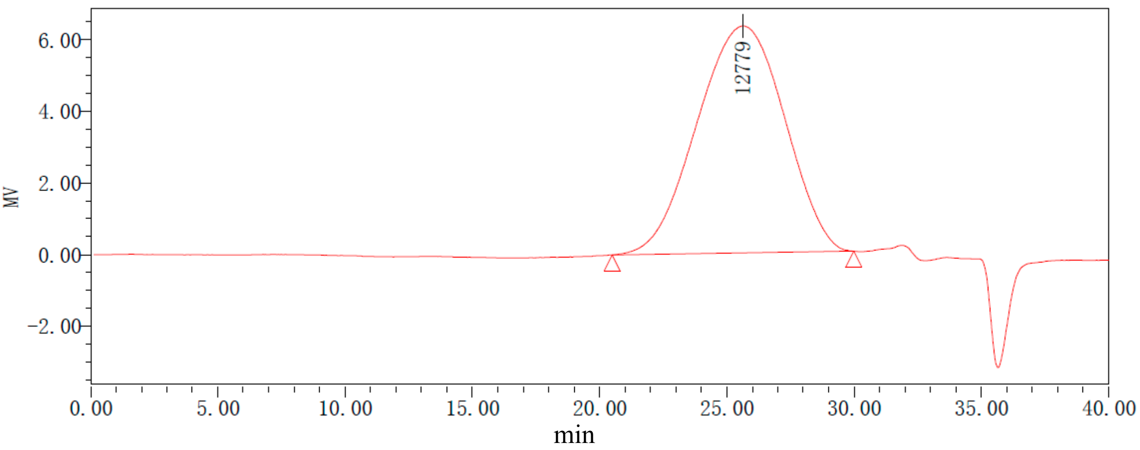

Table S30. **GPC results.**

M_n (D)	M_w (D)	MP	M_z (D)	$M_z + 1$ (D)	PDI
10982	16759	12779	25942	38398	1.526041

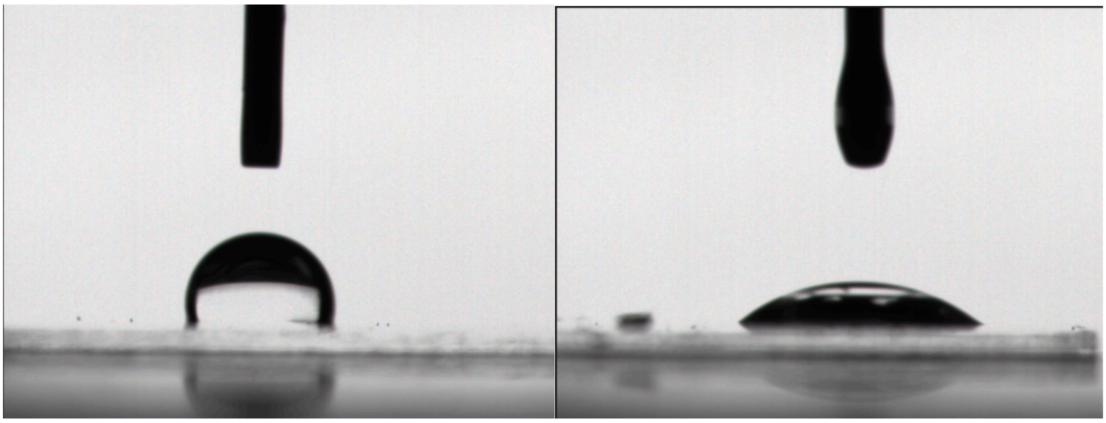

Figure S65. Contact Angle with Water and n-Hexadecane.

33. Compound of Poly(5-co-SMA) (1:15)

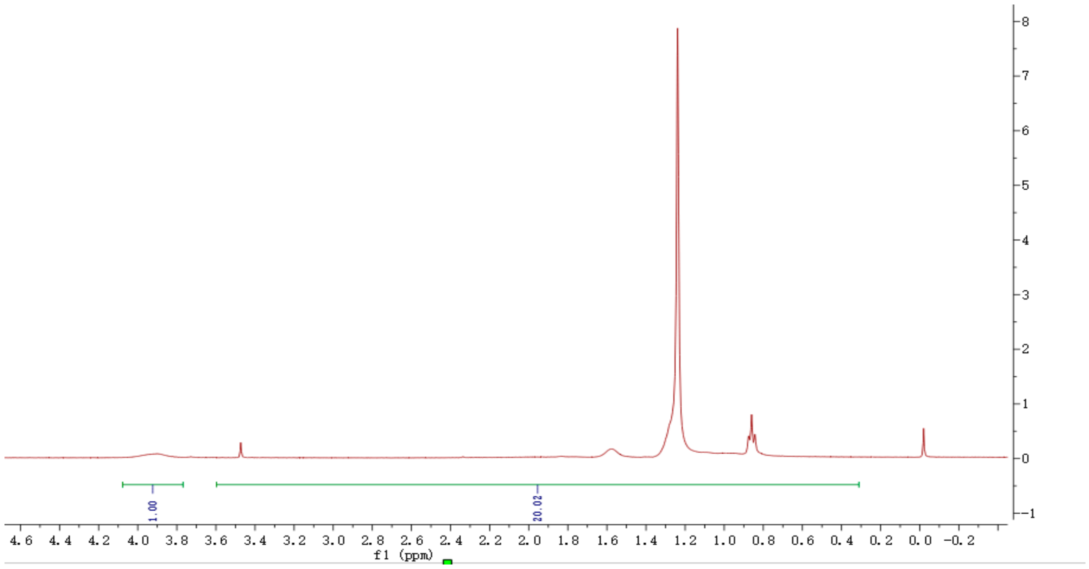

Figure S66. ^1H NMR ($CDCl_3$, 400 MHz).

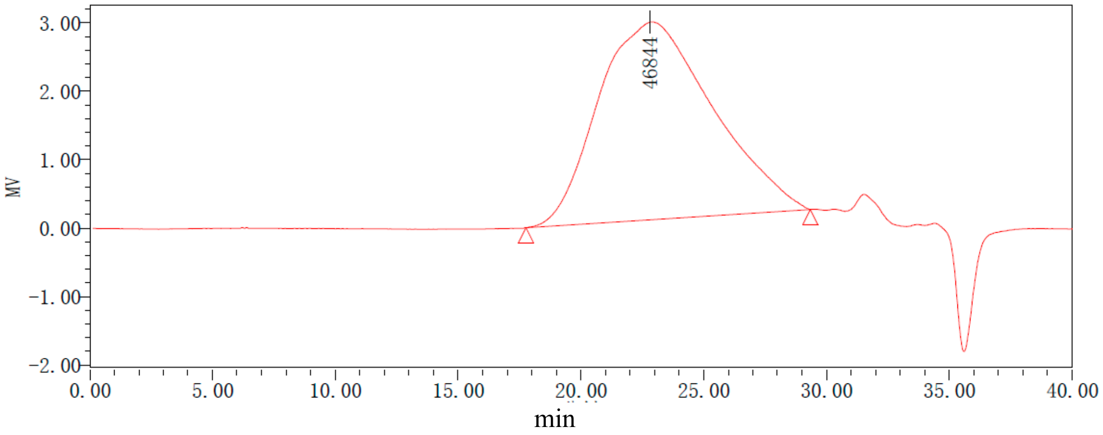

Table S31. GPC results.

M_n (D)	M_w (D)	MP	M_z (D)	$M_z + 1$ (D)	PDI
24,473	59,055	46,844	111,660	166,563	2.413054

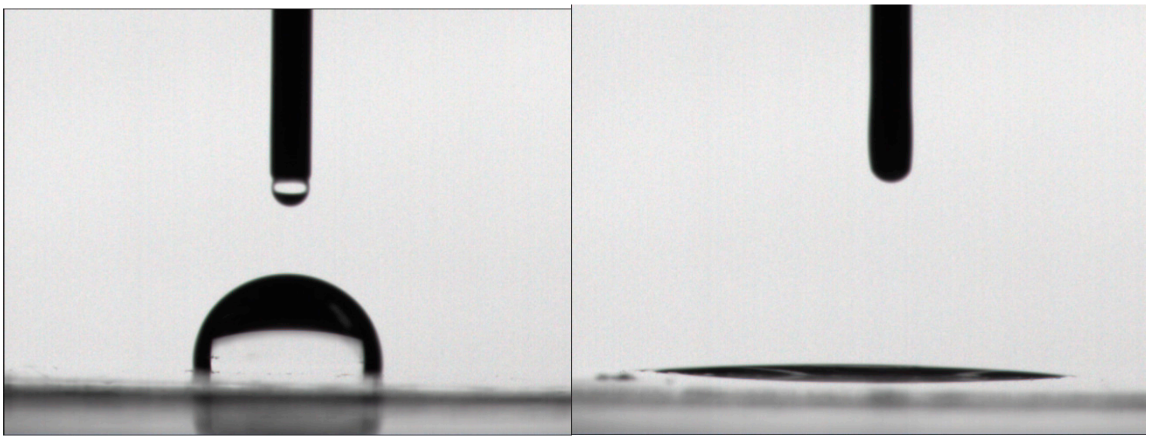

Figure S67. Contact Angle with Water and n-Hexadecane.

34. Compound of Poly(5-co-SMA) (1:8)

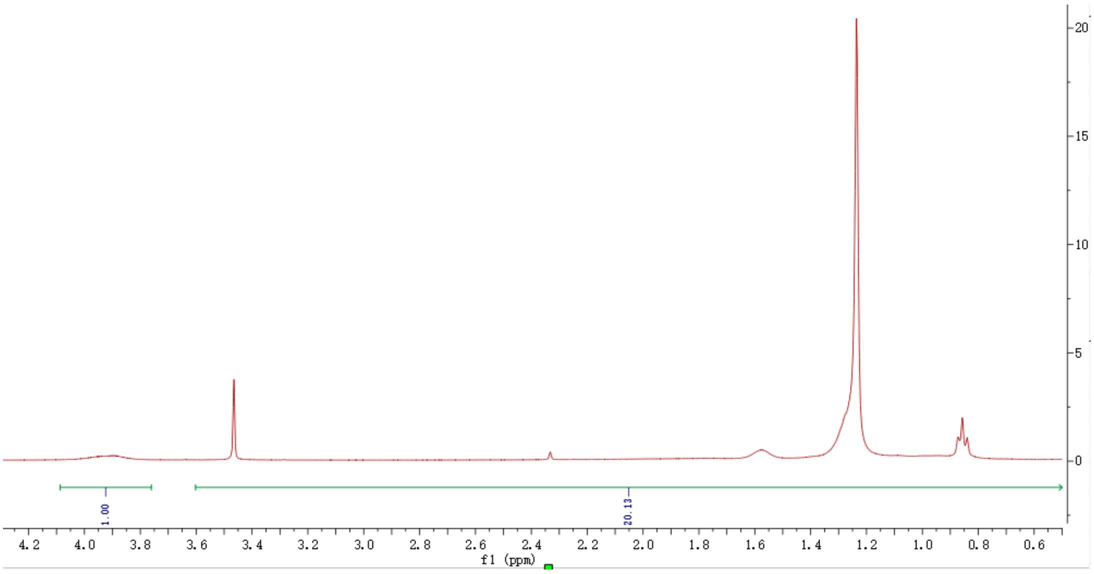

Figure S68. ^1H NMR ($CDCl_3$, 400 MHz).

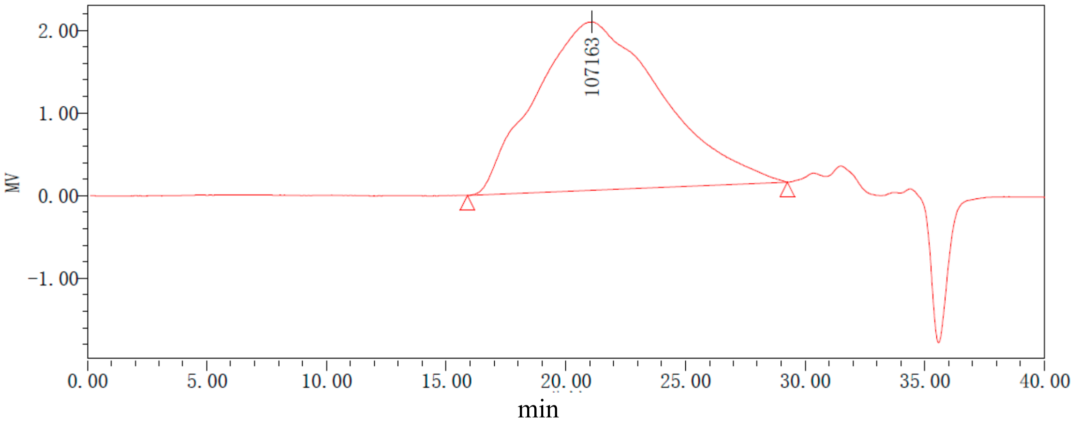

Table S32. GPC results.

M_n (D)	M_w (D)	MP	M_z (D)	$M_z + 1$ (D)	PDI
41346	156080	107163	401568	704109	3.774987

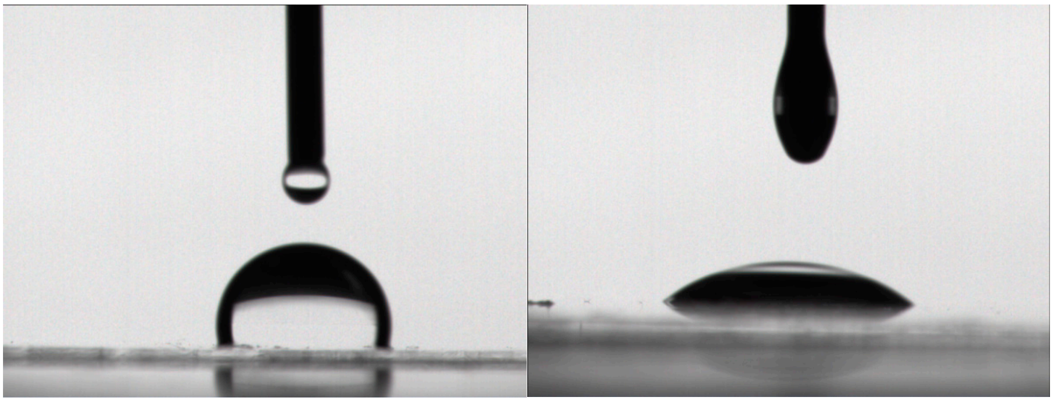

Figure S69. Contact Angle with Water and n-Hexadecane.

35. Compound of Poly(5-co-SMA) (1:6)

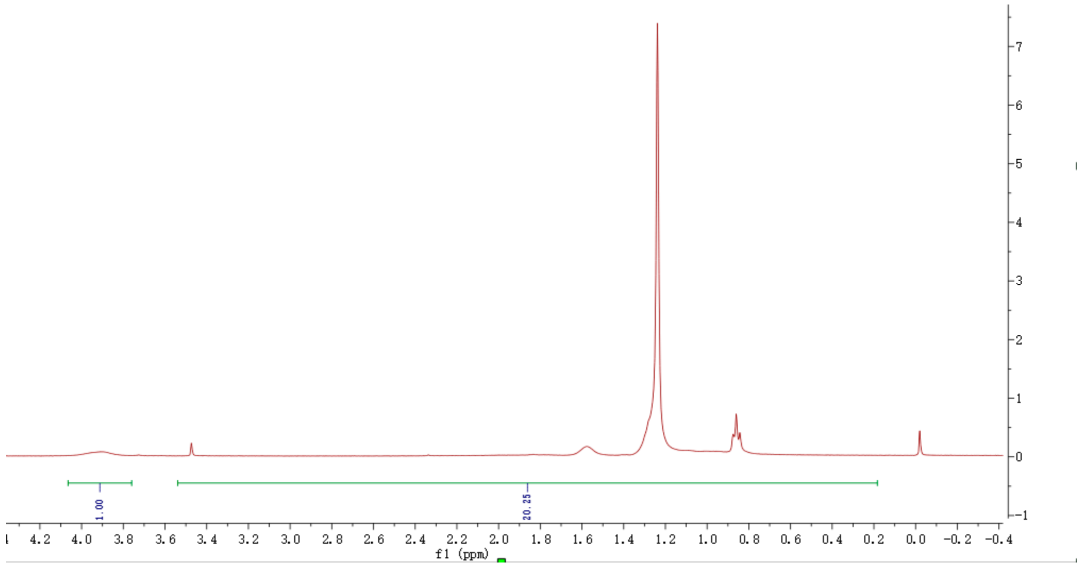

Figure S70. ^1H NMR ($CDCl_3$, 400 MHz).

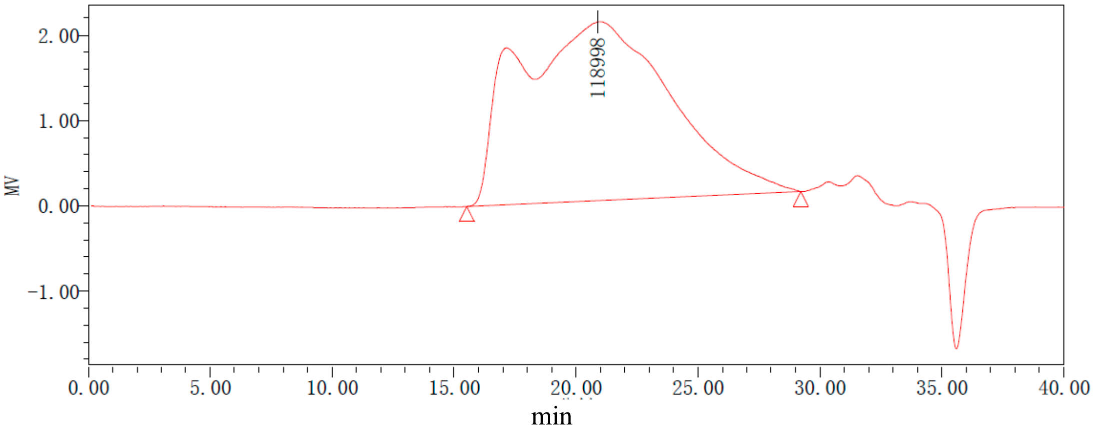

Table S33. GPC results.

M_n (D)	M_w (D)	MP	M_z (D)	$M_z + 1$ (D)	PDI
51258	282544	118998	814452	1282245	5.512205

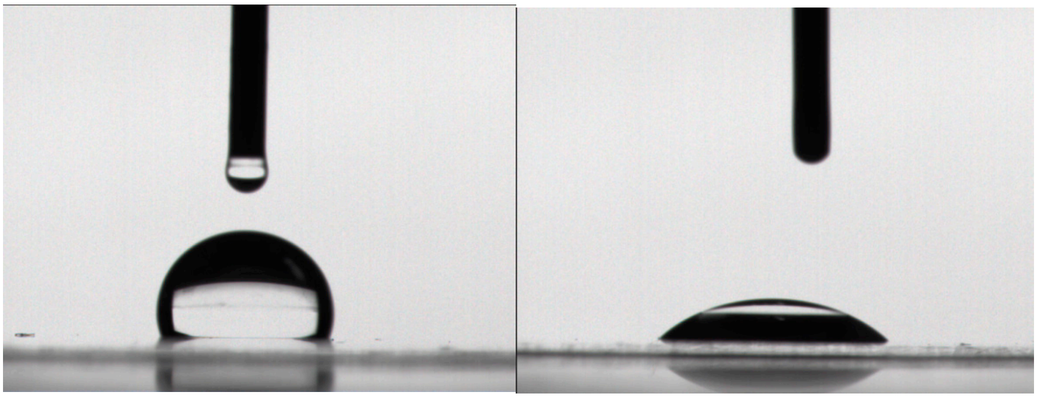

Figure S71. Contact Angle with Water and n-Hexadecane.

36. Compound of Poly(5-co-SMA) (1:2)

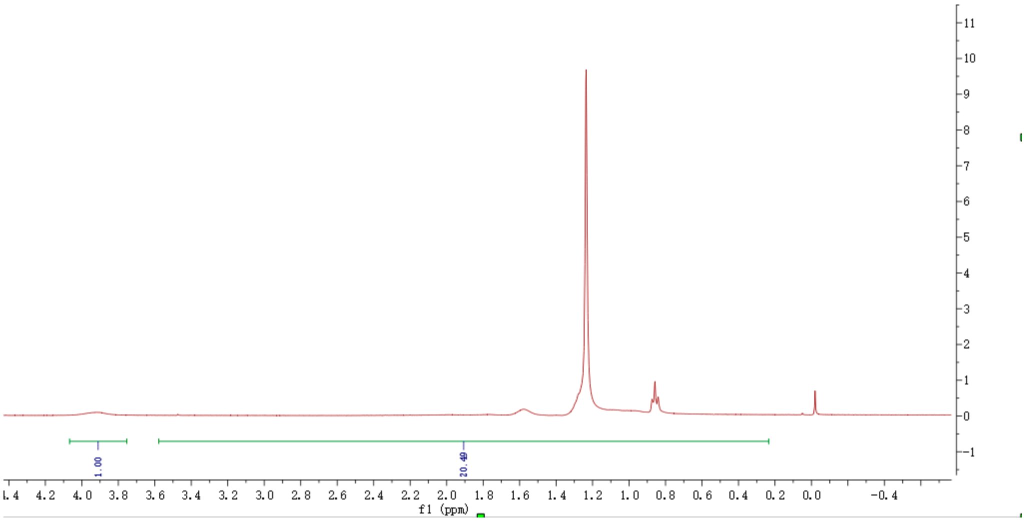

Figure S72. ^{1}H NMR ($CDCl_3$, 400 MHz).

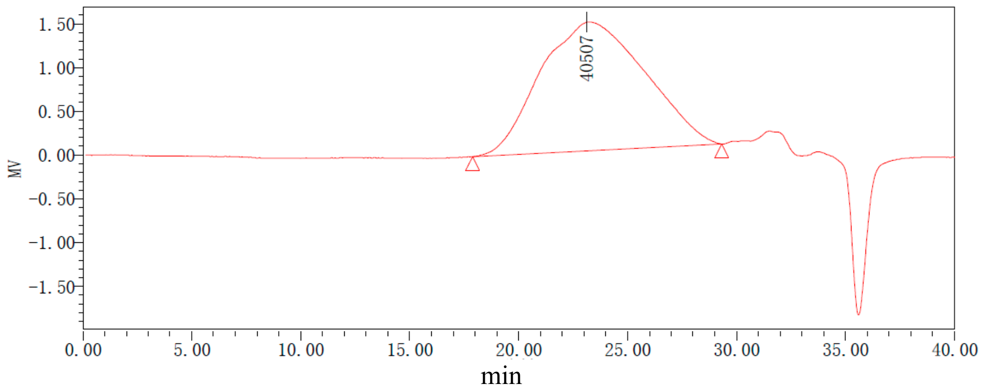

Table S34. GPC results.

M_n (D)	M_w (D)	MP	M_z (D)	$M_z + 1$ (D)	PDI
22274	52870	40507	104719	160683	2.373591

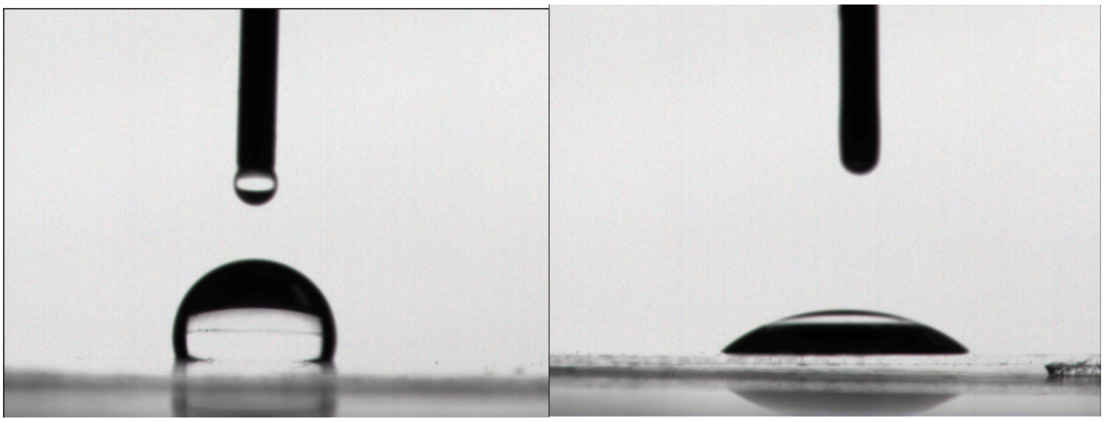

Figure S73. Contact Angle with Water and n-Hexadecane.

37. Compound of Poly(5-co-SMA) (1:1)

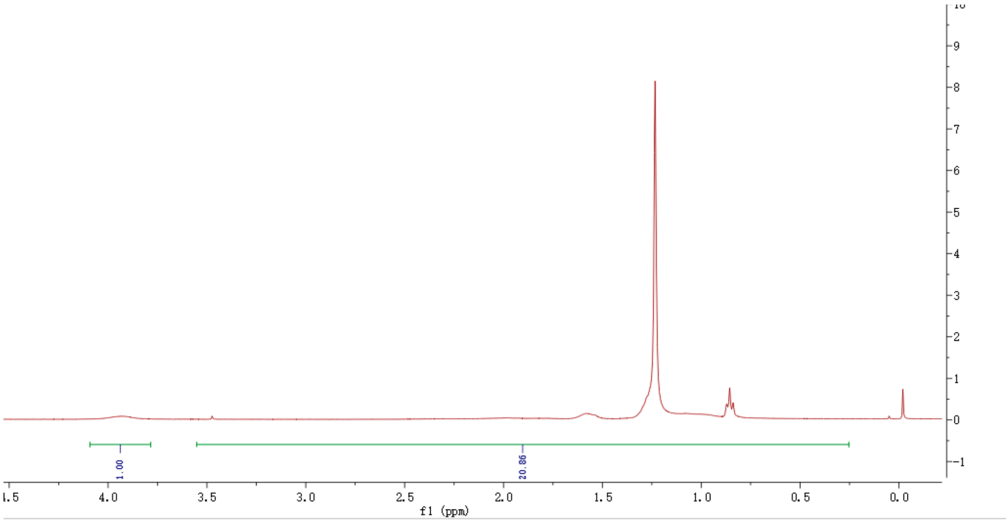

Figure S74. ^1H NMR ($CDCl_3$, 400 MHz).

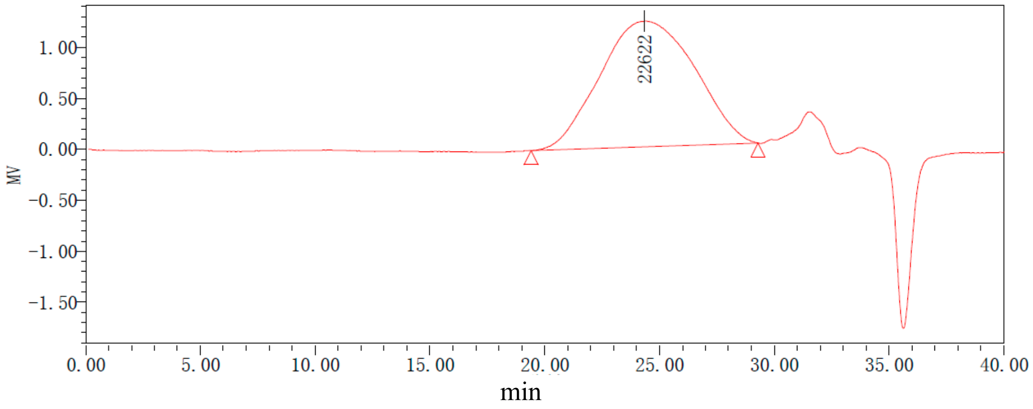

Table S35. GPC results.

M_n (D)	M_w (D)	MP	M_z (D)	$M_z + 1$ (D)	PDI
16315	29509	22622	51433	77091	1.808724

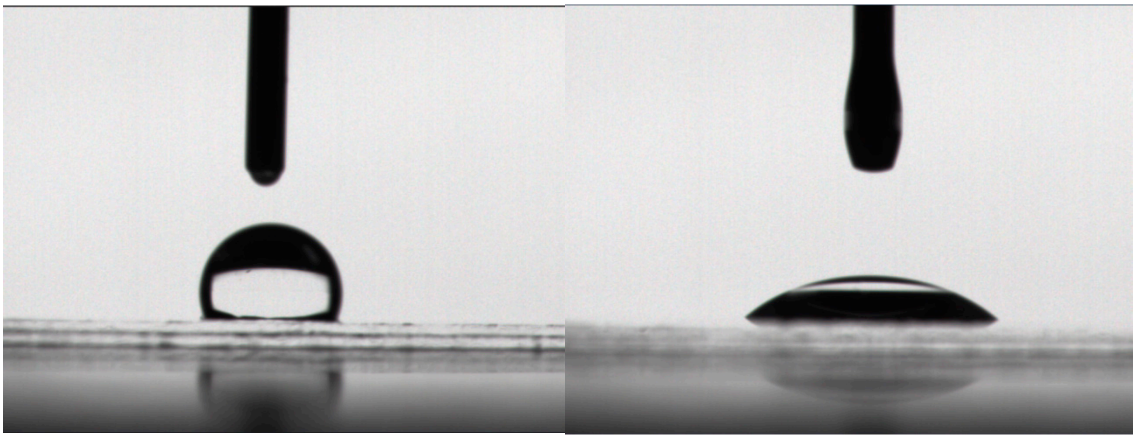

Figure S75. Contact Angle with Water and n-Hexadecane.
